# Identifying Terpenoid Biosynthesis Genes in *Euphorbia maculata* via Full-Length cDNA Sequencing

**DOI:** 10.3390/molecules27144591

**Published:** 2022-07-19

**Authors:** Mi Jin Jeon, Neha Samir Roy, Beom-Soon Choi, Ji Yeon Oh, Yong-In Kim, Hye Yoon Park, Taeyoung Um, Nam-Soo Kim, Soonok Kim, Ik-Young Choi

**Affiliations:** 1 Microorganism Resources Division, National Institute of Biological Resources, Incheon 22689, Korea; mj428star@korea.kr (M.J.J.); ohjy21@korea.kr (J.Y.O.); 2 Agriculture and Life Sciences Research Institute, Kangwon National University, Chuncheon 24341, Korea; neha_roy@kangwon.ac.kr (N.S.R.); tyoungum@kangwon.ac.kr (T.U.); 3 BIT Institute, NBIT Co., Ltd., Chuncheon 24341, Korea; bschoi319@gmail.com; 4 On Biological Resource Research Institute, Chuncheon 24239, Korea; yikim0524@gmail.com; 5 Biological Resources Assessment Division, National Institute of Biological Resources, Incheon 22689, Korea; cauapple@naver.com; 6 Department of Agriculture and Life Industry, Kangwon National University, Chuncheon 24341, Korea

**Keywords:** *Euphorbia maculata* L., medicinal plant, MVA pathway, MEP pathway, terpenoids, transcriptomes, PacBio SMRT sequencing

## Abstract

The annual herb *Euphorbia maculata* L. produces anti-inflammatory and biologically active substances such as triterpenoids, tannins, and polyphenols, and it is used in traditional Chinese medicine. Of these bioactive compounds, terpenoids, also called isoprenoids, are major secondary metabolites in *E. maculata*. Full-length cDNA sequencing was carried out to characterize the transcripts of terpenoid biosynthesis reference genes and determine the copy numbers of their isoforms using PacBio SMRT sequencing technology. The Illumina short-read sequencing platform was also employed to identify differentially expressed genes (DEGs) in the secondary metabolite pathways from leaves, roots, and stems. PacBio generated 62 million polymerase reads, resulting in 81,433 high-quality reads. From these high-quality reads, we reconstructed a genome of 20,722 genes, in which 20,246 genes (97.8%) did not have paralogs. About 33% of the identified genes had two or more isoforms. DEG analysis revealed that the expression level differed among gene paralogs in the leaf, stem, and root. Whole sets of paralogs and isoforms were identified in the mevalonic acid (MVA), methylerythritol phosphate (MEP), and terpenoid biosynthesis pathways in the *E. maculata* L. The nucleotide information will be useful for identifying orthologous genes in other terpenoid-producing medicinal plants.

## 1. Introduction

*Euphorbia* (Euphorbiaceae) is a genus of flowering plants with about 2000 species that is subdivided into many subgenera and sections [1,2]. Distributed worldwide from desert to temperate zones, *Euphorbia* species range from tiny annuals to large and long-lived trees (https://www.finegardening.com/genus/euphorbia; accessed on 1 December 2021). Many *Euphorbia* species are used in traditional Chinese, Japanese, and Korean medicine [3]. Shi et al. (2008) surveyed biomolecules in *Euphorbia* and identified 535 molecules among the terpenoids, steroids, phenolic compounds, and flavonoids [2]. Their biological activities include cytotoxicity, effects on cell division, DNA damage, tumor promotion, and antimicrobial activity [3,4]. *E. maculata* L., commonly called spotted spurge, is an annual herb native to North America but grows worldwide. Although the sap from *E. maculata* may cause skin irritation and rash in some people, extracts have been used to treat diarrhea, hemolysis, and hematuria [4]. There are numerous reports on the bioactive phytochemicals in *E. maculata*, such as polyphenols, tannins, flavonol glycosides, and triterpenoids [4,5,6,7].

Also known as isoprenoids, terpenoids are a large class of plant secondary metabolites with more than 50,000 naturally occurring members [8]. Terpenoids are organic compounds derived from a 5-carbon isoprene (C_5_) called isopentyl diphosphate (IPP). Terpenoids are synthesized by the head-to-tail addition of IPP (C_5_) units, resulting in hemiterpenoids (C_5_H_8_), monoterpenoids (C_10_H_16_), diterpenoids (C_20_H_32_), and triterpenoids (C_30_H_48_) [9]. There are two IPP biosynthesis pathways: the cytosolic mevalonic acid (MVA) pathway, resulting in IPP; and the plastidial methylerythritol phosphate (MEP) pathway, resulting in dimethylallyl diphosphate (DMAPP), an IPP isomer [10]. The cytosolic MVA pathway begins with 2-Acetyl-CoA, which is converted to IPP by stepwise enzyme-mediated reactions [11]; the plastidial MEP pathway starts with the condensation of pyruvate and glyceraldehyde-3-phosphate by 1-deoxy-D-xylulose-5-phosphate (DOXP) synthase. Then, DOXP is converted to DMAPP by stepwise enzymatic reactions [12]. The IPP and DMAPP isomers are interconverted by isopentyl pyrophosphate isomerase (IDI) [13]. While triterpenoids and sesquiterpenoids are synthesized via the MVA pathway, monoterpenoids, diterpenoids, and tetraterpenoids are synthesized via the MEP pathway [14].

Numerous reports document the terpenoids in *Euphorbia* species. Tsopmo and Kamnaing (2011) isolated 18 terpenoid molecules from whole plant parts of *E. sapinii* by simple acetone extraction and deciphered their molecular structures [15]. Terpenoids were extracted from *E. pedroi*, and an isolated tetracyclic triterpenoid was demonstrated to be a multidrug resistance reverser [16]. Many *Euphorbia* species produce a milky latex that is irritating to humans and animals. The triterpene alcohols derived from the milky latex of *E. azorica* have potential as chemopreventive and chemotherapeutic agents in cancer treatment [17]. Sun et al. (2018) isolated 17 triterpenoid derivatives including two lanostane-type triterpenoids from *E. maculata* [4]. The isolated triterpenes exhibited potent anti-inflammatory activities, and the authors proposed these triterpenes as candidate cancer chemopreventive agents. Terpenoids have pharmacological benefits, including antitumor, anti-inflammatory, antibacterial, antioxidation, and immunoregulation activities, and can be used in the prevention of cardiovascular diseases [18].

A transcriptome is the complete set of transcripts at a defined spatial and temporal stage of an organism’s life cycle, and it provides comprehensive information on gene expression and regulation [19,20]. Next-generation sequencing (NGS) technologies, such as Illumina paired-end transcriptome analysis [21,22] and single-molecule real-time sequencing (PacBio SMRT) technology, have been used to isolate numerous key genes in metabolite biosynthesis pathways [23,24]. The PacBio SMRT system is especially useful for plants lacking reference genome sequence data because it reads full-length transcripts [25,26,27]. Plant metabolites are often biosynthesized in specific tissues; thus, tissue-specific transcriptomes can be compared to identify key genes involved in various complex metabolite biosynthesis pathways in plants [28,29].

In this study, we characterized the terpenoid biosynthesis genes in *E. maculata*. We sequenced the leaf, root, and stem transcriptomes using Illumina short-read sequencing and PacBio SMRT techniques. The former technique allowed us to identify differentially expressed genes (DEGs) in the metabolite biosynthesis pathways, and the latter allowed us to obtain the complete sequences and isoform copy number information of transcripts involved in terpenoid biosynthesis.

## 2. Materials and Methods

### 2.1. Sample Preparation

Tissue samples (leaves, stem, and roots) of *Euphorbia maculata* were obtained from the experimental garden of Hallym University, Korea. The *E. maculata* accessions were originally collected in Kangwon Province of Korea. The collected tissues were immediately frozen with liquid nitrogen and stored at −80 °C until use.

### 2.2. Illumina RNA-Seq Library Construction and Sequencing

Total RNA was purified from leaves, stem, and roots using the RiboPure Kit (Applied Biosynthesis, Foster City, CA, USA). DNase1 (Sigma, St. Louis, MO, USA) was used for residual DNA digestion, and the total RNA was quantified using a NanoDrop 2000 spectrophotometer (Thermo Scientific, Wilmington, DE, USA). Paired-end sequencing was performed with a Nova Seq platform (Illumina, San Diego, CA, USA) at the professional sequencing provider Theragen Bio Co., Ltd. (Seongnam, South Korea). The quality of the constructed libraries was checked by a LabChip GX system (PerkinElmer, Waltham, MA, USA).

### 2.3. Full-Length cDNA Sequencing

Total RNAs from the three tissues (leaf, root, and stem) were pooled, and RNA quality was checked (Agilent Technologies, Santa Clara, CA, USA). The cDNA size selection was performed with a BluePippin system (Sage Science, Beverly, MA, USA) to build two cDNA libraries of ≤4 and ≥4 kb. Iso-Seq library preparation and sequencing were carried out using the PacBio full-length cDNA library and sequencing kit according to the manufacturer’s protocol (Pacific Biosciences Inc., San Diego, CA, USA) at the sequencing service provider Theragen Bio Co., Ltd. (Seongnam, South Korea).

### 2.4. De Novo Assembly and Iso-Seq Data Analysis Using a Bioinformatics Pipeline

PacBio raw sequencing reads were processed via the standard Iso-Seq protocol in SMRTlink 4.0 software. Polymerase reads shorter than 50 bp were removed, and the subread BAM files were set to error-corrected circular consensus sequences (CCSs) using the following parameters: full passes ≥0 and predicted consensus accuracy >0.75. Full-length (5′- and 3′-adapters and the poly-A tail) and non-full-length reads (CCSs with all 5′- and 3′-reads) were clustered into consensus sequences using the Iterative Clustering for Error Correction (ICE) algorithm (https://www.pacb.com/products-and-services/analytical-software; accessed on 1 April 2022). These reads were further combined with non-full-length transcripts and polished in clusters by Quiver [30].

### 2.5. Full-Length Unique Transcript Model Reconstruction

Error-corrected, high-quality (HQ) and low-quality (LQ), full-length, polished consensus transcripts were combined to remove redundancy using the CD-HITv4.6 package with the parameters –c 0.99 –G 0 –aL 0.00 –aS 0.99 –AS 30 –M 0 –d 0 –p 1 [31]. The non-redundant transcripts were processed with the Coding GENome reconstruction Tool (Cogent v7.0.0, https://github.com/Magdoll/Cogent; accessed on 1 April 2022). Cogent creates the *k*-mer profile of non-redundant transcripts, computes pairwise distance, and clusters the transcripts into families based on their *k*-mer similarity. Each transcript family was further reconstructed into one or several unique transcript models (referred to as UniTransModels) using a De Bruijn graph method.

### 2.6. Isoform and Paralog Identification

Error-corrected, non-redundant transcripts (transcripts before Cogent reconstruction) were mapped to UniTransModels using Minimap2 v2.6 (Li 2018). Splicing junctions for transcripts mapped to the same UniTransModels were examined, and transcripts with the same splicing junctions were collapsed using Cupcake ToFU v13.0.0 [25]. Collapsed transcripts with different splicing junctions were identified as transcription isoforms of UniTransModels. Paralogs were analyzed by the BLASTclust program with the unigene sequences (https://www.ncbi.nlm.nih.gov/Web/Newsltr/Spring04/blastlab.html; accessed on 1 April 2022) with a score coverage threshold of 1.75 and a length coverage threshold of 0.9.

### 2.7. Functional Annotation

**Figure 1:**
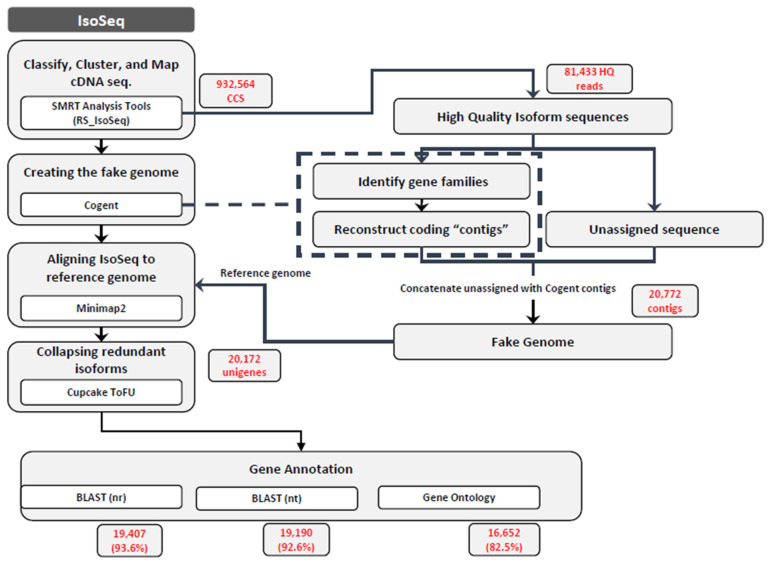
Schematic representation of full-length cDNA analysis in *E. maculata*.

Functional annotations were obtained by mapping sequences into several databases. Non-redundant protein sequences (Nr) and non-redundant nucleotide sequences (Nt) were compared against the NCBI database by BLAST v2.10.1 with an E-value cut-off of 1 × 10^−5^. Gene Ontology (GO) analyses were carried out by BLAST2GO v5.2.5 (bioinformatics software) with an E-value cut-off of 1 × 10^−5^. Figure 1 shows the genomics and bioinformatics pipeline used in this study.

### 2.8. Differential Gene Expression Analysis

Illumina reads were aligned using Bowtie 2 v2.4.2 [32]. The read count values were directly obtained and converted to fragments per kilobase of transcript per million mapped reads (FPKM) values using RSEM (v1.1.12) [33]. Then, the DEGs between different tissue samples (leaf vs. stem, leaf vs. root, and stem vs. root) were detected with the standardization trimmed mean of M values (TMM) normalization method using edge R [34]. The significant DEGs were screened at false discovery rates (FDRs) < 0.05 and fold change of 2 as a cut-off.

## 3. Results

### 3.1. E. maculata Transcriptome Analysis Using PacBio Iso-Seq

**Figure 2:**
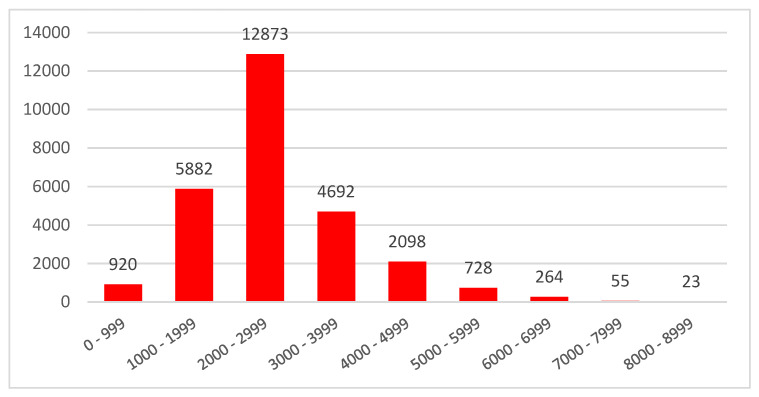
Length distribution of the transcripts after de novo assembly.

We clustered raw sequencing reads from the full-length cDNA libraries into consensus transcripts using the TOFU pipeline (GitHub version) supported by PacBio (Table 1). We obtained approximately 62 million polymerase reads with an average length of 56,777 bp in the ≤4 kb library and 51,584 bp in the ≥4 kb library. We obtained 467,479 CCSs with an average length of 2471 bp and a CCS read score of 0.989 in the ≤4 kb library and 465,085 CCSs with an average length of 4040 bp and a read score of 0.983 in the ≥4 kb library. Using the standard Iso-Seq protocol for transcript clustering, we obtained 47,860 high-quality (HQ) isoforms and 405 low-quality (LQ) isoforms in the ≤4 kb library and 33,573 HQ and 993 LQ isoforms in the ≥4 kb library (Table 1). Then, we processed 81,433 HQ transcripts with the COding GENome reconstruction Tool (Cogent v7.0.0, https://github.com/Magdoll/Cogent; accessed on 1 April 2022) to develop a fake genome of 20,722 reads (containing 18,481 reconstructed contigs and 2241 unassigned sequences). The fake genome was then used as a reference to map the HQ transcripts, which produced 20,172 isoforms (Figure 1, Table 2). The transcript length showed a normal distribution with the greatest number of transcripts in the 2000–2999-bp range (Figure 2).

### 3.2. Isoforms and Paralogs

**Figure 3:**
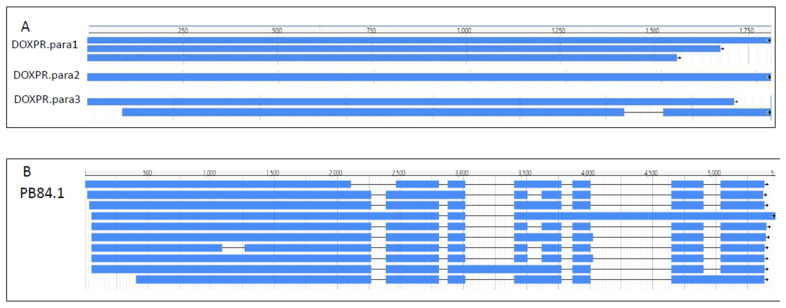
Paralogs and isoforms. (**A**): *DOXP* had three paralogs: DOXP.para1, DOXP.para2, and DOXP.para3. DOXP.para1 had three isoforms with different translation termination sites. DOXP.para3 had two isoforms due to alternative splicing and differences in translation initiation and termination sites. (**B**): PB84.1 is a tRNA ligase gene. It had no paralogs, but 10 isoforms, which differed by alternative splicing and different translation initiation and termination sites.

Of the 20,172 unigenes, 13,492 (66.9%) had no isoform (singleton), while the remaining 6680 unigenes had 2–25 isoforms, and 19.6% of the unigenes produced two isoforms (Table 2). Most unigenes (20,246 unigenes, 97.8%) did not have paralogs (Table 3). The remaining 475 unigenes had 2–20 paralogs. Figure 3 shows the isoforms and paralogs of the DOXP synthase gene and the tRNA ligase gene.

### 3.3. Functional Annotation

Of the 20,172 unigenes, 19,190 (95.1%) and 19,407 (96.2%) matched with the non-redundant nucleotide sequence (Nt) and non-redundant protein sequence (Nr) databases, respectively, in NCBI. Of the unigenes matched to the Nr database, the highest match was with *Hevea brasiliensis* (6046; 29.9%), followed by *Jatropha curcas* (4477; 22.1%), *Ricinus communis* (4048; 20%), and *Manihot esculenta* (3452; 17.6%).

**Figure 4:**
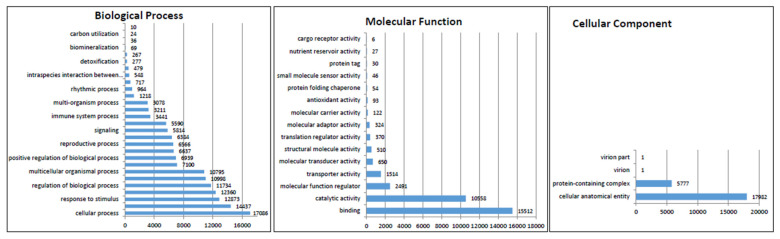
GO analysis of the *E. maculata* transcripts.

In the functional classification, we assigned Gene Ontology (GO) terms to each of the UniTransModels via the BLAST2GO program based on the annotation of the Nr database. Overall, 16,652 (82.55%) unigenes were classified into three major categories: ‘biological process’, ‘molecular function’, and ‘cellular component’ (Figure 4). Genes in the biological process category primarily fell into seven major subgroups with over 10,000 transcripts: cellular process (GO: 00099871), metabolic process (GO: 0008152), response to stimulus (GO: 0050896), biological regulation (GO: 0065007), regulation of biological process (GO: 0050789), developmental process (GO: 0032502), and multi-multicellular organism process (GO: 0044706). In the molecular function category, two subgroups, binding (GO: 0005488) and catalytic activity (GO: 0003824), were predominant. Genes fell mainly into two subgroups in the cellular component category: cellular anatomical entity (GO: 0110165) and protein-containing complex (GO: 0032991).

### 3.4. Gene Expression Analysis across Different Tissues

**Figure 5:**
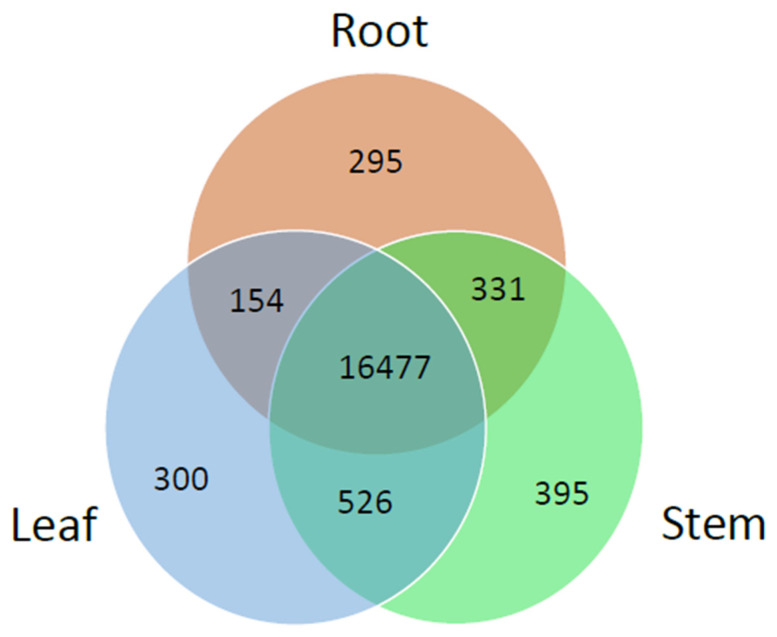
Venn diagram showing the number of unigenes expressed in three different organs.

**Figure 6:**
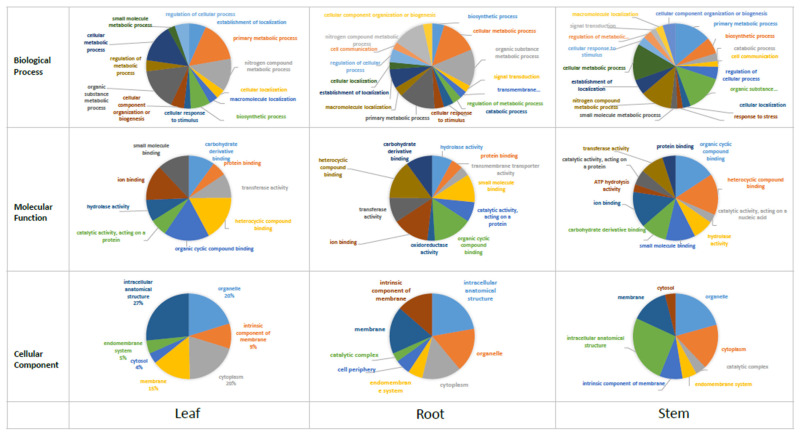
GO analysis of the organ-specific-expressing unigenes.

We analyzed the DEGs in leaf, root, and stem tissues by mapping the Illumina sequencing reads to the Pac-Bio unigene reference sequences (Table 4). The percent mapped paired-end reads to unigene reference sequences was 70.9, 60, and 64.8 in the leaf, root, and stem, respectively. The number of expressed genes was 17,735, 17,260, and 18,008 in the leaf, root, and stem, respectively. Of the 20,172 unigenes, 16,477 (81.7%) were expressed constitutively among the three organs. There were 295 organ-specific genes in the root, 300 in the leaf, and 395 in the stem (Figure 5). The number of DEGs with more than a two-fold difference in expression was distinct among the three organs. We identified more upregulated genes in the root than in the shoot or stem (Table 4). Figure 6 shows the GO analysis of the organ-specific genes. In the biological process category, the proportion of genes involved in metabolic processes was higher in the aboveground organs (leaf and stem) than in the root. However, the distribution of genes in the molecular function and cellular process categories was similar among the three organs.

### 3.5. Terpenoid Biosynthesis Pathway Genes

**Figure 7:**
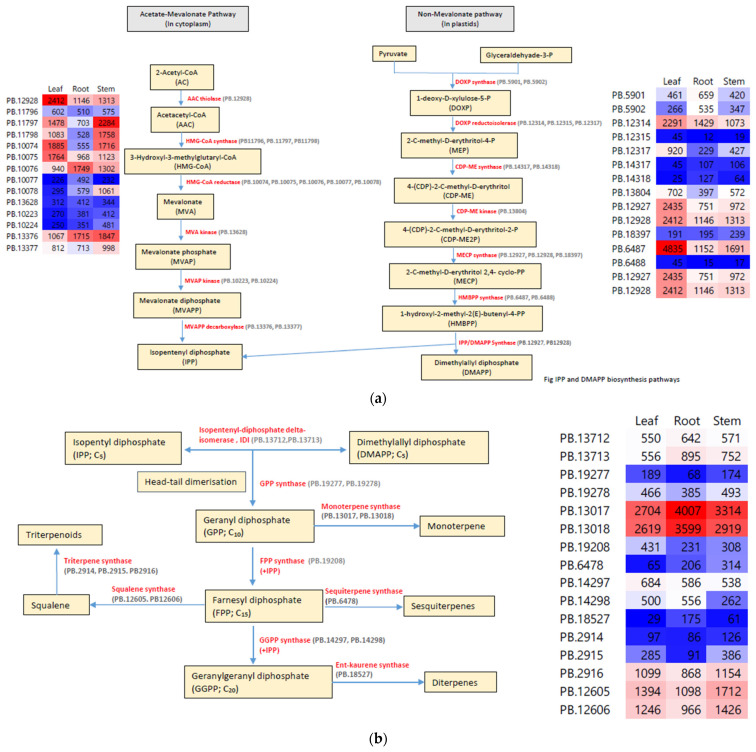
Biochemical pathways of (**a**) the MVA and MEP pathways and (**b**) terpenoid biosynthesis. The numbers in parenthesis are the genes in the *E. maculata* transcriptomes. The numbers in the heat maps are the FPKM-normalized values.

We identified all genes in the MVA, MEP, and terpenoid biosynthesis pathways (Table 5; Figure 7). The nucleotide sequences of paralogous genes and isoforms in these pathways are listed in Supplementary Table S1. In the MVA pathway, six genes encode the enzymes involved in IPP biosynthesis, with one (AAC thiolase and MVA kinase) to five (HMG-CoA reductase) paralogs per gene and one to three isoforms of each paralog. The first reaction in the MEP pathway is the condensation of pyruvate with glyceraldehyde 3-phosphate to form DOXP by DOXP synthase. The DOXP synthase gene had two paralogs and one and three isoforms of each paralog. There are five genes involved in the conversion of DOXP to 1-hydroxyl-2-methyl-2(*E*)-butenyl-4-diphosphate (HMBPP), which had one (CDP-ME synthase) to five (HMG-CoA reductase) paralogs and one to three isoforms of each paralog. HMBPP is reduced to dimethylallyl diphosphate or IPP by IPP/DMAPP synthase, which has two paralogous genes with only one isoform each. IPP and DMAPP are isomers that are interconverted by IDI. IDI has two paralogous genes with one and three isoforms. IPP undergoes head-to-tail dimerization to form geranyl diphosphate (GPP) by GPP synthase, which has two paralogous genes with a single isoform each. GPP is converted to monoterpenes by monoterpene synthase, which has two paralogous genes with a single isoform each. GPP is also converted to farnesyl diphosphate (FPP) by farnesyl synthase, which is encoded by a single-copy gene with two isoforms. FPP is processed into sesquiterpenoids or squalene by sesquiterpene synthase or squalene synthase, respectively. Squalene is further processed to triterpenoid by triterpene synthase, which is encoded by three paralogous genes with a single isoform each. Geranylgeranyl diphosphate (GGPP) is converted into diterpenes by diterpene synthase, which is annotated as ent-kaurene synthase. Ent-kaurene synthase is encoded by a single-copy gene with one isoform.

In a single gene, different paralogs had different numbers of isoforms as exemplified by the DOXP gene in Figure 3. DOXP.para1 had three isoforms with different termination sites, and DOXP.para3 had two isoforms with different starting and termination sites, as well as different exons. The expression of the paralogs differed among the tissues (Figure 7). For instance, of the five paralogs of the gene encoding HMG-CoA reductase in IPP biosynthesis, PB.10074 had the highest expression in the leaf and the lowest expression in the root, but PB.10076 had the opposite expression pattern. Supplementary Table S1 shows the sequence information of all the genes involved in the terpenoid synthesis in *E. maculata*.

## 4. Discussion

NGS technologies have revolutionized many areas of genetics. Transcriptomics captures a snapshot of the total transcripts in a cell at a specific time and is used to quantify gene expression profiles during development [19,35]. High-throughput short RNA-Seq analysis was used to identify the genes involved in the biosynthesis of phytochemicals in medicinal plants [25,27,36]. Here, we used transcriptome profiling to analyze the genes involved in terpenoid biosynthesis in the medicinal plant *E. maculata* L., which is used in folk medicine in oriental countries [4]. Terpenoids are major secondary metabolites in *E. maculata* that have pharmacological benefits including anti-inflammation, antioxidant, antitumor, hepatoprotection, and anti-HIV protease activity [4,5,7,37].

The *E. maculata* genome has not been sequenced; therefore, we obtained transcriptome sequences from PacBio SMRT full-length cDNA sequencing. We obtained 20,172 full-length unigenes, which is similar to that obtained in *Berberis koreana* (23,246) by PacBio SMRT sequencing [27]. Although full-length unigenes may not accurately represent the number of genes in a species, the number of genes in *E. maculata* may be low compared to other plant species. Gene numbers in plants range from 20,000 to 124,000. The small genome of *Arabidopsis thaliana* encodes 26,000 genes [38]. We previously reported an Illumina NovaSeq-derived transcriptome of *Euphorbia jolkini* having 123,215 assembled transcripts [27]. In our functional annotation of *E. maculata* genes, 19,190 (92.6%) and 19,407 (93.65%) matched with the Nt and Nr databases in NCBI, respectively, indicating that the function of most of the transcripts is known and only about 7% of the transcripts have not been annotated. The top three species BLAST-matching with *E. maculata* transcripts were the Pará rubber tree (*Hevea brasiliensis*), castor bean (*Ricinus communis*), and cassava (*Manihot esculenta*), all in Euphorbiaceae. These plants produce a milky latex containing terpenes [18,39,40]. The high match to these species may be because they have well-characterized transcriptome data due to their economic importance, as reported in the Pará rubber tree [41,42], castor bean [43], and cassava [44,45]. GO allows the comparison and functional classification of genes and their products across species (http://www.geneontology.org/; accessed on 1 April 2022) and covers three domains: cellular components, molecular functions, and biological processes. In our *E. maculata* transcriptomes, the distribution of genes in the different functional categories was similar to that of other medicinal plants [26,27,46].

PacBio SMRT sequencing is a third-generation sequencing system that allows the identification of isoforms [20,47]. Paralogs are homologous genes in a species that arise from the duplication of a single ancestral gene [48]. We identified isoforms and paralogs in our PacBio SMRT sequencing data. In humans, approximately 70% of protein-coding genes have at least one paralog [49]. *Arabidopsis* has at least 21,843 paralogs, which account for approximately 84% of its protein-coding genes [50]. However, 97.8% of the *E. maculata* unigenes were single copy, which is unexpectedly high because most eukaryotes underwent several whole-genome duplication events that resulted in the duplication of ancestral genes. Thus, it will be interesting to determine the number of paralogs in other *Euphorbia* species to verify our findings. Currently, only one *Euphorbia* transcriptome has been reported, but it was generated by Illumina NovaSeq, which does not permit the analysis of paralogs of full-length transcripts [26]. Transcript isoforms are derived from alternative splicing of the introns and the differential initiation or termination of translation from primary transcripts, which allows a single gene to code for multiple forms of a protein [51]. Proteome plasticity from alternative splicing plays a major role in adaptation to environmental stresses [52]. In plants, alternative splicing occurs in about 24% of transcripts in wheat (*Triticum aestivum*) to 60% in *Arabidopsis* in intron-containing genes [44]. In the *E. maculata* transcriptome, about 35.8% of the unigenes had isoforms; two examples are shown in Figure 3. Different paralogs had different isoform patterns. Furthermore, the expression patterns of paralogs differed among root, stem, and leaf tissues. Thus, paralogs and their isoforms might help plants adapt to stresses, as demonstrated in cassava under cold stress [44].

Terpenoids are the major bioactive compounds in *E. maculata*. We isolated the genes, as well as their isoforms and paralogs, involved in the MVA, MEP, and terpenoid biosynthesis pathways in *E. maculata*. The MVA pathway begins with Acetoacetyl-CoA synthase (AAC thiolase), which catalyzes the condensation of two 2-Acetyl-CoA (AAC) molecules. AAC is subsequently transformed into five intermediate molecules to form IPP, which involves five enzymes: HMG-CoA synthase, HMG-CoA reductase, MVA kinase, MVAP kinase, and MVAPP decarboxylase (Figure 7) [11]. In *E. maculata*, the genes encoding these enzymes were present as single-copy up to five-copy genes, with one to three isoforms per gene (Table 4). HMG-CoA reductase is a key regulatory enzyme in the MVA pathway in plants [53] and catalyzes the conversion of HMG-CoA to MVA, which is a rate-limiting step in the MVA pathway [10,13]. The HMG-CoA reductase gene is highly conserved among organisms, and we identified 1929 HMG-CoA reductase mRNAs among all biological kingdoms from viruses to bacteria to eukaryotes in the NCBI database (data not shown). The gene encoding HMG-CoA reductase had five copies in *E. maculata*, and each paralog was expressed differently in stem, leaf, and root tissue. Developmental and organ-specific expression of the HMG-CoA reductase gene was also reported in plants [53]. The HMG-CoA reductase gene was expressed higher in stems than in roots and leaves in lavender (*Lavandula pubescens*), which also produces terpenoids [54]. In *E. maculata*, one of the HMG-CoA reductase-paralogous genes was highly expressed in stems. The various paralogs expressed differently among the three organs, which may be highly coordinated for plant development.

The MEP pathway, also known as the non-mevalonate (non-MVA) pathway [13], occurs in plastids; thus, animals do not have this pathway, which has spurred interest as a potential strategy to develop anti-bacterial or herbicide products [55,56]. We identified all enzyme-encoding genes of the MEP pathway in *E. maculata*. Except for the gene encoding CDP-ME kinase, all other enzyme-encoding genes had two to four copies and several isoforms. IPP derived from the MVA pathway and DMAPP derived from the MEP pathway are structurally unrelated isomers that are interconverted by IDI. Because IPP is derived directly from the MVA pathway, IDI is not essential for plant survival; thus, IDI may play a role in modulating the IPP/DMAPP ratio in the cell [13].

IPP is a C_5_ molecule that undergoes enzyme-mediated sequential head-to-tail condensation to become GPP (C_10_), FPP (C_15_), and GGPP (C_20_) [12]. There were two, one, and two copies of GPP synthase, FPP synthase, and GGPP synthase in *E. maculata*, respectively. GPP is converted to monoterpenes by monoterpene synthase, which was encoded by two paralogous genes, and both copies had very high expression in the three organs in our analysis. Monoterpenoids have not been reported in *E. maculata*, but several monoterpenoid compounds were reported in other *Euphorbia* species [57,58]. FPP is converted to sesquiterpenes (C_15_) by sesquiterpene synthase or squalene (C_30_) by squalene synthase. We found one copy of the sesquiterpene synthase gene in *E. maculata*. A sesquiterpene synthase gene was isolated from *Euphorbia fischeriana*, which produced several sesquiterpenoids, including cedrol and eupho-acorenols [59,60]. Oxygenated sesquiterpenes and sesquiterpene hydrocarbons were identified in different *Euphorbia* species, and their bioactivities were also reported [3]. Squalene (C_30_) is a precursor of steroids [61]. Squalene is biosynthesized by combining two molecules of FPP by squalene synthase. A squalene synthase gene was isolated from *Euphorbia pekinensis* [62] and *Euphorbia tirucalli* [63]. We found two copies of squalene synthase in *E. maculata*, and both copies were actively expressed in the three organs. Squalene is converted to triterpenoids (C_30_) by triterpenoid synthase, also called oxidosqualene cyclase [64]. A triterpene synthase gene was isolated from the bark of *Euphorbia lathyris*, in which triterpenoids are abundant [63]. The terpene synthase gene was highly expressed in the latex of *E. lathyris*. We identified three copies of the triterpene synthase gene in *E. maculata*, and their expression was high in leaves and stems compared to roots. Sun et al. (2018) reported two new triterpenes from dried whole *E. maculata* plants, which had anti-inflammatory properties [4]. Triterpenes have been isolated from diverse *Euphorbia* species [63,64,65]. Diterpenoids (C_20_) are derived from GGPP by diterpene synthase. Diterpenoids are abundant in *Euphorbia* species [60]. We found one copy of the diterpene synthase gene in *E. maculata*. Plants produce thousands of diterpenoids, and diterpene synthases have numerous functions in diverse plants [66].

## 5. Conclusions

*E. maculata* L. is a medicinal herb that produces bioactive compounds including terpenoids. We conducted transcriptome sequencing via PacBio SMRT and Illumina RNA-Seq to identify the genes involved in terpenoid biosynthesis in *E. maculata*. Because the *E. maculata* genome sequence is not available, we used de novo assembly and obtained 20,722 unique full-length transcripts. PacBio SMRT sequencing allowed us to identify paralogous genes and isoforms. GO and DEG analyses revealed that paralogs of each gene expressed differently in stem, leaf, and root tissues. Using this approach, we identified the genes involved in the terpenoid biosynthesis pathway in *E. maculata*. Our sequence information will be useful for isolating orthologs in other terpenoid-producing medicinal plants.

## Figures and Tables

**Table 1:** PacBio summary of RNA-seq data from two RNA libraries of *E. maculata*.

Analysis Metric	Under 4 kb	Over 4 kb
**Polymerase reads**		
Total Polymerase Read length (bp)	31,143,923,142	31,036,246,900
Total Polymerase Reads	548,527	601,659
Average Polymerase Read Length (bp)	56,777	51,584
**Subreads**		
Total Subreads	18,525,814	8,597,836
N50	2504	3893
Average Subread Length (bp)	1630	3739
**Circular consensus sequence (CCS) reads**		
Total CCS reads	467,479	465,085
Total CCS read length (bp)	1,155,280,061	1,879,756,017
Average CCS read length (bp)	2471	4040
**Transcript clustering**		
Number of polished high-quality isoforms	47,860	33,573
Number of polished low-quality isoforms	405	993

**Table 2:** IsoSeq results and statistics of isoforms in the transcriptomes of *E. maculata*.

Iso Seq Result	Number of Reads	Length (bp)
High-quality consensus Seq.	76,631	216,086,311
Reconstructed Coding Contig	19,902	60,494,776
Unassigned Seq	3344	10,608,597
Fake Genome	20,722	71,103,373
Minimum read length		100
Maximum read length		13,544
Average read length		3059
Number of Isoforms	Number of Transcripts	Percentage (%)
1	13,492	66.9
2	3946	19.6
3	1269	6.3
4	630	3.1
5	381	1.9
6	185	0.9
7	116	0.6
8–25	153	0.8
Total	20,172	100

**Table 3:** Distribution of number of paralogs in the transcriptome of *E. maculata*.

Number of Paralogs	Number of Transcripts
1	20,246
2	84
3	14
4	18
5–20	27

**Table 4:** Mapping information of the Illumina sequence reads and the results of differentially expressed genes.

Mapping Information	Leaf	Root	Stem
No. of total reads	25,971,888	29,095,594	26,009,774
No. of mapped Paired-end reads	18,411,506	17,458,816	16,843,542
% Mapped Paired-end reads	70.9	60	64.8
No. of expressed genes			
0	2987	3642	2714
>0	17,735	17,260	18,008
Differential Expression	Leaf vs. Root	Root vs. Stem	Leaf vs. Stem
Up	447	1049	87
Down	1660	177	266

**Table 5:** Enzymes involved in the biosynthesis of terpenoids, isopentyl diphosphate, and dimethylallyl diphosphate.

Enzymes	Abbreviation	Pathway	No of Paralogs	Range of Isoform
		Acetate-Mevalonate		
Acetoacetyl CoA thiolase	AAC thiolase		1	1
3-Hydroxy-3-methylglutaryl synthase	HMG-CoA Synthase		3	1
3-Hydroxy-3-methylglutaryl reductase	HMG-CoA Reductase		5	1–3
Mevalonate kinase	MVA kinase		1	1
Mevalonate phosphate kinase	MVAP kinase		2	1–2
Mevalonate diphosphate decarboxylase	MVAPP carboxylase		2	1–2
		Non-Mevalonate		
1-deoxy-D-xylulose-5-phophate synthase	DOXP synthase		2	1–3
1-deoxy-D-xylulose-5-phophate reductoisomerase	DOXP reductoisomerase		3	1–3
Cytidine diphosphate 2-C-methyl-D-erythritol synthase	CDP-ME synthase		2	1
Cytidine diphosphate 2-C-methyl-D-erythritol kinase	CDP-ME kinase		1	1
2C-methyl-D-erythritol synthase	MECP synthase		4	1
1-hydroxy-2-methyl-2-D-butenyl-4-diphosphate synthase	HMBPP synthase		2	2
IPP/MDAPP synthase	IspH		2	1
		Terpenoid synthesis		
Isopentenyl-diphosphate delta-isomerase	IDI		2	1–2
Geranyl diphosphate synthase	GPP synthase		2	1
Farnesyl diphosphate synthase	FPP synthase		1	2
Geranyl geranyl diphosphate synthase	GGPP synthase		2	1
Monoterpene synthase	Monoterpene synthase		2	1
Sesquiterpene synthase	Sesquiterpene synthase		1	1
Diterpene synthase	Ent-Kaurene synthase		1	1
Squalene synthase	Squalene synthase		2	1
Triterpene synthase	Triterpene synthase		3	1

## Data Availability

The raw data of the current research are linked in the http://nbitglobal.com/emaculata (accessed on 12 April 2022).

## Supplementary Materials

The following supporting information can be downloaded at: https://www.mdpi.com/article/10.3390/molecules27144591/s1, Table S1: The DNA sequence information of all the genes involved in the terpenoid synthesis in *E. maculata*.

## Sample Availability

Samples of the compounds are available from the authors.
