# Supplementary material for: Identifying Terpenoid Biosynthesis Genes in Euphorbia maculata via Full-Length cDNA Sequencing
Source: Molecules. 2022 Jul 19;27(14):4591. doi: 10.3390/molecules27144591 (PMC9316252; doi:10.3390/molecules27144591)
Supplement: Supplementary file 1 [file molecules-27-04591-s001.zip › molecules-1786616-supplementary.pdf]

MVA pathway genes
>PB.12928.1 Euphorbia maculata AAC thiolase mRNA
ACAATAAAAACCCAAAGCCATAATCACAAAAAGGATCTCTTCAGCTGAGTCCACTGGTTTTGTTTCCCCCTCCGC
CCATTTGACCACCGTCTCTTGTCTTCTCCGGCGAGCCATGGCCATTTCTCTCCAACTCTGCCGCCTCTCCCTCCG
CGCCGACCTCTTTTCCGGCGACAAGCGCGTCCCTTGTTTCCCCCGTAAATCGATCTCCATCCGCTGCTCCGCCGG
CGACTCCGCTTCGCCGTCGGTCTCCGGTGACTCCGATTTTGATGCTAAGGTATTCCGGCACAACTTGACTCGGAG
CAAGAATTACAACCGGAGAGGATTCGGACACAAGGAGGAGACCTTGGAGCTCATGAATCAGGAATATACGAGTGA
TATTATCAAGACTTTGAAGGAAAACGGGAACGAATACACTTGGGGAAATGTTACTGTGAAATTGGCTGAAGCTTA
TGGGTTTTGCTGGGGCGTTGAACGCGCTGTGCAGATTGCTTATGAAGCTAGAAAGCAATTTCCTGAAGATAAGAT
TTGGATTACTAATGAAGTTATCCATAATCCGACTGTTAATCAGCGTTTGGAGGAGATGGAAGTTGTAACTATCCC
TGTTGCGGAAGGGGGAAAGCAATTTGAAGTTGTGAACAAAGGAGATGTTGTTATTTTGCCTGCTTTTGGAGCTGC
GGTAGACGAGATGTTGACTCTAAGTCAAAAGGATGTTCAAATTGTCGATACTACTTGCCCTTGGGTATCTAAGGT
CTGGAATACGGTTGAGAAGCACAAGAAGGGAGACTACACTTCAATAATTCATGGTAAATACAGTCACGAGGAAAC
TATTGCTACTGCTTCTTTTGCAGGAAAGTACATCATAGTGAAGAATATGGATGAGGCGATGTATGTTTGTGATTA
CATTCTTGGTGGTCAACTTAATGGTTCTAGCTCAACAAGAGAAGCTTTCCTTAAGAAATTTGAAAAAGCAGTTTC
CAAGGGATTTGATCCCGATGTTGACCTTGTCAAAGTCGGTATTGCAAATCAAACTACAATGCTCAAGGGAGAAAC
AGAAGCCATCGGAAAATTGGCGGAGAAGACCATGATGCAGAAATATGGTGTCGAAAATGTCAACGATCACTTTAT
TAGCTTCAACACCATCTGTGATGCTACTCAAGAGAGGCAAGATGCTATGTATAAATTGGTAGAAGACAAGCTGGA
TCTGATTCTAGTTGTTGGTGGATGGAACTCAAGCAACACTTCACACCTCCAAGAAATTGCAGAAGAGCGTGGAAT
TCCTTCATATTGGGTTGACTCTGAAAAGAGAATCGGTCCAGGAAACAAAATCGCTTATAAATTGAATCACGGGGA
ATTGGTTGAGAAAGAGAACTTCCTGCCAGAAGGCCCCATTACAATCGGTGTAACATCCGGTGCCTCAACTCCAGA
CAAGGTTGTTGAAGATGCGCTTGTGAAAATATTCGAAATCAAACGTCAAGAATTGTTGCAATTGGCATAAATGCG
TAAAATTAACAGAAGATTCTTAAGATCTGTGTTGTACCATCAGAATGTACAAAAGGAAGAACAATAATGCCGATA
ATGTGTTTGTTATTTCAGCTTATCTTGTCTTAGAACTAATGTAAAATCCCGAATAAGGTAGAGTCCGAAATTCAG
AGTACTAGCAAACAATCTCATGAATTGGTGTTGCATTCTTTGGTTAAAAAATTTCATATATATGCCTTTACTGT

>PB.11796.1 Euphorbia maculata HMG-CoA synthase Para.1 mRNA
AAACACAGAAATTGGTTTGTCTATATAAGCTCAACAGCCTTCATTTGGGTCCTCATCGTCCCCTTTCTCTTCGCA
GTTTTAATCCTATATTCTCTCCTCCATTCATTCCAAAGGAGCAAAGAGGATCAGCCGTGGTTTTTCCGGTTCACT
TTGGAAATTGTTTTCAATTTGATTGGAATTTTTCAGTAGCGAAGAGAAATGGCAAAGAATGTGGGAATTCTCGCT
ATGGATATCTATTTTCCTCCATCCTGCGTTCAGCAGGAAGCGCTGGAGAATCATGATGGAGTGAGCAAAGGGAAA
TACACTATTGGGCTTGGACAAGATTGCATGGCATTTTGTGCTGAGGTTGAAGATGTCATTTCAATGAGCTTGACA
GTTGTTAGTTCACTTCTCGACAAGTATAATATTGACCCCAAGCAAATCGGTCGATTGGAAGTTGGAAGTGAAACT
GTGATTGACAAGAGCAAATCTATTAAGACATTTCTGATGCAAATTTTTGAGAAACACGGGAATACTGACATAGAA
GGTGTTGACTCAACTAATGCTTGTTACGGAGGAACTGCAGCATTATTCAACTGTGTTAATTGGGTTGAGAGTCGT
TCTTGGGATGGACGATATGGACTTGTGGTGTGCACTGACAGTGCGGTATATGCAGAAGGTCCTGCTCGACCTACC
GGGGGAGCTGCAGCCATTGCTATGTTAATAGGTCCGGATGCTCCTATTGTTTTTGAAAGCAAACTCAGGGGCAGT
CATATGTCTCATGCCTATGACTTTTACAAGCCCAACCTTGCCAGTGAATATCCGGTTGTGGATGGTAAACTTTCT
CAAACTTGCTATCTGATGGCTCTTGATTTATGCTACAAATATTTCTGCACCAAGTATGAGAAATTTGAGGGCAAA
CAATTCTCTATTTCGGATGCTGACTATTTTGTGTTTCATTCACCTTATAACAAGCTTGTACAGAAAAGTTTTGCT
CGTTTGGTGTTCAATGATTTTGTTAGGAATGCAAGGTCTATAGACGAGGCAGCTAAAGAGAAACTTGCTCCATTT
TCCACTTTATCTGGTGATGAAAGCTACCAAAACAGAGATCTTGAAAAGGTGTCCCAACAAGTTGCCAAGCCCTTG
TATGATTCAAAGGTGCAACCATCGACTCTGGTACCAAAGCAAGTTGGGAACATGTACACTGCATCTATTTATGCA
GCATTTATATCCCTTCTTCACAACAAAAACACCGAATTGGCAGGAAATCGAGTGATAATGTTCTCATATGGGAGT
GGTTTGACAGCCACAATGTTCTCACTGCGCCTGAACGAAGGCCGGGGGCCTTTTAGCCTGTCAAACATCTCAACT
GTGATGAACATCACTGGAAAGTTGAAGGCAAGACATGAGGTGACCCCAGAGAAGTTTATTGAGACATTGAAGCTA
ATGGAGCATCGGTATGGGGCTAAAGACTTTGAAACGAACAAGGACTGCAGCCTCTTGGCCCCGGGGACTTACTAT
CTGACACATGTTGACAACATGTATCGGAGATTCTATGACATTAAATCTGTTGACAAAACTGGGGAGAATGGTCAC
ATTGGAAATGGGACTCACTGAGTGCAGGTGGATTATGGAGATGAGATGCGTCAGCAAATTAAGGTCTGCTATGGT
TTGAAGTGTTTTGTTGTCATATTGTTCATCAGCATGAGTTCCCCGTTGTGATTTCTATTTCTCTCCATAGAATTC
CTTTCTAGATTTCAGGTAGACTTTATTTGTTATGATCGATCAAAGTACTTGGGAAATTCAGTTTTGTTTGGTTCT
CTATATACAAAGGCTGTTTTTTAGCATAATTATTACCATTCCTCAGCAAATGTTTCTTATTTAGTTTTTTTTTC
TTATTGTAC

>PB.11797.1 Euphorbia maculata HMG-CoA synthase Para.2 mRNA
ATCGTCCCCTTCTCTTCGCTGCTTTACAGGCTTTGCAGTTTCTCGTCTCTGTTTTTCTCCTCCATTTTCAACGAA

AAGCGGGGAGAAACGAGGAAGAACTGTGTTTTGTTTCCTGTTTTCCTTTTCGAATTTGATCGGTTTTTGCTAGCT
AAGAGAAATGGCGAATAATGTGGGGATTCTCGCTATGGACATCTATTTTCCTCCTACCTGCGTTCAGCAGGAAGC
ACTGGAGAATCACGATGGGGTGAGCAAAGGGAAATATACCATCGGACTTGGACAAGATTGCATGGCATTTTGTAC
TGAGGTTGAAGATGTGATTTCAATGAGCTTGACAGTTGTTAGTTCGCTTCTCCAAAAGTACAATATTGACCCCAA
ACAAATCGGTCGATTGGAAGTTGGAAGTGAAACTGTGATTGACAAGAGTAAAGCGATCAAGACATTCTTGATGCA
GATTTTTGAGAAACACGGGAACACTGACATTGAAGGTGTTGATTCAACTAATGCTTGTTATGGAGGAACCGCAGC
TTTATTCAACTGTGTCAATTGGGTTGAGAGCCGTTCTTGGGATGGACGCTATGGACTTGTGGTTTGCACTGACAG
TGCGGTATACGCCGAAGGTCCTGCCCGACCCACTGGAGGAGCTGCAGCTATTGCTATGCTAATAGGCCCAGATGC
TCCTATTGTTTTTGAAAGCAAACTTAGGGGCAGTCACATGTCACATGCATATGACTTTTACAAGCCTAACCTTGC
CAGTGAATATCCGGTGGTGGATGGGAAGCTTTCGCAAACATGCTATCTCATGGCTCTTGATTCATGCTACAAATA
TTTCTGCGCCAAATACGAGAAATACGAGGGCAAACAATTCTCTATTTCTGATGCAGACTATTTTGTGTTTCATTC
TCCTTATAACAAGCTTGTACAGAAGAGTTTTGCTCGTTTGGTGTTCAATGACTTTGTTAGAAATGCAAGATCTGT
AGATGAGGCAGCTAAAGAGAAGTTTGCTCCATTTTTGAATTTATCTGGTGATGAAAGCTACCAAAGCAGAGATCT
CGAAAAGGTATCTCAACAAGTTGCCAAGCCTTTATATGATTCAAAGGTGCAACCAGCAACTCTGGTACCAAAGCA
GATTGGAAACACATACACTGCATCTATATATGCAGCATTGTTATCCCTCCTTCACAATAAAAACACTGAACTGGC
AGGAAAGCGGGTGATAATGTTCTCATATGGAAGTGGTTTGACATCCTCCATGTTCTCACTGCGCGTAAACGAAGG
CCACGATTCTTTTACCTTGTCAAATATTGCAACTGTGATGAACGTCGGTGGAAAGTTGAAGGCGAGACACGAGGT
CGCACCGGAGAAGTTCATAGAAACTTTGAAGCTAATGGAGCACAGATATGGGGGTAAAGACTTCGTAACAAGCAA
GGATTGCAGCCTCTTGGCACCTGGAACATACTATCTTACCGAAGTGGACAGCATGTACCGAAGATTTTATGACAT
TAAATCTGTTGACAAAACCGGTGAGAATGGTCACATTGCAAACGGTACTCACTGAGCTCGGATGGGTTATAGAAG
TGTAATATGTCACATCTACTATGGGTTGAAGTGTTGTTGAGTCCTAATGGACAGTTGTGTGTTGCTTTTAGTATG
GTTGTATTTTGTAGCCACGTCGTTTGATCATCGGCCTCATTGTATTTTGTTTCTCTTTTAATAAAATTTCTTTTA
AATTG

>PB.11798.1 Euphorbia maculata HMG-CoA synthase Para.3 mRNA
TCATTTCAATGAGCTTGACAGTTGTTAGTTCGCTTCTCCAGAAGTACAATATTGACCCCAAACAAATCGGTCGAT
TAGAAGTTGGAAGTGAAACTGTGATTGACAAGAGTAAAGCAATCAAGACATTCTTGATGCAAATTTTTGAGAAAC
ACGGGAACACTGACATTGAAGGCGTTGACTCATCTAATGCTTGTTATGGAGGAACCGCGGCTTTATTCAACTGTG
TCAATTGGGTTGAGAGCCGTTCTTGGGATGGACGCTATGGACTTGTGGTTTGCACTGACAGTGCGGTATACGCCG
AAGGTCCTGCCCGACCCACTGGAGGAGCTGCAGCTATTGCTATGCTAATAGGCCCAGATGCTCCTATTGTTTTTG
AAAGCAAACTTAGGGGCAGCCACATGTCACATGCATATGACTTTTACAAGCCTAACCTTGCCAGTGAATATCCGG
TGGTGGATGGGAAGCTTTCACAAACATGCTATCTCATGGCTCTTGATTCATGCTACAAATATTTCTGTGCCAAAT
ACGAGAAATATGAGGGCAAACAGTTCTCTATCTCTGATGCTGACTATTTTGTGTTTCATTCACCTTATAACAAGC
TTGTACAGAAGAGTTTTGCTCGTTTAGTGTTCAATGACTTTGTTAGGAATGCAAGATCCGTAGATGAGGCAGCTA
GAGAGAAGTTTGCCCCATTTTTGAATTTATCTGGTGATGAAAGCTACCAAAACAGAGATCTTGAAAAGGTATCCC
AACAAGTTGCCAAGCCCTTGTATGATTCAAAGGTGCAACCAGCAACTCTGGTACCAAAGCAGATTGGAAACACCT
ACACTGCATCTATATATGCAGCATTGCTGTCCCTTCTTCACAATAAAAACACTGAATTGGCAGGAAAGCGTGTGA
TAATGTTCTCATATGGAAGTGGTTTGACATCCTCAATGTTCTTACTGCGCCTAAACGAAGGCCATGATTCGTTTA
GCTTGTCAAATATTGCAACTGTGATGAATGTCGGTGGAAAGTTGAAGGCAAGGCACGAGGTCGCACCGGAAAAGT
TCATAGAAAATTTGAAGCTAATGGAGCACAGATATGGGGGCAAAGACTTTGTAACAAGCAAGGATTGCAGCCTCT
TGGCACCCGGAACATACTATCTTACCGAAGTTGACAGCATGTATCGGAGATTTTATGACATTAAATCTGTTGACA
AAAGCGGTGAGAACGGTCACATTGCCAATGGTTCTCACTGAGTGCAGATGGGTTATAGATGTGTAATATGTCAGC
TCTACTATGGTTTGAAGTGTTGTTGAGTCCTAATGGACAGTTGTGTGTTACTTTTAGTATGGTTGTATTTTGTAG
CCACGTCGTTTGTTCTTCAGCGAGAGTTCCCCATTGCAATTTTTTTCTCTTTTAATAAAATTTCTTTTGAATTGC

>PB.10074.1 Euphorbia maculata HMG-CoA reductase Para.1 mRNA
AAAATTAAAAGCATCGTCACCTTTGGAGCAGAAAGACGCACATGTGTGGACATGAAGCTAAAGGTGGGAGCTTGG
ATCATATAAAAATGTCCCAAATCTCCCAGAGAACTCCACCCCTCTCCTTCATATCACCACCTCCCTCCCCCCCCT
TCTCCACATTCCTCCTCCCATGGAGACTCAACCGCGGCGCCCCAAGCACCTGCCTAACGCCAACCCCAAGCACCT
CCTCCAGCCCCCCCTCAAGGCCTCCGACGCCCTCCCCCTCCCCCTCTACCTCACCAACGCCGTCTTCTTCTCCCT
CTTCTTCTCCCTCGCCTACTTCCTCCTCCACCGCTGGCGCGACAAGATCCGCTCCTCCACCCCCCTCCACCTCCT
CTCCCTCTCCGAGATCGTCGCCCTCGTCTCCCTCATCGCCTCCTTCATCTACCTCCTCGGCTTCTTCGGCATCGA
CTTCGTCCAGTCCTTCATCGCCCGCCCCGACGACCGCGATTACCTCATCAACGGCGACCACCGCCTCCTCACTTG
CTCTCCCCCCAAAATCAACGCCGTTCCCAAAATGCCACCCCCGGAGCCGATAATATCCCCCCTGGCCTCCGCGGA
GGACGAGGAGATCGTGAAATCGGTCGTGAACGGCACGATTCCGTCGTATTCTCTCGAATCGAAGCTCCGCGATTG
CAAGCGCGCGGCGGCGATTCGGCGGGAGGCGCTGCAGAGGACGATGGGGAGGTCGCTGGAGGGTTTGCCGGTTGA

CGGATTCGATTACGAGTCGATTTTAGGTCAGTGCTGCGAAATGCCGGTGGGATACGTGCAGATTCCGGTGGGAAT
CGCGGGGCCGTTGTTGCTCGACGGAAGAGAGTACTCTGTTCCCATGGCCACCACCGAGGGCTGCTTGGTTGCGAG
TACTAACAGAGGATGCAAAGCGATTCATCTCTCCGGCGGCGCCAGCAGTGTGCTGTTGAAGGACGGCATGACTCG
AGCTCCGGTGGTTCGGTTCGGGTCGGCGACAAGGGCGGCGGAATTGAAGTTTTTCCTCGAGAATCCTGACAATTT
CGATAGCTTGTCGGTCGTTTTTAACAGGTCCAGTAGATTTGCAAAGCTTCAAGGAATACAGTGCGCGATTGCTGG
TAAAAATCTGTACATTAGATTCAGCTGCAGCACTGGCGATGCAATGGGGATGAACATGGTTTCCAAAGGGGTCCA
AAACGTTCTCGAATTCCTTCTGAGTGATTTCCCTGATATGGATGTTATTGGTATCTCTGGGAATTTCTGCTCGGA
CAAGAAGCCAGCTGCAGTGAACTGGATCGAAGGGCGAGGCAAATCGGTGGTTTGCGAGGCAATAATCAAGGAAGA
AGTGGTAAAAAAGGTGTTGAAAACAAGCGTTGCTTCACTAGTAGAGCTGAACATGCTCAAGAATCTCACAGGATC
AGCCGTTGCTGGATCTCTCGGGGGATTCAATGCACACGCAGGCAACATAGTCTCTGCAATATTCATCGCCACCGG
CCAAGATCCAGCCCAGAATGTGGAGAGTTCCCACTGCATCACCATGATGGAGGCCGTCAACGACGGCAGGGATCT
CCACATCTCTGTAACCATGCCTTCCATCGAGGTGGGGACAGTTGGAGGGGGGACTCAGCTTGCATCGCAATCGGC
TTGCCTGAATCTGCTCGGTGTGAAGGGGGCGAGCAAGGAGTCGCCAGGGGCAAACTCGAGGCTCCTGGCGAGCAT
CGTGGCTGGATCGGTGCTGGCCGGGGAACTATCCCTGATGTCGGCCATTGCAGCAGGGCAGCTGGTGAAGAGCCA
CATGAAGTACAATAGATCCAGCAAAGATATGACCAAAATTGCATCGGCTTAAGGTGGTATCCTTAATGATAAAAT
AAACAATGGAGAAGATTGATACCGAGAGAGGGGGGGATGGGGAAGAAAGTGAAGAGAATAGATGCCCATGTGAGA
TTTTTATGCTAGTTTATTAGATGTAATGTGTACAATTGGGTCTATTGTATGAAGACTTGAGGGTAACTCCTATTT
CATTTTCTTCCTCCTATTGGGGACTGTATCTTCAATTTCAGTTCAGTAATGGGTGTAACCTAGAGGAATCTTAAA
AAAAATATTATGTCCCAATTTGTC

>PB.10075.1 Euphorbia maculata HMG-CoA reductase Para.2 mRNA
CAAATTCCGCCGACCCACCCCCACCGCCGCCGACCGCCGCCCCCTCTCTCCCCCGCCCAAGGCCTCCGACGCCCT
CCCGCTCCCTCTCTACCTCACCAACGCCGTCTTCTTCACCCTCTTCTTCTCCGTCGCCTACTACCTCCTCCACCG
GTGGCGCGACAAGATCCGCAGCTCCACCCCTCTCCACGTCGTCACGCTCTCCGAAATCGCCGCCATTGTCTCCCT
CATTGCGTCCTTCATCTACCTGCTTGGATTCTTCGGGATCGATTTTGTGCAATCCTTCATCGCACGCGCTAATCA
CGACACGTGGGACCTTGACGATGCAGATCGCAGTTTCCTCATCGATGGAGATCACCGCCTTGTCACTTGCCCTCC
CCCAAAGATTGCTTCAATTTCTCCCGTTCCTTCGCCGAAAATGGAACCGATTATTTCGCCTCTAGCCTCTGAGGA
GGACGAGGAGATCGTGAAATCTGTTGTTGACGGAACGATTCCGTCGTATTCGCTGGAATCGAAGCTCGGGGATTG
CAAACGGGCGGCTACAATTCGGCGCGAGGCTTTGCAGAGGTCGATGGGGAGGTCGCTCGAGGGTTTGCCGGTTGA
TGGATTTGATTATGAGTCGATTTTAGGTCAGTGCTGTGAAATGCCGGTGGGATATGTGCAGATTCCGGTGGGAAT
TGCGGGGCCTTTGCTGCTTGATGGAAGAGAGTACTCTGTTCCTATGGCGACTACGGAGGGGTGTTTGGTTGCTAG
TACTAATAGAGGCTGTAAGGCGATTCATTTGTCCGGTGGCGCTAGTAGTGTGTTGTTGAAGGACGGCATGACTCG
AGCTCCGGTGGTTCGGTTTGGGTCGGCGAGGAGGGCGGCGGAATTGAAGTTTTTCTTGGAGAATCCTGACAATTT
CGATACCTTGTCCATCGTCTTTAATAGGTCCAGTAGATTTGCGAAGCTTCAAGGCATACAGTGCTCTATTGCTGG
CAAAAATCTTTACATTAGATTCAGCTGCAGCACTGGTGATGCAATGGGGATGAACATGGTTTCCAAAGGGGTCCA
AAATGTTCTTGAGTTCCTTCAAAGTGATTTCCCTGACATGGATGTTATTGGCATCTCTGGGAATTTCTGTTCGGA
TAAGAAGCCAGCAGCTGTAAACTGGATTGAAGGGCGAGGCAAGTCGGTTGTCTGTGAGGCAATTATAAAGGAAGG
CGTGGTGAAGAAGGTGTTGAAGACCGATGTTGCTTCACTAGTAGAGCTGAACATGCTCAAAAATCTTGCTGGCTC
AGCAGTTGCAGGGGCTCTCGGTGGATTCAATGCACACGCAGGCAACATAGTCTCTGCAATTTTCATTGCCACTGG
CCAAGATCCAGCCCAGAATGTCGAGAGCTCCCACTGCATCACCATGATGGAAGCTGTCAATGACGGCAAGGATCT
CCACATCTCAGTAACCATGCCTTCCATTGAGGTGGGGACAGTTGGAGGGGGAACTCAACTCGCATCGCAGTCAGC
TTGCCTGAATCTGCTAGGGGTGAAGGGGGCAAGCAAGGAAACACCGGGGGCAAACTCGAGGCTCCTGGCGAGCAT
AGTAGCTGGCTCGGTCTTGGCAGGGGAACTGTCCCTGATGTCGGCCATAGCAGCGGGGCAGCTAGTGAAGAGCCA
CATGAAGTACAACAGGTCCAGCAGAGATGTATCCAAAATGGCATCCTCTTAAAAGGGTGGCCCCTTGAGAAAGTG
ATACAGAGAGGAAGTGGGGAAGAAAACAAAGACAAGCAGGTGCACCCATGTGAGATTATTGCTTGACCTTTTTAG
ATTTCATAGCTGTAAAGTTGTAATGGGCTTGGTTTGCTAACTCCATATTGTTGTTCTTCCCCCATTTGTGGACTG
TGCCATCAAGTTCAGTTCTGAGCACAAAATGTCCTGTGATCACATGTAAAAAAAGTCCCCTGACTGTTGAGTGTT
TTTGTGTCCCCATTTGTTTAATGGTTAATGTTCAATTGAATTGCTTC

>PB.10076.1 Euphorbia maculata HMG-CoA reductase Para.3 mRNA
ATTTCCCCCTTTTGGCATCTTCCCCTCTCCTTCTCCGCCATGGATCCCCCCCGCCCCGCCGCCAAACTCCGGCGA
CCCCTCTCGCCGCCGCCCAAGGCCTCCGACGCCCTCCCGCTCCCCCTCTACCTCACCAACGCCGTCTTCTTCACC
CTCTTCTTCTCCGTCGCCTACTACCTCCTCCACCGCTGGCGCGACAAGATCCGCAACTCCACCCCCCTCCACGTC
GTCACGCTCTCCGAAATCGCCGCCATTGTCTCCCTTATTGCGTCCTTCATCTACCTGCTTGGATTCTTCGGGATC
GATTTTGTGCAGTCGTTCATCGCACGCGCTAATCACGACACGTGGGACCTTGACGATGCGGATCGCAATTTCCTC
ATCGACGGAGATCACCGCCTCGTCACTTGCCCTCCCCCGAAGATTGCTTCAATTTCTCCCGTTCCTCCGCCCAAA

ATGGAGCCGATTATTTCGCCTCTTGCCTCCGAGGAGGACGAGGAAATCGTCAAATCTGTTGTCAATGGAACGATT
CCGTCGTATTCCCTTGAATCGAAGCTCGGGGATTGTAAACGTGCGGCTACCATTCGGCGCGAGGCTTTGCAGAGA
ACGATGGGGAGGTCGCTCGAGGGTTTACCGGTTGATGGATTTGACTATGAGTCGATTTTAGGTCAGTGCTGCGAA
ATGCCTGTGGGATATGTGCAGATTCCGGTGGGAATTGCGGGGCCTCTGTTGCTTAATGGAAGAGAGTACTCTGTT
CCTATGGCGACCACTGAGGGCTGTTTGGTTGCGAGTACTAATAGAGGTTGTAAGGCGATTCATTTGTCCGGTGGA
GCTAGCAGTGTTTTGTTGAAGGATGGGATGACTCGAGCACCGGTGGTTCGGTTTGGATCGGCGAGGAGGGCGGCG
GAATTGAAGTTTTTCTTGGAGAATCCTGACAATTTCGATACCTTGTCCGTCGTCTTTAATAGGTCCAGTAGATTT
GCGAAGCTTCAAAGCATACAGTGCTCTGTTGCTGGCAAAAATCTTTATATTAGATTCAGCTGCAGCACTGGTGAT
GCCATGGGAATGAACATGGTTTCCAAAGGGGTCCAAAATGTTCTTGAGTTCCTTCAAAGTGATTTCCCTGACATG
GATGTTATTGGCATTTCTGGGAATTTCTGTTCTGATAAGAAGCCAGCTGCAGTAAATTGGATTGAAGGGCGAGGC
AAGTCGGTTGTCTGTGAGGCAATAATCAAGGAAGATGTGGTGAAGAAGGTGTTGAAAACTGATGTTGCTTCACTT
GTAGAGCTGAACATGCTCAAGAATCTTGCTGGCTCAGCCGTTGCAGGTGCTCTAGGTGGATTCAATGCACACGCA
GGCAACATAGTCTCTGCAATTTTCATAGCCACAGGCCAAGATCCAGCCCAGAATGTGGAGAGCTCTCACTGCATC
ACCATGATGGAAGCTGTCAATGATGGCAAGGATCTCCACATCTCTGTAACCATGCCTTCCATTGAGGTGGGGACA
GTTGGAGGGGGAACTCAACTTGCATCTCAGTCAGCTTGCCTGAATTTGCTAGGCGTGAAGGGGGCGAGCAAGGAA
ACACCAGGGGCAAACTCGAGGCTCCTGGCAAGCATAGTAGCTGGCTCGGTCTTGGCAGGGGAACTGTCCCTGATG
TCGGCCATAGCAGCAGGGCAGCTAGTGAGGAGCCACATGAAGTACAACAGGTCCAGCAGAGATGTATCCAAAATT
GCATCCTCTTAAATGGGTGGCCCCTTCACAAAGTGGAACCAAATGATACAGAGAAGAAAAGGGGAAGAAAACAAA
GACAAGCAGGTGCACCCATGTGAGATTAGTGCTTGTCTGTCCTTTTTAGATTGGATAGCTGTAAAGTTGTAATGG
GTTTGGTTTGCTAACTACATATTGATGTTCTTCCCCCATCTGTGGACTGTCTCATCGAGTTCACATGTAAAAAAA
AAAAATCCCCTGACTGTTGAGTGTTTTTGTGTCCCGATTTGTTGTTTATTGGTTAATGTTCAATTGAATTGCCTC
CCTTTGATCTTCATGTGTATTGTTGTACAATTTCAGCTTGTAGTTGATTTCATCTTTCAATATG

>PB.10077.1 Euphorbia maculata HMG-CoA reductase Para.4 mRNA
AATCAAACACTTCCTGAGCTTAATCAAACCAATAAAAGTTCCCATTTTTTCCTTTTCCCCCGCTCTCTCTCCATC
CTCCGCCCCCCAGAATGGAGGCCCGCCGGCGACCCATTTCAGAAAAACCAGTTAATTCCTTGAAAGTAAAATCGG
TGGAGGATGAGAGCACACAAAAAGCCTCTTCTGGTGAATTACCTCTTCTTCTCTACCTAACAAATGCTCTTTGCT
TCACGGTGTTCTTTTGGGTTGTTCATTTTCTTCTCAGCCGTTGGCGTGAAAAGATTCGAACTTCAACTCCTCTCC
ATGTTGTTTCTTTCTCCGAAATTTGTGCCATTTTTGGGTTTTTCGCTTCCTTCGTTTACCTCCTTGGTTTCTTCG
GTTTTGACTTTGTCCAGTCCCTGATTTTGCGGCCTCCGGCTGATGTTTGGACCTCTGATGATGAAGAAGAATTAG
CCAACAAGGATGATTCTCGTAAATTGCCATGTGGTCAAGGTTTAGATTGCTCGATTTCTTCAATTGAGGCTCCTT
TGACTAAACCCCACAAGGTGTTCGATGAAAGTCCTCAACCAGAAACCAACTTTTTCACTGATTCAGATGAGGAGA
TAATCAAATCTGTAGTGGATGGGAAAACACCGTCTTACTCTCTGGAGTCAAAATTGGGGGATTGTAAGCGAGCTG
CTGCAGTTAGGCGCGAGGCTTTACAAAGAATCACAGGCAAATCCCTTGCGGGATTGCCATTAGAGGGTTTTGATT
ATGAGTCAATTTTGGGGCAGTGCTGTGAGATGCCAGTTGGGTATGTTCAAATTCCTGTTGGAATTGCTGGCCCGT
TGTTGCTCAATGGGAAGGAGTACAGTGTTCCAATGGCTACGACGGAAGGGTGTTTGGTGGCTAGTACTAACAGAG
GTTGCAAGGCGATTCACTTATCTGGTGGAGCCACTAGTGTGTTGTTAAAAGACGGGATGACGAGAGCGCCTGTTG
TTAGATTTGGGACGGCTAGAAGGGCTGCCCAGTTGAAGTTTTACTTGGAGGATCCTGCTAATTTTGGGGCTATTT
CTTCTGCTTTTAATAGATCAAGCAGATTTGGTAGGTTACAGAGTGTTAAATGTGCTATTGCTGGCAAAAATCTCT
ATTTGAGATTCACTTGTAGCACTGGTGATGCTATGGGTATGAATATGGTGTCTAAAGGTGTGCAGAATGTGCTGG
AGTTTCTCCAACCTGATTTTCCTGATATGGACGTTTTGGGCATCTCTGGGAACTATTGTTCTGATAAGAAGCCTG
CAGCTGTGAACTGGATTGAAGGAAGAGGAAAATCAGTAGTATGTGAGGCGATTATAAAGGGGGACATTGTGAAAA
AGGTTCTAAAGACAAATGTTGAGGCCTTGGTAGAGCTCAACATGCTCAAGAATCTTACTGGTTCAGCCATGGCTG
GAGCTTTAGGTGGCTTCAACGCTCACGCTAGTAACATTGTCTCAGCAATTTACATAGCAACCGGTCAAGACCCTG
CACAAAATGTGGAGAGTTCTCACTGTATAACTATGATGGAAGCTGTTAATGATGGTCAAGATCTTCATGTCTCTG
TTACCATGCCTTCTATTGAGGTTGGAACTGTTGGAGGCGGAACACAACTAGCATCTCAATCAGCATGCTTAAACT
TGCTTGGGGTGAAAGGCGCAAGCAAAGAAACGCCCGGAGCTAACTCGAGGCAATTGGCAGCCATTGTTGCAGGTT
CTGTTCTTGCTGGAGAGCTTTCTCTCATGTCTGCAATTGCTTCAAATCATCTTGTTAACAGTCACATGAAATATA
ACAGAGCACCATCTAAGAACTGATTTTGTAATTACTTCCCACACCCGAAACATAGTTTTTCATTACTTCAACTGC
CAATGACATCACTGTTGTGTGTAATTTGTTGTTTTAGTTTCTATGTTTGTATTTATATTCATCCAAATCCTTGTA
TAAAATATCTGCATATTAGGTGAAATCATTCAAATGTAGTTGAATATCCACGCTACAATTTCCTGAAAATAATGT
AGTTATTTTTTCCTCCATTTGTTCTTTTGTTTTGAAAGATTGTATCGACAATGGTGCTTAAGGGAAGTTAAGTTT
TCACT

>PB.10078.1 Euphorbia maculata HMG-CoA reductase Para.5 mRNA
CTCCCATCAAACACAACACATTGCCACTTTGATCACACACAGCAAAGCTTCAATCTTTCCTCTCCCGCCCCCCTC

CGCCGCCGGAAATGGACCCCCGCCGGCGACCCATCTCGGAAAAACCGCTCAATTCGTTGAAGCCCAAACTGCCGG
AGGATCAAAAGCCCTCTTCCGACGCTTTACCTCTTCTTCTCTACCTCACCAACGCTCTCTTCTTCTCTGTGTTCT
TCTGGGTCGTCTATTTCCTCCTCAGCCGCTGGCGCGAGAAAATCCGCACTTCGACTCCTCTCCACGTCGTCACTC
TCTCCGAGATTTGCGCGATTGTTGGGTTTTTCGCTTCGTTTGTTTACCTTCTTGGTTTCTTCGGTATTGACTTTG
TCCAGTCCCTGATTTTGCGCCCTCCGACTGATTTCTGGTCCTCCGATGATGAGGACGAATTAGTCAAAACTGAGG
ATCGCAAATTGCCGTGTGATCAGGGTTTAGATTGCTCCATTTCGCCCCCTCTTCTGGCTCCTTCGGTTAAGCCCC
ACAAGGTGTTCGATGAAATTCCCCAACCAGAATCCAGTTTTTTTACTGATTCTGATGAGGAGGTAGTCAAATCTG
TGGTGGAGGGGAAAACCCCGTCTTACTCTCTAGAGGCGAAATTGGGGGATTGTGAGCGTGCGGCTGCAATTAGGC
GTGAGGCGTTACAGAGAATAACGGGCAAGTCCCTAGCTGGATTGCCACTAGAGGGGTTTGATTACGAGTCAATTT
TGGGCCAGTGCTGTGAGATGCCAGTTGGGTTTGTTCAGATCCCAGTTGGGATTGCGGGCCCTCTGTTGCTTAACG
GGAAGGAATTCAGTGTTCCAATGGCTACAACTGAAGGGTGTCTGGTGGCTAGCACTAACAGGGGTTGCAAGGCAA
TCCACTTGTCTGGTGGAGCCACTAGTGTGCTGTTAAAAGATGGGATGACTAGAGCGCCTGTAGTTAGATTCGGGA
CAGCGAAAAGAGCTGCTCAGTTGAAGTTTTTCCTTGAGGATCCTGCTAATTTTGAGGCTATTTCTTCTGCTTTTA
ATAAATCAAGTAGATTTGGGAGATTGCAGAGTATTAAGTGTGCTATGGCCGGCAAAAATCTTTACCTGAGGTTCA
CTTGTAGCACTGGTGATGCCATGGGAATGAATATGGTGTCTAAAGGTGTGCAAAATGTGCTGGAATTCCTTCAAG
CTGATTTTCCTGACATGGATGTTCTGGGCATTTCTGGCAACTATTGTTCTGATAAGAAGCCTGCTGCTGTGAATT
GGATAGAAGGAAGAGGGAAATCAGTAGTATGCGAGGCAATTATAAAGGGCGATATTGTGAAGAAGGTTCTAAAGA
CAAATGTTGAGGCCTTGGTAGAGCTCAACATGCTCAAGAATCTTACTGGTTCAGCCATGGCTGGAGCCTTAGGCG
GCTTTAATGCTCATGCTAGCAACATTGTCACAGCTATTTACATAGCAACCGGTCAAGACCCGGCACAAAATGTAG
AGAGCTCTCACTGTATTACTATGATGGAAGCTGTTAATGATGGCCAAGATCTTCATGTCTCCGTCACTATGCCTT
CTATCGAGGTTGGAACCGTTGGAGGCGGAACACAACTGGCGTCTCAATCAGCTTGCTTAAACTTGCTTGGGGTGA
AAGGGGCAAGCAAAGAGACACCAGGGGCAAACTCGAGGCAGTTGGCAGCCATTGTTGCAGGTTCTGTTCTTGCTG
GCGAGCTCTCTCTCATGTCTGCAATTGCTTCAAATCAACTGGTTAACAGTCACATGAAGTACAACAGAGCACCAT
CCAAGAATTGATCTCTTAGTTAAATTAATTACACAAATGTTACTGTTTTTGTATAATTTGTTCTTATTTGTGTTT
GTACTCAATCAAATCCTTGTAAGAAATATCTGCCAATTGTGTAAATTTATTCGAAAAGTCTATTATCCTTTTCAT
TTTGTGATTTGAGCAGTAAAAGACAATATAACTCCTGTCGTTGATAGTCTTGGTCC

>PB.13628.1 Euphorbia maculata MVA kinase mRNA
AATAGATTTCATCTCAAACTATTTCAATTTCCATCTATCATAGTATTGACATTTATCGAGAAATAGGAAGGTTCC
TTTGAGAAGGGGAATTTGGGGACTTTTGAAACATGGAAGTGAAAGCAAGAGCTCCAGGAAATATCATTCTCTCTG
GTGAACATGCAGGTGGTTCATGGTTCCACCGCAGTTGCTGCGTCCATTGATCTCTACACTTGTGTTACCCTTTCT
TTTCCCACTTCTGAGGATGCTAATTCACTAGTACTTCAGCTTAAGGACGTGGGACTAGAATTTTCATGGCCTATT
GATAGAATAAAAGAAGCATTCTCTAGCATTGATGTCCCATCCTCTTCGAAACCTACCTCTTGCTCTTCGGAATCA
ATTAAGTCAATTTCAGTTTTGATTGACGAACAAAATATCCCAGAAGCAAAAATTTCACTTGCTTCCGGGGTTTTG
GCATTTCTGTGGTTATATACTTCTATCCAAGGATTTAAACCTGCCAATGTAGTTGTCAATTCAGATCTTCCTCTG
GGTTCGGGCCTCGGCTCATCAGCTGCATTTTGTGTTGCTCTTTCTGCTGCTCTTCTTGCTGTATCAGACTGTCTA
TATGTCGATAAGCAGCATGAAGGATGGATGAGATATGGAGATAGTGAACTTGAATTGTTAAATAAATGGGCATTT
GAAGGCGAAAAGTTGATTCATGGAAAGCCATCTGGAATTGACAACACTGTTAGCACTTACGGCAACATGATTAAG
TTCAGGTCTGGTAATCTAACTCGAATGAAGTCCAACATGCCGCTCAAAATGCTTATAACTAACACAAAAGTGGGG
AGGAACACAAAAGCACTACTTGCTGGTGTTTCGGAAAGAACCTTTCGGCATCCTAATGCCATGAGTTTTGTCTTT
AACGCGGTCGATTCTATCAGCAATGAGCTGGCTAATATCATCCAGGAGCCGGCCCCTGATGAGTTAACCGTTACA
GAGAAGGAAGAGAAGCTAGAAGAGCTAATGGAAATGAACCAAGGCTTGCTCCAATGCATGGGGGTTAGCCATGCT
TCTATTGAAACTGTTCTCCGCACAACATTGAAGTACAAGTTGTCTTCCAAGTTGACTGGAGCTGGCGGTGGAGGC
TGTGTGCTCACCTTGTTACCAACCCTGCTATCAGGAACAGTTGTTGACAAGGTTGTTGCAGAGCTCGAGTCGAAT
GGATTCCAATGTATGATTGCTGGAATTGGTGGAAATGGAGCTGAGATCTGCTTTGAAAGTTCATCGTCGTCTTGA
TCATGCAAAAGCTGCAATGAAGTTAAAAGAGTATGATCTTATTTCTAAAGTCAAATAATTGCAAAATAAGTACTT
TCTTTTTTATGCTAAAGCTTGATTTGTATTTTCCTATTCAATCTGTACATATATTGTGCCGAGTTGTGCATGTTT
TTGGAATTTTGATTGTTCTTTTTCAGTAATTGAGTCAAACATTTTACATAATGTGTCAATGCAATCTGGCTTTGT
TTAGTCTCGTCTAATTACC

>PB.10223.1 Euphorbia maculata MVAP kinase Para.1 mRNA
GCGAGGAAGCATGATGAGCTATCGCACAGATCGAAGTCCAACCATGACTTTGACGTTCTTGTTTTCAACGTCAAA
ACTTCATTCTCTCGACGGCTCCACTTCCTCATCGCCGATTTGCACTCTCCGATTTCGTAGCTAGCCTTGATCCCT
CTCTACGATTTCCAAGCTCGGAGCTCTCTCTTCTCTATCTGCTTCTTTTTAAGCTTCTTACTCCTACTCTCAATT
ATTCAGGATTCAAGTTCGCAGTTGGTTCAGCTTTCGTTGAGTTGTTTATTTTCTCCAGATAAATTATTCTGCTCG
TTGTCTACATCTTCTTGAAGATCAGGTTGCAATTCATAGAGAAGAAGACAATGGCAGTAGTTGCTTCTGCTCCTG

GTAAGGTGTTGATGACTGGGGGTTACCTCGTACTGGAAAGACCCAATGCTGGGATAGTTCTTAGTACAAATGCTC
GATTCTATGCGATTGTGAAGCCTTTATATGATGCAATCAAGCCCGATAGCTGGGCATGGGCATGGACTGATGTGA
AATTAACTTCTCCCCAACTTTCTAGAGAAAGCACTGTACAAATTATCAATGAAGAATTCGACTTTACAATGTGTC
TCTCCAAACCAATCACGGAACCCTTTTGTGGAGCAATCAGTTCAACATGCTGTAGCAGCAGCGCATGCAACTTTG
GGCGAAGACAAGAAGGATGCACTGAACAAGCTACTCTTGCAAGGTCTTGATATCACAATTTTGGGTTCCAACGAT
TTTTATTCATATCGAAATCAGATTGAAGCACGCGGGCTCCCTTTGACACCTGAGGCTTTAGCTTCACTTCCTCCA
TTCACCTCAATCACCTACAACGCAGAAGAAGAAAATGGAGGAAACAGTAAACCTGAGGTGGCTAAAACTGGATTG
GGTTCTTCAGCAGCAATGACCACTGCTGTTGTTGCCGCTTTGCTTCATTATCTCGGAGTGGTTGATCTTTCATCT
ATTAGTAAGGATGAGGAATCTAGTGATCTTGATGTGGTGCATATAATAGCTCAAACTGCTCACTGCATTGCACAA
GGGAAAGTTGGCAGTGGTTTTGATGTCAGCTCTGCAGTTTATGGAAGTCATCGTTATGTTCGGTTTTCACCGGAA
GTGTTATCCCCAGCTCAGGATATTTCCAAATGGGTTTCGCTACAAGATGTCACTATTAGCATTTTAAAAGCAAAG
TGGGACCATGAAAGGACTAAGTTTTCGTTGCCACCGTTATTGAACCTGTTGCTTGGAGAACCAGGAACTGGAGGA
TCATCTACACCGTCAATGGTAGGTGCAGTTAATAAGTGGAAGAAGCTTGAACCTCAGGAATCCCAGGAAACATGG
AGAAAGCTGTCAGAGGCAAATTCAGCACTTGAAACTCATTTCAATGTTTTGAGCAAGCTTGCAGAAGAACACGGG
GATGCATACAAGTCCATGATAGACTGCTGCAGCATGCAGAAGGCACAACGGTGGATGGAACAGGCCACTGAACCA
GCCCAAGAAGCCGTTGTGAAAGCTCTACTAGGTGCAAGAAATTCAATGCTTGATATCAGAAATCTCATGCGCCGG
ATGGGCAAGGCTGCAGGTGTTCCGATTGAGCCAGAATCACAGACCCAACTTCTGGATAGGACAATGGATATGGAG
GGAGTATTGTTGGCTGGAGTTCCTGGAGCCGGTGGGTTTGATGCGATCTTTGCAATTACAATGGGAGAGAGTAGT
AGCAGAGTGGCAAAAGTGTGGAGCTCGCTTGATGTTCTAGGCATGCTGGTTAGAGAAGATGCTCGGGGCATTGCT
TTAGAAAGCAGTGATCCCAGAATCAACGATGTTACATCCGCAGTTTCTGCAGTTCAGATTCATTGAGGTGTGGTT
TTCTTTTGTATTTTGACGAGATCGGCCCACTGTTATGGTGTGGGAAATACATGTTATAAGGGAAACTAGACTAGT
TTTTGGGTTAAAATAATGAAAGGGGAAAACAAAAAATGAAAGAAGAGGATAATTACTGATAGCTTGTATTTGTGA
TTCCATTTGTAAGGATGTGATTAAATTTCTCTGAATAATGGCCTAATAAAGAAACTATTTGGTTGTTTTTCGGGC
C

>PB.10224.1 Euphorbia maculata MVAP kinase Para.2_Iso.1 mRNA
AAACATGACTTTGACGTTCTTGTTTTCAACGTCAAAACTTCTTTCTCTCGACGGCTCCACTTCCTCATCGCCGAT
TAGTACTCTCCGACTTTGTCGTTGCACTTGATCCTTCTCTTCGAATTCCAAGCTCTGAGCTCTCTCTACTCTATT
GGCTTCATTTTGAGCTTTTTTCTCCTGCTCTCATTTATTAAGGATTCAAGTTTTCAATTGGTTCAGCTTTCGTTG
AGGAGCTTCATTCGTCCGTTTAATCTCTTTTGAGTTATTATATTATATCCAGATAAATTCTTCTGCGCGTTGACT
GCATCTTCTCGGAAAGACAGGTTGCAATCGACAGAGAAGAAGGCAATGGCAGTAGTTGCTTCTGCTCCTGGTAAG
GTGTTGATGAGTGGGGGTTACCTCATACTGGAAAGACCCAATGCTGGGGTAGTACTCAGCACAAATGCTCGATTC
TATGCGATTGTGAAGCCTTTATATGATGCAATCAAGCCCGGTAGCTGGGCATGGGCATGGACTGATGTAAAATTA
ACTTCTCCCCAACTTTCTAGAGAAAGCACGTACAAATTATCAATGAAGAATTCAAGTCTACAATGTGTCTCTCCA
AACCAATCGCGGAACCCTTTTGTGGAACAATCAGTACAATATGCTGTAGCAGCAGCGCATGCAACCCTGGGCGAA
GATAAGAAGGATGCACTGAACAAGCTACTCTTGCAAGGTCTTGATATCACAATTTTGGGTTCCAATGATTTCTAT
TCATATCGAAATCAGATTGAAGCACGCGGGCTCCCTTTGACACCAGAGGCTTTAGCTTCACTTCCTCCATTCACC
TCAATCACCTACAATGCAGAAGAAGCAAATGGGGGAAACTGTAAACCTGAGGTGGCTAAAACTGGATTGGGCTCT
TCAGCAGCAATGACCACTGCTGTTGTGGCCGCTTTGCTTCATTATCTCGGAGTGGTTGATCTTTCATCTCTTAGT
AAAGATAACGAATCTAGCGATCTTGATGTGGTGCATATAATAGCTCAAACTGCTCACTGCATTGCACAAGGGAAA
GTTGGCAGTGGTTTTGATGTCAGTTCAGCAGTTTATGGAAGTCATCTTTATATTCGCTTTTCGCCAGAAGTGTTA
TCCCCAGCTCAGGATATTTCCAAAGGGGTTTCACTACAGGATGTCACTGTTAGCATTTTAAACGCAAAGTGGGAC
CATGAAAGGACTAAGTTTTCCTTGCCACCTTTATTGAACCTGTTGCTTGGAGAACCAGGAAGTGGAGGATCATCT
ACGCCATCAATGGTAGGTGCAGTGAATAAGTGGAAGAAGCTTGAACCTCGGGAGTCCCAGGAAACATGGAGAAAG
CTGTCAGAGGCAAATTTAGCACTTGAAACTCATTTCAATATTTTGAGCAAGCTTGCAGAAGAACATTGGGATGCA
TATAAGTCCATGATAGACTGCTGCAGCATGCTGAAGGCACAACAGTGGATGGAACAGGCTACTAAACCAGCCCAA
GAAGCCATCCTGAAAGCTCTATTAGGCGCAAGAAATGCAATGCTTGATATCAGAAATCTCATGCGCCGGATGGGT
GAGGCTGCAGGTGTACCGATTGAGCCAGAATCACAGACCAAACTTCTAGATAGGACAATGGAAATGGAGGGAGTT
TTGTTGGCTGGAGTTCCAGGAGCCGGTGGGTTTGATGCAGTCTTTGCAATTACAATGGGAGAGAGTAGTAGCAAA
GTGGCAAAAGCATGGAGCTCACTTAATGTTCTGGCCATGTTGGTTAGAGAAGATGCTCGGGGTGTTGCTTTAGAA
AGCGGTGATCCCAGAATCAAGGATGTTACATCAGCAGTTTCTGCAGTTCAGATTCATTGAGGTATGGTTTTCTTT
TGTTTTTTTATGAGATTGGCCCACTGGTATAATGTGGGAAATACATGTTTTAAGGGAAACTGAACTAGTTTTTCG
GTTGAAATAATCGAAAGGGAGAACAAAAAGAAAAAACAATATTAATACTGATAGCTTCTATATGTGATTCCATTT
GTAAGGATGTGATCAAATTTCTCTGAATAATGGCCTATATCATGAATAAAGTACTTGGTTGTTTTCGGGCTG

>PB.10224.2 Euphorbia maculata MVAPP Para.2_Iso.2 mRNA

ATGACTTTGACGTTCTTGTTTTCAACGTCAAAACTTCTTTCTCTCGACGGCTCCACTTCCTCATCGCCGATTAGT
ACTCTCCGACTTTGTCGTTGCACTTGATCCTTCTCTTCGAATTCCAAGCTCTGAGCTCTCTCTACTCTATTGGCT
TCATTTTGAGCTTTTTTCTCCTGCTCTCATTTATTAAGGATTCAAGTTTTCAATTGGTTCAGCTTTCGTTGAGAT
AAATTCTTCTGCGCGTTGACTGCATCTTCTCGGAAAGACAGGTTGCAATCGACAGAGAAGAAGGCAATGGCAGTA
GTTGCTTCTGCTCCTGGTAAGGTGTTGATGAGTGGGGGTTACCTCATACTGGAAAGACCCAATGCTGGGGTAGTA
CTCAGCACAAATGCTCGATTCTATGCGATTGTGAAGCCTTTATATGATGCAATCAAGCCCGGTAGCTGGGCATGG
GCATGGACTGATGTAAAATTAACTTCTCCCCAACTTTCTAGAGAAAGCACGTACAAATTATCAATGAAGAATTCA
AGTCTACAATGTGTCTCTCCAAACCAATCGCGGAACCCTTTTGTGGAACAATCAGTACAATATGCTGTAGCAGCA
GCGCATGCAACCCTGGGCGAAGATAAGAAGGATGCACTGAACAAGCTACTCTTGCAAGGTCTTGATATCACAATT
TTGGGTTCCAATGATTTCTATTCATATCGAAATCAGATTGAAGCACGCGGGCTCCCTTTGACACCAGAGGCTTTA
GCTTCACTTCCTCCATTCACCTCAATCACCTACAATGCAGAAGAAGCAAATGGGGGAAACTGTAAACCTGAGGTG
GCTAAAACTGGATTGGGCTCTTCAGCAGCAATGACCACTGCTGTTGTGGCCGCTTTGCTTCATTATCTCGGAGTG
GTTGATCTTTCATCTCTTAGTAAAGATAACGAATCTAGCGATCTTGATGTGGTGCATATAATAGCTCAAACTGCT
CACTGCATTGCACAAGGGAAAGTTGGCAGTGGTTTTGATGTCAGTTCAGCAGTTTATGGAAGTCATCTTTATATT
CGCTTTTCGCCAGAAGTGTTATCCCCAGCTCAGGATATTTCCAAAGGGGTTTCACTACAGGATGTCACTGTTAGC
ATTTTAAACGCAAAGTGGGACCATGAAAGGACTAAGTTTTCCTTGCCACCTTTATTGAACCTGTTGCTTGGAGAA
CCAGGAAGTGGAGGATCATCTACGCCATCAATGGTAGGTGCAGTGAATAAGTGGAAGAAGCTTGAACCTCGGGAG
TCCCAGGAAACATGGAGAAAGCTGTCAGAGGCAAATTTAGCACTTGAAACTCATTTCAATATTTTGAGCAAGCTT
GCAGAAGAACATTGGGATGCATATAAGTCCATGATAGACTGCTGCAGCATGCTGAAGGCACAACAGTGGATGGAA
CAGGCTACTAAACCAGCCCAAGAAGCCATCCTGAAAGCTCTATTAGGCGCAAGAAATGCAATGCTTGATATCAGA
AATCTCATGCGCCGGATGGGTGAGGCTGCAGGTGTACCGATTGAGCCAGAATCACAGACCAAACTTCTAGATAGG
ACAATGGAAATGGAGGGAGTTTTGTTGGCTGGAGTTCCAGGAGCCGGTGGGTTTGATGCAGTCTTTGCAATTACA
ATGGGAGAGAGTAGTAGCAAAGTGGCAAAAGCATGGAGCTCACTTAATGTTCTGGCCATGTTGGTTAGAGAAGAT
GCTCGGGGTGTTGCTTTAGAAAGCGGTGATCCCAGAATCAAGGATGTTACATCAGCAGTTTCTGCAGTTCAGATT
CATTGAGGTATGGTTTTCTTTTGTTTTTTTATGAGATTGGCCCACTGGTATAATGTGGGAAATACATGTTTTAAG
GGAAACTGAACTAGTTTTTCGGTTGAAATAATCGAAAGGGAGAACAAAAAGAAAAAACAATATTAATACTGATAG
CTTCTATATGTGATTCCATTTGTAAGGATGTGATCAAATTTCTCTGAATAATGGCCTATATCATGAATAAAGTAC
TTGGTTGTTTTCGGGCTAT

>PB.13376.1 Euphorbia maculata MVAPP decarboxylase Para.1_Iso.1 mRNA
CTCTTCAATTGAGTTTATAAAATTCCCCCAATTTCCCTCCTCTTCTCCCTACTCCTTCCTCCTGTGATCTTCCTT
CCTCTTTGCTCTACAATGGCGGAGTCTTGGGTGAGGATGGTCACTGCGCAAACTCCGACCAACATTGCAGTCATC
AAGTACTGGGGGAAACGGGACGAGTCCTTGATTTTGCCTGTCAATGATAGCATTAGTGTCACTCTAGATCCTTCT
CATCTTTGCACCACCACCACTGCTGCTGTCAGCCCTTCTTTTGATCGGGATCGCATGTGGCTTAACGGAAAGGAG
ATTTCCCTTTCTGGGAATAGATTTCAGAGTTGTTTGAGGGAAATTAGAGCCCGAGCTTGTGAAGTTGAGGATGAA
GAGAAGGGAATTAAAATTTCAAAGAAGGATTGGGACAAGTGGCATGTACATATAGCTTCGTATAACAATTTCCCT
ACTGCTGCTGGACTGGCTTCTTCAGCTGCTGGCTTTGCTTGCTTTGCCTTTTCCCTTGCAAAGCTAATGAATGCT
AAAGAGGATAACAGTGAGCTTTCTGCTATTGCAAGGCAAGGTTCAGGAAGTGCTTGTCGCAGTTTGTTTGGTGGA
TTTGTGAAGTGGAACATGGGACAAGCTGACAATGGAAGCGACAGCCTTGCTGTTCAAGTGGTAGATGAGAAGCAC
TGGGATGAACTTGTTATTATTATCGCTGTGGTAAGTTCAAGGCAGAAAGAAACTAGCAGCACGTCAGGAATGCGT
GATACTGTGGAGACTAGCTTACTTTTACAACATAGAGCCAAGGAAATTGTGCCAAAACGCATTGTAGAAATGGAA
GACGCCATAAAAAATCGAGATTTTGCATCTTTCGCACGTTTAACCTGTGCTGATAGCAACCAGTTCCATGCTGTC
TGCTTAGATACACAACCTCCGATTTTCTACATGAATGATACTTCGCACAGGATAATCAATTGTGTCGAGAAATGG
AATCACTTTGAAGGAAACCCCCAGGCAGCATATACATTTGATGCTGGGCCGAATGCAGTACTCATTGCACGTAAT
AGGAAGGCTGCTGTCCACTTGATGCAGAAGCTTCTTTTCTACTTTCCTCCAAACTCCGATACTGATTTAAGCAGT
TACGTGGTTGGTGACAAAACCATATTGAAGGATGCTGGGATCGAAGAGATGAAGGATATAGACTCAATTTCGCCA
CCTCCAGAGTCGAAGGATGCTCAAAGATGCAAAGGAGATGTCAGTTATTTCATATGCACAAGACCCGGTAGAGGT
CCCGTTTTGCTATCTGATGAAAGCCAAGCACTTCTCAACCCGGAAACTGGGCTTCCTAAGTGAAATGGTGTTATC
TTCTCGGGCCTATATCTTGGCGATGGTTTTTTCTAGTGAATTCTTTATTGAGTTTGCAATGTTGGTTCTTGTGTC
TCGTGTGTTTTTTTGTAGCTCATTGTGTTTTGGATTATTCAATGTACTCAAGTAAATGAGCTGTCGGTTGAAGCTT
CCGGCAGCTGCATGTTTATTTTATATAATTTATGCAGTATCAGGAATTCTGTAGCTGTGATGCTCATATCAACGA
ATAAACTCCGGGTGTTGTGATTCAAAGGGGTCTTGGTTGGAGTCTGATAGTTTGAAAGCAATGGCCTTTTAATGC
CG

>PB.13376.2 Euphorbia maculata MVAPP decarboxylase Para.1_Iso.2 mRNA
CTCTTCAATTGAGTTTATAAAATTCCCCCAATTTCCCTCCTCTTCTCCCTACTCCTTCCTCCTGTGATCTTCCTT

CCTCTTTGCTCTACAATGGCGGAGTCTTGGGTGAGGATGGTCACTGCGCAAACTCCGACCAACATTGCAGTCATC
AAGTACTGGGGGAAACGGGACGAGTCCTTGATTTTGCCTGTCAATGATAGCATTAGTGTCACTCTAGATCCTTCT
CATCTTTGCACCACCACCACTGCTGCTGTCAGCCCTTCTTTTGATCGGGATCGCATGTGGCTTAACGGAAAGGAG
ATTTCCCTTTCTGGGAATAGATTTCAGAGTTGTTTGAGGGAAATTAGAGCCCGAGCTTGTGAAGTTGAGGATGAA
GAGAAGGGAATTAAAATTTCAAAGAAGGATTGGGACAAGTGGCATGTACATATAGCTTCGTATAACAATTTCCCT
ACTGCTGCTGGACTGGCTTCTTCAGCTGCTGGCTTTGCTTGCTTTGCCTTTTCCCTTGCAAAGCTAATGAATGCT
AAAGAGGATAACAGTGAGCTTTCTGCTATTGCAAGGCAAGGTTCAGGAAGTGCTTGTCGCAGTTTGTTTGGTGGA
TTTGTGAAGTGGAACATGGGACAAGCTGACAATGGAAGCGACAGCCTTGCTGTTCAAGTGGTAGATGAGAAGCAC
TGGGATGAACTTGTTATTATTATCGCTGTGGTAAGTTCAAGGCAGAAAGAAACTAGCAGCACGTCAGGAATGCGT
GATACTGTGGAGACTAGCTTACTTTTACAACATAGAGCCAAGGAAATTGTGCCAAAACGCATTGTAGAAATGGAA
GACGCCATAAAAAATCGAGATTTTGCATCTTTCGCACGTTTAACCTGTGCTGATAGCAACCAGTTCCATGCTGTC
TGCTTAGATACACAACCTCCGATTTTCTACATGAATGATACTTCGCACAGGATAATCAATTGTGTCGAGAAATGG
AATCACTTTGAAGGAAACCCCCAGGCAGCATATACATTTGATGCTGGGCCGAATGCAGTACTCATTGCACGTAAT
AGGAAGGCTGCTGTCCACTTGATGCAGAAGCTTCTTTTCTACTTTCCTCCAAACTCCGATACTGATTTAAGCAGT
TACGTGGTTGGTGACAAAACCATATTGAAGGATGCTGGGATCGAAGAGATGAAGGATATAGACTCAATTTCGCCA
CCTCCAGAGTCGAAGGATGCTCAAAGATGCAAAGGAGATGTCAGTTATTTCATATGCACAAGACCCGGTAGAGGT
CCCGTTTTGCTATCTGATGAAAGCCAAGCACTTCTCAACCCGGAAACTGGGCTTCCTAAGTGAAATGGTGTTATC
TTCTCGGGCCTATATCTTGGCGATGGTTTTTTCTAGTGAATTCTTTATTGAGTTTGCAATGTTGGTTCTTGTGTC
TCGTGTGTTTTTTGTAGCTCATTGTGTTTTGGATTATTCAATGTACTCAAGTAAATGAGCTGTCGGTTGAAGCTT
CCGGCAGCTGCATGTTTATTTC

>PB.13377.1 Euphorbia maculata MVAPP decarboxylase Para.2 mRNA
AATTCCATTGAGTTTATAAACTGCCCCAAATTCCCTCCTCTTCTTCCTACTCCTTCCTCCTCTGATCTCTCTCTT
TGCTCTACAATGGCGGAGACTTGGGTGAGGATGGTCACTGCGCAAACTCCGACTAACATTGCAGTCATCAAGTAT
TGGGGGAAACGGGACGAGTCCTTGATTTTGCCTGTCAATGATAGTATTAGTGTCACTTTAGATCCCTCTCATCTT
TGCACTACCACCACTGCTGCTGTCAGCCCTTCTTTTGATCGCGATCGCATGTGGCTTAACGGAAAGGAAATTTCC
CTCTCTGGAAACAGATTTCAGAGTTGTTTGAGAGAAATTAGAGCGCGAGCTTGTGAAGTTGAGGATAAAGAGAAG
GGAATTACAATTTCAAAGAAGGATTGGGAGAAGTTGCATGTACATATAGCTTCTTATAACAATTTCCCTACTGCT
GCTGGACTGGCTTCTTCAGCTGCTGGTTTTGCTTGCTTTGCCTTTTCCCTTGCAAAGCTAATGAATGCTAAAGAG
GATAACAGTGAGCTTTCTGCTATTGCAAGGCAAGGTTCAGGTAGTGCTTGTCGCAGTTTGTTTGGTGGATTTGTG
AAGTGGAACATGGGACAAGCTGACAATGGAAGCGACAGCCTCGCTGTTCAAGTTGTAGATGAGAAGCACTGGGAT
GAACTTGTTATTATTATCGCTGTGGTAAGTTCAAGGCAGAAAGAAACTAGCAGCACGTCAGGAATGCGTGATACC
GTGGAGACTAGCTTACTTTTACAACATAGAGCTAAGGAAATTGTGCCAAAACGCATTGTAGAAATGGAAGATGCT
ATAAAAAATCGAGATTTTACATCTTTCGCACGTTTAACCTGTGCTGATAGTAACCAGTTCCATGCTGTCTGCTTA
GATACACAACCTCCAATTTTTTACATGAACGATACCTCGCACAGGATAATCAATTGTGTCGAGAAATGGAATCGC
TTTGAAGGAAACCCCCAGGCAGCATATACATTTGATGCTGGGCCCAATGCAGTACTCATTGCACGTAATAGGAAG
GCTGCTATCCAGTTGATGCAGAAGCTTCTTTTCTACTTTCCTCCAAGCTCTGATACTGATTTAAGCAGTTACGTG
GTTGGTGACAAAACCATACTGAAGGATGCTGGGATTGAAGAGATGAAGGATATAGAATCAATTTCGCCACCTCCA
GAGTCAAAGGATGCTCAAAGATGCAAAGGAGAAGTCAGTTATTTCATATGCACGAGACCTGGTAGAGGTCCTGTT
TTGCTATCTGATGAAAGTCAAGCACTTCTCAACCCCGAAACTGGGCTTCCTAAGTGAAATGGCGTTATCTTCTCG
GGCCTATATCTTGATGATGGTTTTTTCTGATGAATTCTTTATTGAGTTTGTAATGTTGGTTCTCGTGTCTGTGTG
CTTTTTGTAGCTTATTGTGTTTTGGATTATTCAATGTACTCAAGTAAATGAGCTGTCGGTTGAAGTTTCCGGTAG
CTGCAAGTTTTTCTTATAATTTATGGAGTATCAGGAAGTCTGTAGCTGTGATGCACTCATCAATGAATAAACTCA
GGTGTTGTGATTC

MEP pathway genes

>PB.5901.1 Euphorbia maculata DOXP synthase Para.1_Iso.1 mRNA
ATAGCCAAACAAAAGAGACAAAAATGGAAGTCACAGAGCAAACAAAAGACGGCCAAAAGCCAACAACGAGATTCT
CAATTCTCAAAGCACCAAATCAATAATCGCAAATGAAAACCGATTCCACCAATCTTCAGAATTATGCTCCCTTTC
TCTCGAAGTTACCCACCATTGATCATTGTCTTGTTGATGAGCTCTCAAATTACCCATTCGATTTGTGCTGAAATT
TGGGGGCTAATCTCTCTGTTTTGCTATGGCTGCTGCTTCAGCTCAGTACACCAATGGAATCGCTGCAAATTCGTG
TAAGAGATTTGGTCCCATTAGGGATTTTTCGGTCTCCGATTTCCCTTGTAAAGCTGGGTTTTCGAGAACTGATTT
CAATCCGAGCTCTTCTTCCATCTCCCATTCCAAGGGTTTTGTGAATCGAATATGTTCGGTGCCTGATCTTGATGA
TATTTTCTGGGAGAAAACTCCTACTCCAATGCTTGATTTAGTTGAAAACCCTATTCATTTGAAGAATCTATCAAT
TAAGGAGCTGCAACAACTGGCGAATGAAATTCGAACAGAACTATCTTCTATAATGTCGGGGACTCACAAATCGTT
AAGGGCCAGCTTGGCCTCTGTGGAACTGACGGTTGCCATTCATCACGTTTTTCACGCACCAGTGGATAAGATCCT
GTGGGATGTTGGTGAACAAACATATGCGCATAAAATTCTCACAGGAAGGCGACCCCTTATGCATACTCTAAGACA
AAAGAACGGTCTTTCCGGGTGTACATGTCGATCTGAAAGCGAATATGATCCATTTGGAGCAGGGCATGGATGCAA
TAGTGTTTCTGCTGGGCTTGGGATGGCGGTTGCACGTGATATAAAAGGGAAACGAGAGCGAGTTGTTACAGTGAT
CAGCAATGGGACAACTATGGCTGGTCAGGTCTATGAAGCAATGAGCAATGCTGGCTATTTAGACTCTAATATGAT
AGTCATCTTAAATGACAGCCGCCACACTTTGCACCCAAAAGTCGAAGACGGATCCAAAACTTCGATTAATGCTCT
ATCTAGTACCCTCAGCAAGCTTCAGTCAAGTAAATCATTTCGCAGGTTCAGGGAAGTTGCCAAGGGCGTAACGAA
GAGAATCGGTATGGGTATGCATGAATTAGCAGCAAAAGTTGATGAATATGCACGTGGTATGATCGGTCCTTTAGG
AGCTACCCTTTTTGAAGAACTTGGTTTGTATTACATAGGCCCTGTTGATGGGCACAATATCGAGGACCTTGTTTG
TGTCCTGCAGCAAGTGGCGTCTCTAGATTCAATGGGTCCTGTCCTTGTACATGTTATCACTGAGGAAAATCGAGA
ACCGGTAGACAAGAAAAACAACGAGGCACTAGGAATCATGGAGAAAGGTTTGTGTAAGCCAGATTCCTATCTTTA
CAGTGTTCTTACTCGTACATACAGTGATTGCTTCGCTGAGGCTTTGGTTATGGAGGCCGAGAAGGACAAAGATAT
AGTGATTGTTCATGCAGGAATGGAAATGGAAACATCTTTTCAGCTATTTCAGGAAAGATTTCCCGATAGGTTTTT
CGATGTAGGAATGGCGGAACAGCATGCTGTTACTTTTTCTGCTGGTTTAGCTTGTGGAGGCTTGAAACCGTTTTG
CATAATTCCGTCCACTTTTCTTCAAAGAGCATATGACCAGGTGGTCCAAGATGTAGATAAGCAGAGACTACCAGT
TCGTTTTGTCATCACAAGTGCAGGACTAGTGGGATCTGATGGTCCTGCGCAATGTGGGGCGTTCGATGTAACATT
CATGTCATGCTTACCTAACATGATCGTGATGGCGCCATCCGATGAGGATGAGCTTGTGGATATGGTGGCCACTTC
AACCCAAATTGATGATCGTCCAGTATGCTTTCGTTACCCGAGAGGTGCCATTGTTGGGTCAGACTATTATGCACG
CACTGGAATTCCACTTGAGATTGGAAAGGGAAAAGTTGTTATAGAGGGAAACAAAGTCGCTTTGCTCGGATACGG
TACAATGGTTCAAAACTGCCTCAAAGCCCGACACCTTCTCTCCAAGCTCGGGATCGAGGTAACAGTTGCTGACGC
CAGATTCTGCAAGCCACTCGATATGAATCTCGTCAGACAACTATGCGAAAGCCACGAGTTTCTTGTCACAGTCGA
AGAAGGCTCGATCGGAGGGTTCGGATCCCACGTGGCACAGTTCATTTCACTCGATGGGCAGCTCGATGGCAGGGT
CAAGTGGAGACCGGTTGTTCTGCCTGATAGTTATATAGAACACGCTTTGCCTAAAGAACAGCTTGCTCTCGCTGG
GCTTACTGGACATCACATTGCTGCAACTGTGTTGAATCTTCTGGGCCGAACCCGAGAAGCTCTTCTTTTGATGTG
TTAGAAATCTTCGAGTTTTCCCGGGATAGTTTTTATGTAATTAAGGTAATAACAATATCGGGTTTTATCGGTGCA
GAGGATTGAATTGTGTGTACAGAAGTGCAGGTTGATTGATTCATAGATCGATCTTGCTGTAAATTCAATGCGAAC
TCAGTTTGGCGGGTTATCCATTTTCTGCCAGCGAGCTGATGAATTTGTCTGATCAATCACTTTTAGGCCAACTCC
TGCCCTTAGTAAAATGTA

>PB.5901.2 Euphorbia maculata DOXP synthase Para.1_Iso.2 mRNA
ATAGCCAAACAAAAGAGACAAAAATGGAAGTCACAGAGCAAACAAAAGACGGCCAAAAGCCAACAACGAGATTCT
CAATTCTCAAAGCACCAAATCAATAATCGCAAATGAAAACCGATTCCACCAATCTTCAGAATTATGCTCCCTTTG
TCTGATTGTCTTCTCAATTTACCCATTTTCTACTGATTTCTTCGTTTTCTGGCCTTTGTTTTGTGTGTGATTTCT
TATCCATTTCTGTCACTTTTTCTTCAGCTCTCGAAGTTACCCACCATTGATCATTGTCTTGTTGATGAGCTCTCA
AATTACCCATTCGATTTGTGCTGAAATTTGGGGGCTAATCTCTCTGTTTTGCTATGGCTGCTGCTTCAGCTCAGT
ACACCAATGGAATCGCTGCAAATTCGTGTAAGAGATTTGGTCCCATTAGGGATTTTTCGGTCTCCGATTTCCCTT
GTAAAGCTGGGTTTTCGAGAACTGATTTCAATCCGAGCTCTTCTTCCATCTCCCATTCCAAGGGTTTTGTGAATC
GAATATGTTCGGTGCCTGATCTTGATGATATTTTCTGGGAGAAAACTCCTACTCCAATGCTTGATTTAGTTGAAA
ACCCTATTCATTTGAAGAATCTATCAATTAAGGAGCTGCAACAACTGGCGAATGAAATTCGAACAGAACTATCTT
CTATAATGTCGGGGACTCACAAATCGTTAAGGGCCAGCTTGGCCTCTGTGGAACTGACGGTTGCCATTCATCACG
TTTTTCACGCACCAGTGGATAAGATCCTGTGGGATGTTGGTGAACAAACATATGCGCATAAAATTCTCACAGGAA
GGCGACCCCTTATGCATACTCTAAGACAAAAGAACGGTCTTTCCGGGTGTACATGTCGATCTGAAAGCGAATATG
ATCCATTTGGAGCAGGGCATGGATGCAATAGTGTTTCTGCTGGGCTTGGGATGGCGGTTGCACGTGATATAAAAG
GGAAACGAGAGCGAGTTGTTACAGTGATCAGCAATGGGACAACTATGGCTGGTCAGGTCTATGAAGCAATGAGCA
ATGCTGGCTATTTAGACTCTAATATGATAGTCATCTTAAATGACAGCCGCCACACTTTGCACCCAAAAGTCGAAG

ACGGATCCAAAACTTCGATTAATGCTCTATCTAGTACCCTCAGCAAGCTTCAGTCAAGTAAATCATTTCGCAGGT
TCAGGGAAGTTGCCAAGGGCGTAACGAAGAGAATCGGTATGGGTATGCATGAATTAGCAGCAAAAGTTGATGAAT
ATGCACGTGGTATGATCGGTCCTTTAGGAGCTACCCTTTTTGAAGAACTTGGTTTGTATTACATAGGCCCTGTTG
ATGGGCACAATATCGAGGACCTTGTTTGTGTCCTGCAGCAAGTGGCGTCTCTAGATTCAATGGGTCCTGTCCTTG
TACATGTTATCACTGAGGAAAATCGAGAACCGGTAGACAAGAAAAACAACGAGGCACTAGGAATCATGGAGAAAG
GTTTGTGTAAGCCAGATTCCTATCTTTACAGTGTTCTTACTCGTACATACAGTGATTGCTTCGCTGAGGCTTTGG
TTATGGAGGCCGAGAAGGACAAAGATATAGTGATTGTTCATGCAGGAATGGAAATGGAAACATCTTTTCAGCTAT
TTCAGGAAAGATTTCCCGATAGGTTTTTCGATGTAGGAATGGCGGAACAGCATGCTGTTACTTTTTCTGCTGGTT
TAGCTTGTGGAGGCTTGAAACCGTTTTGCATAATTCCGTCCACTTTTCTTCAAAGAGCATATGACCAGGTGGTCC
AAGATGTAGATAAGCAGAGACTACCAGTTCGTTTTGTCATCACAAGTGCAGGACTAGTGGGATCTGATGGTCCTG
CGCAATGTGGGGCGTTCGATGTAACATTCATGTCATGCTTACCTAACATGATCGTGATGGCGCCATCCGATGAGG
ATGAGCTTGTGGATATGGTGGCCACTTCAACCCAAATTGATGATCGTCCAGTATGCTTTCGTTACCCGAGAGGTG
CCATTGTTGGGTCAGACTATTATGCACGCACTGGAATTCCACTTGAGATTGGAAAGGGAAAAGTTGTTATAGAGG
GAAACAAAGTCGCTTTGCTCGGATACGGTACAATGGTTCAAAACTGCCTCAAAGCCCGACACCTTCTCTCCAAGC
TCGGGATCGAGGTAACAGTTGCTGACGCCAGATTCTGCAAGCCACTCGATATGAATCTCGTCAGACAACTATGCG
AAAGCCACGAGTTTCTTGTCACAGTCGAAGAAGGCTCGATCGGAGGGTTCGGATCCCACGTGGCACAGTTCATTT
CACTCGATGGGCAGCTCGATGGCAGGGTCAAGTGGAGACCGGTTGTTCTGCCTGATAGTTATATAGAACACGCTT
TGCCTAAAGAACAGCTTGCTCTCGCTGGGCTTACTGGACATCACATTGCTGCAACTGTGTTGAATCTTCTGGGCC
GAACCCGAGAAGCTCTTCTTTTGATGTGTTAGAAATCTTCGAGTTTTCCCGGGATAGTTTTTATGTAATTAAGGT
AATAACAATATCGGGTTTTATCGGTGCAGAGGATTGAATTGTGTGTACAGAAGTGCAGGTTGATTGATTCATAGA
TCGATCTTGCTGTAAATTCAATGCGAACTCAGTTTGGCGGGTTATCC

>PB.5901.3 Euphorbia maculata DOXP synthase Para.1_Iso.3 mRNA
AAACAAAAGACGGCCAAAAGCCAACAACGAGATTCTCAATTCTCAAAGCACCAAATCAATAATCGCAAATGAAAA
CCGATTCCACCAATCTTCAGAATTATGCTCCCTTTGTCTGATTGTCTTCTCAATTTACCCATTTTCTACTGATTT
CTTCGTTTTCTGGCCTTTGTTTTGTGTGTGATTTCTTATCCATTTCTGTCACTTTTTCTTCAGCTCTCGAAGTTA
CCCACCATTGATCATTGTCTTGTTGATGAGCTCTCAAATTACCCATTCGATTTGTGCTGAAATTTGGGGGCTAAT
CTCTCTGTTTTGCTATGGCTGCTGCTTCAGCTCAGTACACCAATGGAATCGCTGCAAATTCGTGTAAGAGATTTG
GTCCCATTAGGGATTTTTCGGTCTCCGATTTCCCTTGTAAAGCTGGGTTTTCGAGAACTGATTTCAATCCGAGCT
CTTCTTCCATCTCCCATTCCAAGGGTTTTGTGAATCGAATATGTTCGGTGCCTGATCTTGATGATATTTTCTGGG
AGAAAACTCCTACTCCAATGCTTGATTTAGTTGAAAACCCTATTCATTTGAAGAATCTATCAATTAAGGAGCTGC
AACAACTGGCGAATGAAATTCGAACAGAACTATCTTCTATAATGTCGGGGACTCACAAATCGTTAAGGGCCAGCT
TGGCCTCTGTGGAACTGACGGTTGCCATTCATCACGTTTTTCACGCACCAGTGGATAAGATCCTGTGGGATGTTG
GTGAACAAACATATGCGCATAAAATTCTCACAGGAAGGCGACCCCTTATGCATACTCTAAGACAAAAGAACGGTC
TTTCCGGGTGTACATGTCGATCTGAAAGCGAATATGATCCATTTGGAGCAGGGCATGGATGCAATAGTGTTTCTG
CTGGGCTTGGGATGGCGGTTGCACGTGATATAAAAGGGAAACGAGAGCGAGTTGTTACAGTGATCAGCAATGGGA
CAACTATGGCTGGTCAGGTCTATGAAGCAATGAGCAATGCTGGCTATTTAGACTCTAATATGATAGTCATCTTAA
ATGACAGCCGCCACACTTTGCACCCAAAAGTCGAAGACGGATCCAAAACTTCGATTAATGCTCTATCTAGTACCC
TCAGCAAGCTTCAGTCAAGTAAATCATTTCGCAGGTTCAGGGAAGTTGCCAAGGGCGTAACGAAGAGAATCGGTA
TGGGTATGCATGAATTAGCAGCAAAAGTTGATGAATATGCACGTGGTATGATCGGTCCTTTAGGAGCTACCCTTT
TTGAAGAACTTGGTTTGTATTACATAGGCCCTGTTGATGGGCACAATATCGAGGACCTTGTTTGTGTCCTGCAGC
AAGTGGCGTCTCTAGATTCAATGGGTCCTGTCCTTGTACATGTTATCACTGAGGAAAATCGAGAACCGGTAGACA
AGAAAAACAACGAGGCACTAGGAATCATGGAGAAAGGTTTGTGTAAGCCAGATTCCTATCTTTACAGTGTTCTTA
CTCGTACATACAGTGATTGCTTCGCTGAGGCTTTGGTTATGGAGGCCGAGAAGGACAAAGATATAGTGATTGTTC
ATGCAGGAATGGAAATGGAAACATCTTTTCAGCTATTTCAGGAAAGATTTCCCGATAGGTTTTTCGATGTAGGAA
TGGCGGAACAGCATGCTGTTACTTTTTCTGCTGGTTTAGCTTGTGGAGGCTTGAAACCGTTTTGCATAATTCCGT
CCACTTTTCTTCAAAGAGCATATGACCAGGTGGTCCAAGATGTAGATAAGCAGAGACTACCAGTTCGTTTTGTCA
TCACAAGTGCAGGACTAGTGGGATCTGATGGTCCTGCGCAATGTGGGGCGTTCGATGTAACATTCATGTCATGCT
TACCTAACATGATCGTGATGGCGCCATCCGATGAGGATGAGCTTGTGGATATGGTGGCCACTTCAACCCAAATTG
ATGATCGTCCAGTATGCTTTCGTTACCCGAGAGGTGCCATTGTTGGGTCAGACTATTATGCACGCACTGGAATTC
CACTTGAGATTGGAAAGGGAAAAGTTGTTATAGAGGGAAACAAAGTCGCTTTGCTCGGATACGGTACAATGGTTC
AAAACTGCCTCAAAGCCCGACACCTTCTCTCCAAGCTCGGGATCGAGGTAACAGTTGCTGACGCCAGATTCTGCA
AGCCACTCGATATGAATCTCGTCAGACAACTATGCGAAAGCCACGAGTTTCTTGTCACAGTCGAAGAAGGCTCGA
TCGGAGGGTTCGGATCCCACGTGGCACAGTTCATTTCACTCGATGGGCAGCTCGATGGCAGGGTCAAGTGGAGAC
CGGTTGTTCTGCCTGATAGTTATATAGAACACGCTTTGCCTAAAGAACAGCTTGCTCTCGCTGGGCTTACTGGAC
ATCACATTGCTGCAACTGTGTTGAATCTTCTGGGCCGAACCCGAGAAGCTCTTCTTTTGATGTGTTAGAAATCTT

CGAGTTTTCCCGGGATAGTTTTTATGTAATTAAGGTAATAACAATATCGGGTTTTATCGGTGCAGAGGATTGAAT
TGTGTGTACAGAAGTGCAGGTTGATTGATTCATAGATCGATCTTGCTGTAAATTCAATGCGAACTCAGTTTGGCG
GGTTATCCATTTTCTGCCAGCGAGCTGATGAATTTGTCTGATCAATCACTTTTAGGCAACTCCTGCCCTTAGTAA
AATGTATAACATAGACCAGTACTCGAACTCTGGATGTAAGAGACTTAAGGCAACTTTTAACAGGGGCTCTTTATC
TAGTTAGCTAC

>PB.5902.1 Euphorbia maculata DOXP synthase Para.2 mRNA
CCAAACAAAGAGACAGTAATGAAAGTTACAGAGCAAACAAAAGACGGCCAAAAGCCAACAATGAGATTCTCAATT
CTCAAAGCAACCAAATCAATAATCGCTAATTGAAATTGATTCCACCAATTCTCTGAATTATACCCCCTTTGTCTG
ATTCTCTTCTCAATTCACCCAATTTCCACTGATTTCTCCGTTTCTTGGCCTTTGTTTTGTGTGTGATTTCTTATC
CATTTCTGTCACTTTTTCTTCAGCTCTCGAAGTTACCCACCATTGATCTTTGTCCTGTTGATGAGCTCACAAACT
GCCCTATTCAATTCGTGCTGAAATTTGGGGGGGCTAATCTCTCTGTTTTGCTATGGGTGCTGCTTCAGCTCAGTA
CACCAATGGAATCGCTGCAAATTCCTGTACGAGATTTGGTCTCGACAGGGATTTTTCCGTCTCCGATTTCCCTTG
TAAAGCTGGGTTTTCGAGAACTGATTTCAATCAAAGCTCTTCTTCTGTCACCAATTCCAAGGGGTTTGTGAATCG
AATATGTTCGGTGCCTGATCTTGATGATATCTTTTGGGAGAAAACTCCTACTCCTATGCTTGATTTAGTTGAAAA
CCCTATTCATTTGAAGAATCTATCAATTAAGGAGCTGCAGCAACTGGCAAACGAAATTCGAACAGAACTGTCTTC
TGTAATCTCGGGGACTCACAAATCATTAAGGGCTAGTTTAGCCTCTGTGGAATTGACAGTTGCCATTCATCACGT
TTTTCATGCTCCAGTGGATAAGATCCTGTGGGATGTCGGTGAACAAACATATGCACATAAAATTCTCACAGGAAG
GCGACCCCTTATGCATACTTTAAGACAAAAGAACGGTCTTTCCGGGTTTACATGTCGATCCGAAAGCGAATATGA
TCCTTTTGGAGCAGGGCATGGATGCAATAGTGTTTCTGCTGGGCTTGGGATGGCGGTTGCACGTGATATAAAAGG
GAAGCGAGAGCGAGTTGTTACAGTGATCAGCAATGGGACAACTATGGCTGGTCAGGTCTATGAAGCAATGAGCAA
TGCCGGCTATTTGGACTCTAATATGATAGTCATCTTAAATGACAGCCGCCACGCTTTGCACCCGAAAATTGAAGA
CGGTTCTAAAACTTCAATTAATGCTCTATCTAGTACCCTCAGCAAGCTTCAGTCAAGTAAATCATTTCGAAGGTT
TAGGGAAGTTGCCAAGGGCGTAACGAAGAGGATTGGTATGGGTATGCATGAATTAGCAGCAAAAGTTGATGAATA
TGCACGTGGTATGATCGGTCCTTTAGGAGCTACCCTTTTTGAAGAACTTGGTTTGTATTACATAGGCCCTGTTGA
TGGGCACAATATCGAGGACCTTGTTTGTGTCCTCCAGCAAGTGGCATCTCTAGATTCAATGGGTCCTGTCCTTGT
ACATGTTATCACTGAGGAAAATCGAGAACCGGTAGACAAGAAAAACAATGAGGCACTTGGAATCAAGAAGGAAGG
TTCATGTAAGCCAGATTCCTATCTTTACAGTGTTCTTACTCGTACATATAGCGATTGCTTTGCTGAGGCTTTGGT
TATGGAGGCAGAGAAGGACAAAGATATAGTGATTGTTCATGCCGGAATGGAAATGGAAACATCTTTTCAGCTATT
TCAGGAAAGATTTCCCGATAGGTTTTTCGATGTTGGAACAGCTGAACAGCACGCTGTTACCTTTTCCGCTGGTTT
AGCTTGTGGAGGCATGAAACCATTTTGCATCATTCCTTCCACTTTTCTACAAAGAGCATATGATCAGGTGGTCCA
AGATGTAGATAGGCAGAGAATACCGGTTCGTTTCGTCATCACAAGTGCAGGACTAGTAGGATCCGATGGGCCTGC
GCAATGCGGAGCATTCGATGTTACATTCATGTCATGCTTACCAAACATGATCGTGATGGCACCGTCCGATGAGGA
TGAGCTTGTTGATATGGTGGCCACTTCAACCCTAATTGATGATCGTCCAATTTGCTTCCGCTACCCGAGAGGTGC
CATTGTTGGGTCAGACTATTATTCTCGCACTGGAATTCCACTCGAGATTGGAAAGGGAAAAGTAGTTATCGAGGG
AAAAAACGTCGCTTTGCTAGGATACGGTACGATGGTCCAAAACTGCCTCAAAGCCCGACACCTTCTCTCCAAGCT
CGGGATCGAGGTAACCGTTGCTGACGCCAGATTCTGCAAGCCCCTCGATATGAATCTCGTCAGACAATTATGCCA
AAGCCACGAATTTCTTGTCACAGTCGAAGAAGGCTCAATCGGAGGGTTCGGGTCCCATGTGGCACAGTTCATTTC
TCTCGATGGACAGCTCGATGGTCGGGTAAAGTGGAGACCGGTTGTTCTGCCTGATAGCTATATAGAACATGCATT
GCCAAAAGAACAGCTCGCACTTGCTGGACTTACTGGGCATCACATAGCTGCAACCGTGTTGAATCTTCTGGGCCG
AACCCGAGAAGCTCTGCTTTTGATGTGTTAGAAATGATCTGGTTTTCCTGGGATGGTGTTTATGTAGTTAAGGTG
ATAACAGTATCGGGTTTTATTGATGCTGAGGTTCGAATTGTGTGTACAGAAGTGCATATTGATTGATTGAGAGGT
CGATCTCACTGTAAATTCAATGCGAACTAATTTGGCAGTTTATCCATTTTCTGCAAACAAGTCG

>PB.12314.1 Euphorbia maculata DOXP reductoisomerase Para.1 mRNA
GAGCTCTTGCAAGTTGCAATTCCCCCATTTCCAGTTCTTCATTTGAATCCATGGCTCTCAATCTGCTCTCTCCCG
CCGAGGTCAAGTCCCTCTCCTTCTTGGACTCTGCTAAGTCCAATTATCACCTCCCCAAGCTCTCAGGTGGATTGA
GTTTTAAGAGGAAGGAATTTGGAATGGCGTCTGGTGGGAGGAGAGTGCTTTGTTCGGCTCAAACTCCGCCGCCTC
CTGCCTGGCCGGGAACCGCTATGCCGGAGCCGGGCCGGAAGAGTTACGATGGTCCAAAGCCGATTTCGATTGTTG
GATCTACTGGCTCAATTGGGACTCAGACATTGGATATTGTGGCGGAGAATCCGGATAAATTTAGAGTCGTTGCAC
TTGCTGCCGGTTCTAATGTTACTCTTCTTGCTGATCAGGTGAAAACATTCAAACCTCAGCTTGTTGCTGTGAGGA
ACGAGTCACTAATTGATGAACTTAAAGAGGCTTTGGCTGATCTAGATGAGAAGCCTGAGATTATTCCAGGGGAGC
AAGGAGTTGTTGAGGTTGCTCGCCATCCCGATGCTGTCAGCGTGGTCACAGGAATAGTTGGTTGTGCCGGCCTCA
AACCTACGGTGGCTGCAATAGAAGCTGGGAAGGACATATGCTTAGCCAATAAAGAGACTTTGATAGCTGGAGGTC
CTTTTGTCCTTCCTCTTGCTCAAAAGTACAACGTCAAAATTCTTCCCGCTGACTCAGAGCATTCAGCTATATTTC
AGTGTATTCAGGGCCTGCCAGAGGGTGCACTTAGGCGTATTATTTAACTGCTTCTGGTGGGGCTTTCAGGGACT

GGCCAGTTGAGAAATTGAAAGATGTTAAAGTAGCTGATGCTTTGAAGCATCCCAACTGGAGCATGGGGAAAAAGA
TTACCGTGGACTCTGCTACCCTTTTCAATAAGGGTTTAGAGGTTATTGAAGCCCATTACTTGTACGGAGTTGACT
ATGACAATATTGAGATAGTAATTCATCCTCAATCCATCATTCATTCAATGATTGAAACACAGGATTCATCTGTGC
TTGCACAACTGGGGTGGCCTGACATGCGATTACCAATTCTATACACAATGTCTTGGCCCGAGAGAGTATATTGCT
CTGAAGTCACCTGGCCTCGCCTTGATCTTTGCAAGCTTGGGTCTCTAACATTCAAAGCTCCCGACAATGTAAAAT
ACCCGTCAATGGATCTGGCCTATGCTGCTGGACGTGCCGGAGGCACAATGACCGGAGTCCTCAGTGCCGCCAATG
AGAAAGCCGTGGAAATGTTCATCGATGAAAAGATAAGCTACCTTGATATATTCAAAGTTGTGGAGCAAACATGTG
AGAAGCATCAAGAAGAATTGGTGGCCTCTCCCTCTCTAGAGGAGATTATACATTATGATTTATGGGCCAGAGATT
ATGCTTCGAAATTGCAGCCGTCTTCGGGTCTAAGTCCTGTTCTTGCTTGATTGTTAACTAGCTAGTAATGGTAGC
CAGTTGTTTTGTGTACAAATGGTGAAATGGGCAATTTCAGTGTTTGTGTTCTTGATCCATGGTTTTTGTGTTCTG
TAATTGCTTTCTTACATAATGAAAAGCTGAGATCAAATCCATTTCCAAATGCATGGGAAGTGATTCAATTTTGTA
GAGATTGAAAATGAAACTGCATTTTGGTATCCTTAGGCTTCCATTTTTTAAGTTCTGGCAGTTTTGTATTGACAA
TTAAAGGGGTGTTTCTCGAACCTTTTAAGCACCTTATAAGCACTTTTTAGTTTATAAGTCATCTTGGGTCATGTT
CTTTTGGTTG

>PB.12314.2 Euphorbia maculata DOXP reductoisomerase Para.2 mRNA
GAGCTCTTGCAAGTTGCAATTCCCCCATTTCCAGTTCTTCATTTGAATCCATGGCTCTCAATCTGCTCTCTCCCG
CCGAGGTCAAGTCCCTCTCCTTCTTGGACTCTGCTAAGTCCAATTATCACCTCCCCAAGCTCTCAGGTGGATTGA
GTTTTAAGAGGAAGGAATTTGGAGTGGCGTCTGGTGGGAGGAGAGTGCTTTGTTCGGCTCAAACTCCGCCGCCTC
CTGCCTGGCCGGGAACCGCTATGCCGGAGCCGGGCCGGAAGAGTTACGATGGTCCAAAGCCGATTTCGATTGTTG
GATCTACTGGCTCAATTGGGACTCAGACATTGGATATTGTGGCGGAGAATCCGGATAAATTTAGAGTCGTTGCAC
TTGCTGCCGGTTCTAATGTTACTCTTCTTGCTGATCAGGTGAAAACATTCAAACCTCAGCTTGTTGCTGTGAGGA
ACGAGTCACTAATTGATGAACTTAAAGAGGCTTTGGCTGATCTAGATGAGAAGCCTGAGATTATTCCAGGGGAGC
AAGGAGTTGTTGAGGTTGCTCGCCATCCCGATGCTGTCAGCGTGGTCACAGGAATAGTTGGTTGTGCCGGCCTCA
AACCTACGGTGGCTGCAATAGAAGCTGGGAAGGACATATGCTTAGCCAATAAAGAGACTTTGATAGCTGGAGGTC
CTTTTGTCCTTCCTCTTGCTCAAAAGTACAACGTCAAAATTCTTCCCGCTGACTCAGAGCATTCAGCTATATTTC
AGTGTATTCAGGGCCTGCCAGAGGGTGCACTTAGGCGTATTATTTTAACTGCTTCTGGTGGGGCTTTCAGGGACT
GGCCAGTTGAGAAATTGAAAGATGTTAAAGTAGCTGATGCTTTGAAGCATCCCAACTGGAGCATGGGGAAAAAGA
TTACCGTGGACTCTGCTACCCTTTTCAATAAGGGTTTAGAGGTTATTGAAGCCCATTACTTGTACGGAGTTGACT
ATGACAATATTGAGATAGTAATTCATCCTCAATCCATCATTCATTCAATGATTGAAACACAGGATTCATCTGTGC
TTGCACAACTGGGGTGGCCTGACATGCGATTACCAATTCTATACACAATGTCTTGGCCCGAGAGAGTATATTGCT
CTGAAGTCACCTGGCCTCGCCTTGATCTTTGCAAGCTTGGGTCTCTAACATTCAAAGCTCCCGACAATGTAAAAT
ACCCGTCAATGGATCTGGCCTATGCTGCTGGACGTGCCGGAGGCACAATGACCGGAGTCCTCAGTGCCGCCAATG
AGAAAGCCGTGGAAATGTTCATCGATGAAAAGATAAGCTACCTTGATATATTCAAAGTTGTGGAGCAAACATGTG
AGAAGCATCAAGAAGAATTGGTGGCCTCTCCCTCTCTAGAGGAGATTATACATTATGATTTATGGGCCAGAGATT
ATGCTTCGAAATTGCAGCCGTCTTCGGGTCTAAGTCCTGTTCTTGCTTGATTGTTAACTAGCTAGTAATGGTAGC
CAGTTGTTTTGTGTACAAATGGTGAAATGGGCAATTTCAGTGTTTGTGTTCTTGATCCATGGTTTTTGTGTTCTG
TAATTGCTTTCTTACATAATGAAAAGCTGAGATCAAATCCATTTCCAAATGCATGGGAAGTGATTCAATTTTGTA
GAGATTGAAAATGAAACTGCATTTGGT

>PB.12314.3 Euphorbia maculata DOXP reductoisomerase Para.3 mRNA
GAGCTCTTGCAAGTTGCAATTCCCCCATTTCCAGTTCTTCATTTGAATCCATGGCTCTCAATCTGCTCTCTCCCG
CCGAGGTCAAGTCCCTCTCCTTCTTGGACTCTGCTAAGTCCAATTATCACCTCCCCAAGCTCTCAGGTGGATTGA
GTTTTAAGAGGAAGGAATTTGGAGTGGCGTCTGGTGGGAGGAGAGTGCTTTGTTCGGCTCAAACTCCGCCGCCTC
CTGCCTGGCCGGGAACCGCTATGCCGGAGCCGGGCCGGAAGAGTTACGATGGTCCAAAGCCGATTTCGATTGTTG
GATCTACTGGCTCAATTGGGACTCAGACATTGGATATTGTGGCGGAGAATCCGGATAAATTTAGAGTCGTTGCAC
TTGCTGCCGGTTCTAATGTTACTCTTCTTGCTGATCAGGTGAAAACATTCAAACCTCAGCTTGTTGCTGTGAGGA
ACGAGTCACTAATTGATGAACTTAAAGAGGCTTTGGCTGATCTAGATGAGAAGCCTGAGATTATTCCAGGGGAGC
AAGGAGTTGTTGAGGTTGCTCGCCATCCCGATGCTGTCAGCGTGGTCACAGGAATAGTTGGTTGTGCCGGCCTCA
AACCTACGGTGGCTGCAATAGAAGCTGGGAAGGACATATGCTTAGCCAATAAAGAGACTTTGATAGCTGGAGGTC
CTTTTGTCCTTCCTCTTGCTCAAAAGTACAACGTCAAAATTCTTCCCGCTGACTCAGAGCATTCAGCTATATTTC
AGTGTATTCAGGGCCTGCCAGAGGGTGCACTTAGGCGTATTATTTTAACTGCTTCTGGTGGGGCTTTCAGGGACT
GGCCAGTTGAGAAATTGAAAGATGTTAAAGTAGCTGATGCTTTGAAGCATCCCAACTGGAGCATGGGGAAAAAGA
TTACCGTGGACTCTGCTACCCTTTTCAATAAGGGTTTAGAGGTTATTGAAGCCCATTACTTGTACGGAGTTGACT
ATGACAATATTGAGATAGTAATTCATCCTCAATCCATCATTCATTCAATGATTGAAACACAGGATTCATCTGTGC
TTGCACAACTGGGGTGGCCTGACATGCGATTACCAATTCTATACACAATGTCTTGGCCCGAGAGAGTATATTGCT

CTGAAGTCACCTGGCCTCGCCTTGATCTTTGCAAGCTTGGGTCTCTAACATTCAAAGCTCCCGACAATGTAAAAT
ACCCGTCAATGGATCTGGCCTATGCTGCTGGACGTGCCGGAGGCACAATGACCGGAGTCCTCAGTGCCGCCAATG
AGAAAGCCGTGGAAATGTTCATCGATGAAAAGATAAGCTACCTTGATATATTCAAAGTTGTGGAGCAAACATGTG
AGAAGCATCAAGAAGAATTGGTGGCCTCTCCCTCTCTAGAGGAGATTATACATTATGATTTATGGGCCAGAGATT
ATGCTTCGAAATTGCAGCCGTCTTCGGGTCTAAGTCCTGTTCTTGCTTGATTGTTAACTAGCTAGTAATGGTAGC
CAGTTGTTTTGTGTACAAATGGTGAAATGGGCAATTTCAGTGTTTGTGTTCTTGATCCA

>PB.12315.1 Euphorbia maculata DOXP reductoisomerase Para.2 mRNA
GAGCTCTTGCATATTGCAATTCCCCCTTTTCCCATTCTCCATTTCAATCCATGGCTCTCAATCTGCTCTCTCCCG
CCGAGGTCAAGTCCCTCTCCTTCTTGGACTCCGCTAAGTCCAATCATCACCTGCCCAAGCTCTCAGGTGGGTTGA
GCTTTAAGAGGAAGGAATTTGGAGTGGCGGGGAGGAAAGTGGTTTGTTCGGCGCAAACTCCTCCGCCTCCTGCTT
GGCCGGGAACTGCTATGCCGGAGCCGGGCCGGAAGAGTTACGACGGCCCAAAGCCGATTTCGATTGTTGGGTCTA
CCGGCTCAATTGGGACTCAGACATTGGATATTGTGGCTGAGAATCCGGATAAATTTAGAGTCGTTGCACTTGCTG
CTGGTTCCAATGTTACTCTTCTTGCTGATCAGGTAAAAACATTCAAACCTCAGCTTGTTTCTGTGAGGAATGAGT
TGCTAATTGATGAACTTAAAGAGGCTTTGGCTGATCTAGATGAGAAGCCAGAGATTATTCCAGGGGAACAAGGAG
TTGTTGAGGTTGCTCGCCATCCAGATGCTGTCAGCGTGGTCACAGGAATAGTTGGTTGTGCCGGCCTAAAACCTA
CGGTGGCTGCTATAGAAGCTGGAAAGGACATATGCTTAGCCAATAAAGAGACTTTGATAGCTGGAGGTCCTTTTG
TCCTTCCTCTTGCTCAAAAGTACAATGTCAAAATTCTTCCTGCTGATTCCGAGCATTCGGCAATCTTTCAGTGTA
TTCAAGGCCTGCCAGAGGGTGCACTTCGGCGTATCATTTTAACTGCTTCTGGTGGGGCTTTCAGGGACTGGCCGG
TTGAGAAATTGAAAGATGTAAAAGTAGCTGATGCGTTAAAGCATCCCAACTGGAGTATGGGGAAAAAGATCACCG
TGGACTCTGCTACCCTTTTCAATAAGGGTTTAGAGGTTATTGAAGCCCATTACTTGTATGGAGTTGATTATGATA
ATATTGAGATAGTAATTCATCCTCAATCTATCATTCATTCAATGATTGAAACACAGGATTCATCTGTGCTTGCAC
AACTGGGGTGGCCTGATATGCGATTACCAATTCTATACACAATGTCTTGGCCTGAGAGAGTATATTGCTCTGAAG
TAACCTGGCCTCGCCTCGATCTTTGCAAGCTCGGGTCTCTAACATTCAAAGCTCCCGACAATGTAAAATACCCGT
CAATGGACCTCGCCTATGCTGCTGGGCGTGCCGGAGGCACAATGACTGGAGTCCTCAGTGCAGCCAATGAGAAAG
CTGTGGAAATGTTCATCGATGAAAAGATAAGCTACCTTGATATATTCAAGGTTGTGGCCCAAACATGTGAGAAGC
ATCAAGAAGAATTGGTGGCCTCTCCCTCTCTAGAGGAGATTATACATTATGATTTGTGGGCCAGAGATTATGCTT
CGAAATTGCAGCCGTCTTCGGGTCTTAGTCCTGTTCTTGCTTGATCGAAAGCTAGCTAGTAATGGTATTGGTGGC
TAGTTGTTTTGTGTACAAATAGTGAAATGGGCAGTTTCAGTGTTTGTGTTCTTGAATTGATCCATGGTTTTTGTG
TTGTGTAACTGCTTTCTTATAATGAAAAGCTGAGATCAAATCCATTTCCAAAATCATGGGAAGTGATTCAATTTT
GTAGAGATTGAAAATGAAACTGCATTTCGGTATCCTTAGGCTTCCATTTTTAAAGTTCTGGCAGTTTTGTATTGA
CAATTCAAGGTGTTTCCCAAAGACTATAAGCACTTTATAATTTATAAGTCATCTTAGGTCC

>PB.12317.1 Euphorbia maculata DOXP reductoisomerase Para.2_Iso.1 mRNA
AAGCATAAACGTAGCAAAGCAACCGCCAAAGGCAGCCACTGGTCACAATTGATTCACGTATGATTCCTTGTATTT
AAGCCTCTGCAATCCCCCTCTCTACTCGCTCTCAATTTCAATTTCAATTTATGGCTCTCAATTTACTTTCTCCCG
CCAAGGTCAAGGCTCTCTTCTTCTCGGATTCTCCCAAGTCCAATTCTCACCTCCCCAAGCTTTCAGGAGGACTGA
GTTTGAAGAGGAAGGATTTTGGAGCGAGGAAAGTGCAGTGTTCGGCTCAGCCTCCTCCACCGGCTTGGCCTGGGA
CAGCTTTACCAGAGCCTGGACGCAAGAGATACGACGGTCCAAAACCTATTTCAATCGTTGGATCTACTGGCTCTA
TTGGAACTCAGACATTGGATATTGTGGCAGAGAATCCAGATAAATTTAGAGTCGTTGCACTAGCCGCTGGTTCAA
ATGTTACTCTTCTTGCTGATCAGGTGAAGACATTTAAACCGCAGCTTGTTGCAATTAGGAACGAGTCATTAGTTG
ATGAACTTAAAGAGGCTTTGGCTGATCTAGATGAAAAGCCTGAGATTATTCCTGGGGAACAAGGAGTTGTCGAGG
TTGCTCGCCATCCAGATGCTGTCAGTGCGGTGACAGGAATAGTTGGTTGTGCAGGCTTAAAACCTACAGTGGCTG
CAATAGAAGCTGGAAAGGATATATGCTTGGCCAATAAAGAGACTTTAATCGCTGGAGGTCCTTTCGTCCTGCCTC
TTGCTCAAAAGTACAATGTAAAAATTCTTCCTGCTGACTCGGAACATTCTGCAATATTTCAGTGTATACAGGGCC
TGCCCGAGGGTGCACTTCGGCGAATTATTTTAACTGCTTCTGGTGGGGCTTTCAGGGACTGGCCTGTTGAAAAAT
TGAAAGAGGTAAAAGTAGCTGATGCTTTGAAGCACCCCAACTGGAGTATGGGAAAAAAGATCACTGTGGATTCTG
CTACACTTTTCAATAAGGGTTTGGAGGTTATTGAAGCCCATTACTTGTATGGAGTTGATTATGATAATATTGAGA
TAGTAATTCATCCTCAATCAATCATCCATTCAATGATTGAAACACAGGATTCATCAGTCCTTGCACAACTGGGGT
GGCCTGATATGCGTTTACCAATTCTATACACTATGTCTTGGCCTGAGAGAGTATATTGCTCCGAAGTAACCTGGC
CACGGCTTGATCTTTGCAAACTCGGGTCACTAACATTTAAAGCGCCTGATAATGTAAAATACCCATCCATGGATC
TCGCCTATGCTGCTGGACGTGCTGGAGGAACCATGACTGGAGTCCTCAGTGCAGCAAACGAGAAAGCTGTAGAAA
TGTTCATCGACGAAAAGATCAGCTACCTCGATATATTCAAGGTTGTAGAGCTAACATGTGAGAAGCATCAAGAGG
AACTGGTAACATCTCCCTCACTGGAAGAAATTATACACTATGACTTATGGGCTAGAGACTATGCTTCCAAGTTGC
GGACATCCTCCGGTCTAAGTCCGGTTCTTGCGTGATCATTGCCAGGAATGGTGGTAAGTTCTGTTATGTGGTATA
AATAGGTATTACATGGTTTAGCATAAACTGGGTTGTCCAGATGTATGTGTGCCTCCGCCTTTATGTGTTATTATG

TAATTGCTTCCTTAGGCAATGAAAGTCTGATTTTCCAAATTCACGGGGAGGCAAATGATTTGACTCTGGAGACTG
AAAGAACTTGAAATTGTATTTCGTGCTTAAAAAGGAAATGGATATTGAAGCTAAAGCGG

>PB.12317.2 Euphorbia maculata DOXP reductoisomerase Para.2_Iso.2 mRNA
GCTCTCAATTTCAATTTCAATTTATGGCTCTCAATTTACTTTCTCCCGCCAAGGTCAAGGCTCTCTTCTTCTCGG
ATTCTCCCAAGTCCAATTCTCACCTCCCCAAGCTTTCAGGAGGACTGAGTTTGAAGAGGAAGGATTTTGGAGCGA
GGAAAGTGCAGTGTTCGGCTCAGCCTCCTCCACCGGCTTGGCCTGGGACAGCTTTACCAGAGCCTGGACGCAAGA
GATACGACGGTCCAAAACCTATTTCAATCGTTGGATCTACTGGCTCTATTGGAACTCAGACATTGGATATTGTGG
CAGAGAATCCAGATAAATTTAGAGTCGTTGCACTAGCCGCTGGTTCAAATGTTACTCTTCTTGCTGATCAGGTGA
AGACATTTAAACCGCAGCTTGTTGCAATTAGGAACGAGTCATTAGTTGATGAACTTAAAGAGGCTTTGGCTGATC
TAGATGAAAAGCCTGAGATTATTCCTGGGGAACAAGGAGTTGTCGAGGTTGCTCGCCATCCAGATGCTGTCAGTG
CGGTGACAGGAATAGTTGGTTGTGCAGGCTTAAAACCTACAGTGGCTGCAATAGAAGCTGGAAAGGATATATGCT
TGGCCAATAAAGAGACTTTAATCGCTGGAGGTCCTTTCGTCCTGCCTCTTGCTCAAAAGTACAATGTAAAAATTC
TTCCTGCTGACTCGGAACATTCTGCAATATTTCAGTGTATACAGGGCCTGCCCGAGGGTGCACTTCGGCGAATTA
TTTTAACTGCTTCTGGTGGGGCTTTCAGGGACTGGCCTGTTGAAAAATTGAAAGAGGTAAAAGTAGCTGATGCTT
TGAAGCACCCCAACTGGAGTATGGGAAAAAAGATCACTGTGGATTCTGCTACACTTTTCAATAAGGGTTTGGAGG
TTATTGAAGCCCATTACTTGTATGGAGTTGATTATGATAATATTGAGATAGTAATTCATCCTCAATCAATCATCC
ATTCAATGATTGAAACACAGGATTCATCAGTCCTTGCACAACTGGGGTGGCCTGATATGCGTTTACCAATTCTAT
ACACTATGTCTTGGCCTGAGAGAGTATATTGCTCCGAAGTAACCTGGCCACGGCTTGATCTTTGCAAACTCGGGT
CACTAACATTTAAAGCGCCTGATAATGTAAAATACCCATCCATGGATCTCGCCTATGCTGCTGGACGTGCTGGAG
GAACCATGACTGGAGTCCTCAGTGCAGCAAACGAGAAAGCTGTAGAAATGTTCATCGACGAAAAGATCAGCTACC
TCGATATATTCAAGGTTGTAGAGCTAACATGTGAGAAGCATCAAGAGGAACTGGTAACATCTCCCTCACTGGAAG
AAATTATACACTATGACTTATGGGCTAGAGACTATGCTTCCAAGTTGCGGACATCCTCCGGTCTAAGTCCGGTTC
TTGCGTGATCATTGCCAGGAATGGTGGCAATGAAAGTCTGATTTTCCAAATTCACGGGGAGGCAAATGATTTGAC
TCTGGAGACTGAAAGAACTTGAAATTGTATTTCGTGCTTAAAAAGGAAATGGATATTGAAGCTAAAGCATAGAAT
GCTTGTGTATCTTTGTGCACTTTCTCTTTGGGAGATATGAGGCACCCGTAGCTCTCTTAATTTAATATTTGTTGA
TTCTGTACTTTTTGTGTAGTTAAATCCTTTCATTGCTCTATGTAGATTGGTATTTGAAAAATTTTTATTGAACGA
TTTTGGATTCAGTGATATTGGAAGGTGGTTTTAGTTT

>PB.14317.1 Euphorbia maculata CDP-ME synthase Para.1 mRNA
ATCCCAGGCACGTCCGTTTCTTCTTCTTCTCCCTCTCTTTTAAATCAACCCTGTTGCCTCTCTCGCCGTTGCTCT
CTGATAATCGTAAAAAAGATGCTTCTTTTGTGAATTTCGTCTCTAAAAGAGCGATCTTTGTTGATTTTATGGTCA
TGGATTTTGAAACGGAATCGCAGAAGAAGAAATTCGGGGTCTCGGCTAAGGTTTTCGGAGATGTCTACGGCGTTA
CTACTATGGTCCTCCCCAAAGAACACCTTCATCTCCATAAGATGGAGCCTTCTCCGCCGTCGCCTCCGCCTTTTC
ATCTCCGGCTTCAGCTCCCCGATCTCAGGAATTTGATTCGGAGCAGAGAGGCTGGCGAGTTTCTCAGCGGGGCTC
TAGCTGGTGCTATGACCAAGGCAGTACTTGCCCCTTTGGAGACTATCAGAACAAGAATGGTGGTTGGTGTTGGAT
CCAAGAACATTTCTGGTAGTTTCCTTGAGGTCGTTGAGCAGCAGGGTTGGCGAGGACTTTGGACTGGCAATGCAA
TTAATATGATCCGTATAGTGCCTACCCAGGCAATTGAGCTTGGAACATTTGAGTTTGTCCAACGTACTATGAAGT
CAGCGCAAGAAAAGTGGGATGAAGCTGGTGGTGCAAAAGTAAACATCGGTCCTGTCAGCTTGAACTTTTCACTCT
CCTGGTTATCCCCGACCGCTGTAGGTGGTGCTGCTGCTGGAATTGTTAGCACACTTGCATGCCACCCCCTTGAAG
TTTTGAAGGATCGACTAACAGTAAGTCCTAACGTGTACCCTAGTTTAAGAATTGCAATAAGCAAGATATATACTG
AGGGTGGGATAGGCGCCTTTTATTCCGGTCTCTCCCCCACATTGATTGGCATGCTTCCATACAGTACATGCTACT
ATTTCATGTATGAGACAATGAAGAAAAGTTACTGTAAATCCAAGAAAAAGAAGTCATTAAACCGTCCAGAGATGC
TCTTGGTTGGAGCTCTTGCAGGTTTTACAGCGAGCACAATCAGTTTCCCTCTGGAGGTTGCTAGGAAACGGCTAA
TGGTGGGAGCTTTGCAGGGCAAGTGCCCCCCTAACATGATTGCAGCACTCTCAGAAGTGATAAGGGAAGAGGGTT
TGATGGGACTTTACAGAGGGTGGGGTGCGAGTTGTTTGAAAGTCATGCCCTCTTCTGGTATCACTTGGATGTTCT
ATGAAGCTTGGAAAGATGTACTGCTGGCAGAAAGACGGCTTCTTTGATGGCCGCAGTAGCATCACTAGTTTCGGC
GTTCTCTTTTATGTATGGCAATTTTTATTCCTGACCTTAAATGAAGCCATTAGCAGGGCTTAAAATTTTGTATGA
ACGATATGCTGTCTGAATGCATTGAGAATCAGCATATGTTAGAAAATTTTGAATGTCTAACCGCATTGATTGCAA
TGAAGTGTAGCTGGTGAAATGATTATTTGGTTTTACAG

>PB.14318.1 Euphorbia maculata CDP-ME synthase Para.2 mRNA
AAAGAAAGATTGAGCATTTCAAAGAAAGCAAATCCAAAACCCTAAAAATCTCCAATGCCCAAAAAGTTCCCATCC
CAGGCACGTCTATTTCTTCTTCCTCCTCAAAACCCTCTTGCCTCTCCCGCCATTGCTCTCTGATAATCATAAAAC
AAATGCTTCTTTTGTGATTTTCGTTTCTAAAAGAGTGTCCTTTTTTAACTGACTGCATATACTGCTCTTGATCTT
ATAAACATGGATTTTGAAACGGAATCGCAGAAGAAGAAATTCGGAGTCTCGGCTAAGGTTTTCGGAGATGTCTAT

GGCGTTACTACGATGGTCCTCCCCAAAGACCACCTTCGCCTGCATACGATGGACCCTTCTCCGCCGTCGCCTCCG
CCTTTTCATCTCCGGTTTCAGCTCCCCGATCTCAGAAATTTGATTCGGAGCAGAGAGGCTGGCGAGTTTCTCAGC
GGGGCTTTAGCTGGGGCTATGACTAAGGCGGTACTTGCCCCCTTGGAGACTATCAGAACAAGAATGGTGGTTGGT
GTTGGATCCAAGAACATTTCTGGTAGTTTCATTGAGGTTGTTGAGCAGCAGGGTTGGCGAGGACTTTGGACCGGG
AATGCAATTAATATGATTCGTATTGTCCCTACCCAAGCAATCGAGCTTGGAACATTTGAGTTTGTCCAGCGTACT
ATGAAGTCAGCTCAAGAAAAGTGGAATGAAGCTGGTGGTGCAAAAGTAAACATCGGTCCCGTCAGCTTGAACTTT
TCACTCTCCTGGTTATCCCCAACCGCTGTTGGTGGTGCTGCTGCTGGAATTGTTAGTACACTTGCATGCCACCCC
CTTGAAGTTTTGAAGGATCGACTAACAGTAAGTCCAAATGTGTACCCTAATTTAAAAATTGCAATAAGCAAGATA
TACACCGAGGGTGGGATAGGCGCTTTTTATTCTGGTCTTTCTCCCACACTGATGGGCATGCTCCCATACAGTACA
TGCTACTATTTTATGTATGAGACAATGAAGAAAAGTTATTGTAAATCCAAGAAAAAGAAGGCGTTAAACCGTCCA
GAGATGCTCTTGGTTGGAGCTCTTGCAGGTTTTACAGCGAGCACAATCAGCTTTCCTCTGGAGGTTGCAAGGAAA
CGGCTAATGGTAGGGGCTTTGCAGGGCAAGTGCCCCCCTAACGTGATAGCAGCACTCTCAGAAGTGATAAGGGAA
GAGGGTTTGATGGGACTTTACAGAGGGTGGGGGGCGAGTTGTTTGAAGGTCATGCCATCCTCTGGTATCACTTGG
ATGTTCTATGAAGCTTGGAAAGATGTACTGCTAGCAGACAGACGGCTTCTATAATGGCTGCAGTAGCGTCACTAG
TTTTGGCCTTCTCCTTTAATGTATCCCAAATTTGATCCCTGGCCTTAAATGAAGCCATTAGCAGGGCTTAAAATT
TTGTATGAACTGAAATGATGTCCCAATGCGTTGAGAATCAGCATATTTTAG

>PB.13804.1 Euphorbia maculata CDP-ME kinase mRNA
ACTGTGTTGCAAGAAGAAGAAGACAAAGACATTAGAAAATATATCATGTCTCCGATGATTTGATTCCATCCTCTT
GCTCTGGCTGTTCGAATTTGACTGCGAAACCCCCGAAATGGATAATTTAATGCTCTAGTCTACTCTTTCAGGCCG
CCGTTTGATTTATAAAATCATTGTATCCGCAATTGCTCCCTTTGCTTCAGATTGCTGCTGCTATGGCTTCCGCTC
ATTTCCTCTGCAACAACCACCTCTTCCATCCTTCTTTCTCTAAATCCAATTCCCCTTCTTTCCGCCCGTTTAGCT
CCCTCTCCTTCGATCAAAGGTTAAGGGTTCAACCAGTTAAAGCCACCCAGAAGCAACTCGAGATAGTTTATGATC
CTGATGAAAGATTAAACAAGTTAGCGGATGAAGTGGATAAGAATGCTCCTCTTTCTAGGCTCACTCTGTTCTCTC
CTTGCAAGATTAATGTATTCCTCAGAATAACCAAAAAGAGAGAAGATGGTTATCATGACTTGGCATCTTTGTTTC
ATGTTATTAGTTTGGGAGATACAATTAAGTTCTCTCTGTCTCCTTCAAACAAGACTGATCGTTTATCTACTAATG
TATCTGGTGTGCCCCTAGATGAAAGGAATCTGATTATAAAAGCCCTCAATCTTTACAGGAAGAAAACCGGAACAG
ACCAGTTCTTTTGGATTCATCTAGATAAGAAGGTGCCTACAGGGGCAGGACTTGGCGGGGGAAGTAGTAATGCTG
CAACTGCCCTGTGGGCAGCAAATCAATTCAGTGGCGGTCTTGCCTCTGAAAAGGATCTGTTAGAATGGTCAAGTG
AGATTGGTTCAGATATCTCCTTCTTTTTCTCTCAAGGAGCTGCATATTGCACTGGCCGTGGTGAAATTGTTGAAG
ATACCCCTTCACCATTTCCTCGTGACCTTCCAATGGTTCTTATAAAACCTAAGGAGGCGTGTCCAACTGCAGAAG
TCTACAAGCATTTTCGATTGGACCAAACCAGTGACGTTGATCCTTTACACCTTCTGGAGAAGGTCTCAAAGTATG
GAATATCACAAGATGTTTGCATTAATGATTTAGAGCCTCCTGCATTTGAGGTTCTCCCATCACTAAAAAGATTGA
AACAGCGTATAGCTGCAGCTGGTCGGGGACAATATGATGCGGTCTTTATGTCTGGGAGTGGAAGTACGATTGTTG
GCATCGGCTCACCAGATCCTCCGCAATTCATTTATGATGATGATGAGTTCAAGGACGTATTTGTGTCAGAGGCCA
ACTTCTTGACTCGGGAGCCAAACCAGTGGTACAAACAGCCTGCTTCCACCACTGCTTGCAGCCCCCCTTCTCGAT
TCGAATCATGATTTCTTGTTTTCATTTCCTTGCTCTTCTTCATTCATACTGTTTTCTGTTGTAAGCTTTGCACCA
TTTTGAAGTACTACCGATGGAGCCGCCTAAATTTGTAACCTCCACTGTGCGAGGTAGGTTGTTGTAGATTGCCCA
CCTTTTGAATCAATATATAAAACTCTCAGTTACTTGTAATCAACAACTAAGAGTGAATAATGAATTCAGATTTTT
GAACCG

>PB.12927.1 Euphorbia maculata MECP synthase Para.1 mRNA
ATCTATTCAGCTGAGTCCACTAGTTTTTCCCCTCCGCAGAATTGACCACCGTCTCTTGTCTTCTCCGGCGAGCCA
TGGCCATTTCTCTCCAACTCTGCCGCCTCTCTCTCCGCGCCGATATATTTTCCGGCGACAAACTCGTCCCTCATT
TCCCCCGTAGATCGATCTCCATCCGATGCTCCGCCGGCGACTCCGCTTTGCCGTCGGTCTCCGGTGACTCCGATT
TCGACGCCAAGGTCTTCCGGCACAACTTGACTCGGAGCAAGAATTACAATCGGAGAGGATTTGGACACAAGGAGG
AGACCTTGGAGCTCATGAGTCAGGAATATACGAGTGATATTATCAAGACTTTGAGGGAAAACGGGAATGAATACA
CTTGGGGAAATGTTACTGTGAAATTGGCGGAAGCTTATGGGTTTTGCTGGGGCGTTGAGCGAGCTGTGCAGATTG
CTTACGAAGCTAGAAAGCAATTTCCGGAGGATAAGATTTGGATTACTAATGAAGTTATTCATAATCCAACTGTAA
ACAAGCGTTTGGAAGAGATGGAAGTTGTAACTATTCCTGTTGCAGAAGGGGGAAAGCAATTTGAAGTTGTGAACA
AAGGAGATGTTGTGATTTTGCCTGCTTTTGGAGCTGCAGTTGATGAGATGCTGACTCTAAGTCAAAAGGATGTTC
AAATTGTCGATACTACTTGCCCTTGGGTATCTAAGGTCTGGAATACGGTTGAGAAGCACAAGAAGGGAGACTATA
CTTCAATAATTCATGGTAAATATAGTCACGAGGAAACTATTGCTACTGCTTCTTTTGCGGGAAAGTACATCATAG
TGAAGAATATGGATGAGGCGATGTACGTTTGTGATTACATTCTTGGTGGTCAACTTAATGGCTCTAGCTCAACAA
GAGAAGCGTTCCTTAAGAAATTTGAAAAAGCAGTTTCCAAGGGATTTGATCCAGATGTTGACCTTGTCAAAGTCG
GTATTGCAAATCAAACTACAATGCTCAAGGGAGAAACAGAAGCCATTGGAAAATTGGCAGAGAAGACCATGATGC

AGAAATATGGTGTCGAAAATGTCAATGATCACTTTATTAGCTTCAACACCATTTGTGATGCTACTCAAGAGAGGC
AAGATGCAATGTATAAATTGGTAGAGGAAAAGCTGGATCTGATTCTGGTTGTTGGTGGATGGAACTCCAGCAACA
CTTCACACCTCCAAGAAATTGCAGAAGAGCGTGGAATTCCTTCATATTGGATCGACTATGAAAAGAGAATCGGTC
CAGGAAACAAAATCGCTTATAAGCTGAATCATGGGGAATTAGTTGAGAAAGCGAACTTTCTCCCAGAAGGTCCCA
TTACAATCGGTGTAACATCCGGTGCCTCAACTCCAGATAAGGTTGTTGAAGATGCGCTTGTGAAAATATTTGAAA
TCAAACGTCAAGAATTGTTGCAATTGGCATAGATTATGATTCTGGTAACAGACCCCGAGCATGAAATTAACATAA
GGTGCTGAAGATCTGTGTTGTACCATCAGAATGTACAAAAGGAAGAGCAATAATGTTGATAATATGCTTTGTATT
TGAGCTTATCTTATGTAAGAACTAATGTAAAATCCCAAATAAGGGTAGAGTCCGAAATTCATTGTACTAGTAAAC
CATCTCTTGTGTTGGTGTTGCATTTTTTGGTTAGAAAATTTCATATACACACCTTTACTATCG

>PB.12928.1 Euphorbia maculata MECP synthase Para.2 mRNA
ACAATAAAAACCCAAAGCCATAATCACAAAAAGGATCTCTTCAGCTGAGTCCACTGGTTTTGTTTCCCCCTCCGC
CCATTTGACCACCGTCTCTTGTCTTCTCCGGCGAGCCATGGCCATTTCTCTCCAACTCTGCCGCCTCTCCCTCCG
CGCCGACCTCTTTTCCGGCGACAAGCGCGTCCCTTGTTTCCCCCGTAAATCGATCTCCATCCGCTGCTCCGCCGG
CGACTCCGCTTCGCCGTCGGTCTCCGGTGACTCCGATTTTGATGCTAAGGTATTCCGGCACAACTTGACTCGGAG
CAAGAATTACAACCGGAGAGGATTCGGACACAAGGAGGAGACCTTGGAGCTCATGAATCAGGAATATACGAGTGA
TATTATCAAGACTTTGAAGGAAAACGGGAACGAATACACTTGGGGAAATGTTACTGTGAAATTGGCTGAAGCTTA
TGGGTTTTGCTGGGGCGTTGAACGCGCTGTGCAGATTGCTTATGAAGCTAGAAAGCAATTTCCTGAAGATAAGAT
TTGGATTACTAATGAAGTTATCCATAATCCGACTGTTAATCAGCGTTTGGAGGAGATGGAAGTTGTAACTATCCC
TGTTGCGGAAGGGGGAAAGCAATTTGAAGTTGTGAACAAAGGAGATGTTGTTATTTTGCCTGCTTTTGGAGCTGC
GGTAGACGAGATGTTGACTCTAAGTCAAAAGGATGTTCAAATTGTCGATACTACTTGCCCTTGGGTATCTAAGGT
CTGGAATACGGTTGAGAAGCACAAGAAGGGAGACTACACTTCAATAATTCATGGTAAATACAGTCACGAGGAAAC
TATTGCTACTGCTTCTTTTGCAGGAAAGTACATCATAGTGAAGAATATGGATGAGGCGATGTATGTTTGTGATTA
CATTCTTGGTGGTCAACTTAATGGTTCTAGCTCAACAAGAGAAGCTTTCCTTAAGAAATTTGAAAAAGCAGTTTC
CAAGGGATTTGATCCCGATGTTGACCTTGTCAAAGTCGGTATTGCAAATCAAACTACAATGCTCAAGGGAGAAAC
AGAAGCCATCGGAAAATTGGCGGAGAAGACCATGATGCAGAAATATGGTGTCGAAAATGTCAACGATCACTTTAT
TAGCTTCAACACCATCTGTGATGCTACTCAAGAGAGGCAAGATGCTATGTATAAATTGGTAGAAGACAAGCTGGA
TCTGATTCTAGTTGTTGGTGGATGGAACTCAAGCAACACTTCACACCTCCAAGAAATTGCAGAAGAGCGTGGAAT
TCCTTCATATTGGGTTGACTCTGAAAAGAGAATCGGTCCAGGAAACAAAATCGCTTATAAATTGAATCACGGGGA
ATTGGTTGAGAAAGAGAACTTCCTGCCAGAAGGCCCCATTACAATCGGTGTAACATCCGGTGCCTCAACTCCAGA
CAAGGTTGTTGAAGATGCGCTTGTGAAAATATTCGAAATCAAACGTCAAGAATTGTTGCAATTGGCATAAATGCG
TAAAATTAACAGAAGATTCTTAAGATCTGTGTTGTACCATCAGAATGTACAAAAGGAAGAACAATAATGCCGATA
ATGTGTTTGTTATTTCAGCTTATCTTGTCTTAGAACTAATGTAAAATCCCGAATAAGGTAGAGTCCGAAATTCAG
AGTACTAGCAAACAATCTCATGAATTGGTGTTGCATTCTTTGGTTAAAAAATTTCATATATATGCCTTTACTGT

>PB.18397.1 Euphorbia maculata MECP synthase Para.3 mRNA
ACCCCCAAAATCAGCTCTCTTCCTCTTCAATACAAACCACCATCGTCGTCTCCTTCTTCGAAGCCAATTACCCGC
CTTTCCAGCCGCCGGGCCAACAACTGTTCAAGCCGATGCAGCCGCAACTTCCGCCAAAGTCTCTGCCTTTTAGAG
TGGGTTATGGGTTCGATCTTCACCTTTGATCATTGGGGGGATTAATATACCGCATGAAAGAGGCTGTGAAGCTCA
CTCTGATGGAGATGTGTTGTTGCGGGATGCAATTCTGGGGGGTATTAGGGCTGCCTGATATTGGTCAAACATTCC
TAGATTCTGATCCTAAGTGGAAAGGAGCTGCGTCTTCTGTTTTCATCAAAGAAGCAAGTCTCCCGCCGAATCATT
CGCTCTTCTTCGATTTTCTAGATTCCGTTGAACCTTCTCCTGTAGTTGATCTTGAAGGACTGAGGCAAACTTGAC
TCAACTTCTAGTTCTACAAACGATGATATAAAACCTGGTTTGTTGACAGACTTCTTTTGTTCGTTTGAGAATGCT
GAGAACGTGAGTGCCAAAGCAGATTCAAGTCAGGGAGCTAGGTCCGGTGGTCAGAATGCTGCTTATGCTAATCAT
TCCGAACTGTTTCAGCTCCAGTTTATTGGGATTGGAAAATGAAAGGTTACATTACGCTTGAAGCTCATAAGAAGG
CAGAATCTGTGAAGAGTTCATTGAAAAGATTGAGTCTTGGCCGGATCAATGGCTTGTACCTTTCCAAAGATGATA
GGATTCTCACTGGGCTGCAGCCTAAACTCAAGTTCAGAAATACAGTGAAGCAATCAAAGATTGTCTCAAATCCAT
TGAGTTGACCCAAATTATATCAAGGCATACTCGTTTGGACTACATATTATGCCCAGGAGAACTCCAAAGATTTAG
GAAAGAATGAAGTCTGCCAACAAGGTCCGGACCTGGTGCACAAAGGAAAAGGGACCGGAGCCACAGGGCAAGCCT
CTGATCCAGAATCAAACATACAAAGAGTGGTCCAAAGTGTCTACAAAGCCAAAGACAAGTGAAAAAATTCATTTA
ATGTCTCTACTTAAATTAATCAGAAGCATTTATGATAAGAGAGACAACAAAAGAAAGTTGGGACCACTTTGAAAG
CGACATAAAGCAACCAAAGCCAATCCGGAAAAAGCTCAAGTCCAAAAATGGAAACATCCTGATATGAAGCAATCT
GAGAGCAGAGAAAGACATCTGATCCTCTTGCCAGTCAACCAAGTTTCAAACTCAAATTTACAACGAGCATTTAAT
TGAGTCTAACAGCTTTTTGGACGGTCCGGATACAAGATTCCAAATCAAATCAACGGACAAGATTGGTTCTTCATC
AACGGTCGCATTTATTGCTCAACCTATAAATACCAGAGCACAAGAAGTGAGGAAGCAGCTGATTCTTTCGAATAT
ATTTAGAGCGTTTGAATTCTGAAAGCAATCTGCGTTGAACATTCAAAGTGCAAGGTCCGGAGGGTAATCTGTGAG

CTTTGTTTTTGAGTGTTTGTTTGTTTCGCATTTCAATCTGGGGATTAAAGGTGCAGGGACTTAGTTTCTAGGATC
GGTGAGTGATCAGAAACGGTATATCCCATCTGTTATAGGATTTAGCTAAGTGGTGAGCTTAGTGGTATATCCTAC
CTCCGATAGATAGGGATCTTCGGTGATGTGATGTGACGGTTTGGAACCGAACTAAGAGCAAATGTGATCCTCTCA
TCAGCTTAGGTGCAAAGTACAAACCGTCTCCAGAAGAAGTAGCGGCTTCGTGTGATGTTACATTTGCAATGCTTG
CTGACCCGGAAAGCGCAGTGGAGGTTGCATGTGGAAAAAATGGAGTAGCAACCGGTATGAGTCCTGGAAAAGGAT
ATGTAGATGTCTCAACTGTTGATGGTGGCACATCGAAACTAATAGGCGAACATATCAAAGCTACTGGAGCATTGT
TTTTGGAGGCTCCTGTTTCTGGCTCCAAAAAACCGGCAGAAGACGGGCAGCTTATCTTTCTTACTGCAGGTGACA
AATCTCTGTATGAAAAAGCCGCTCCATTTCTAGACATCATGGGAAAGCAATTTTTAACAGTCCAGATTCTATCTC
GGTGAGGTCGGAAATGGAGCAGCTATGAAACTTGTGGTCAACATGGTCATGGGCAGTATGATGGCATCTTTTTCT
GAAGGGTTGCTTCTCAGCGAGAAAGTTGGGTTAGACCCTAATGTACTTGTTGAGGTAGTGTCACAGGGTGCGATT
AGTGCTCCGATGTACAAAATGAAAGGGCCGTCCATGATAAAATCTCAGTACCCACCCGCTTTCCCCTTGAAGCAT
CAACAAAAGGATTTGAGGCTCGCTCTCGGGCTAGCAGAATCGGTTTCTCAACCAACTCCAATTGCTGCTGCTACT
AATGAGCTCTATAAGGTCGCTAAAGGTCTCGGCCTTAGCGACAGCGATTTTTCTGCAGTTATTGAAGCACTTAAA
GCCAAACTGCAGTCTTGAGCATATGGTTGAATCGTGCGTTGGTTATTCGGTTTTGCTTGTTGTTAAGAGCTGTAA
CATTTACGACATTTGTTGTACCAATGTTATTAGAGGAAATTTTTTGATGTAGTTGAGATGGCGAGAATCAAAAGA
GATTTTGATCAAGGGTTACTTCATGTAGTTTGATATATTTAACAAAGGAACAAAGCCAAACTTTTATCTTATGTG
TAACAAAAAGACATTTATGAAACTTCGATGTGTTATGAGTAATTTAGAATTTAATTAATTTTTGCTTGTAGATCC

>PB.6487.1 Euphorbia maculata HMBPP synthase Para.1_Iso.1 mRNA
AAACTAATTCAGTCTTCAAATACAAATCCTCTGTGATTTTGTTTCCCTCAAATTCTTCACCAACACACGCCCTTC
TCTGCATTATCTCCTCTACTTCGTCTCCAGATCCTTCATTTCCCTTCGCTTTCTCTTCTCTCTTTGGTAGGTGAG
AAATGGCGACTGGAGCTGTGCCGGCGTCTTTTACTGGTCTGAAGACCAGAGAATCGAGCTTGGGGTTTGGGAAGA
GCTTAGATTTTGTCAGAGTTTGTGATTTGAAGAGGATCAAGTCCGGCAGGAAGAGGATTTCAATGATTCGTAACT
CAAATACGGGTCCTGAGATGGTTGAGCTTCAGCCTGCTTCAGAAGGAAGCCCTTTATTAGTTCCTCGACAAAAGT
ATTGTGAATCTATTCACAAGACTGTGAGGAGAAAAACCCGCACTGTGATGGTGGGAAATGTTCCTCTTGGTAGCG
AGCATCCTATCAGGATCCAAACTATGACTACTACTGACACCAAAGATGTTGCTGGAACAGTTGAACAGGTGATGA
GAATAGCAGATCAGGGGGCAGATATAGTTCGGATAACAGTTCAAGGAAAGAGAGAAGCAGATGCGTGCTTTGAAA
TTAAAAATTCTCTCGTGCAGAAAAATTACAATATCCCGCTGGTTGCTGATATTCATTTTGCTCCACCTATTGCAA
TGCGGGTTGCTGAATGCTTTGACAAAATTCGTGTAAATCCAGGGAATTTCGCTGATAGGCGGGCACAGTTTGAGA
AGCTGGAGTACACAGAAGACGACTATCAGAAAGAACTTGAGCATATTGAGCAGGTATTTACACCACTGGTTGAAA
AATGTAAGAAATATGGAAGGGCTATCCGCATTGGGACAAACCATGGGAGCCTTTCAGATCGTATCATGAGCTACT
ATGGAGATTCTCCAAGGGGAATGGTTGAATCTGCATTTGAGTTTGCAAGGATATGCCGGAAACTGGATTTCCACA
ATTTTGTCTTTTCAATGAAAGCAAGTAACCCAGTTATCATGGTCCAGGCCTACCGTCTCCTTGTTGCAGAAATGT
ATGTCCAAGGCTGGGATTATCCATTGCACTTGGGTGTCACTGAAGCTGGAGAAGGTGAAGATGGGCGTATGAAAT
CTGCAATTGGCATTGGAACATTACTTCAGGATGGTTTGGGTGACACTATCAGGGTGTCCCTGACAGAATCCCCAG
AGATGGAGATAGACCCTTGCAGAAGATTGGCCAACCTTGGTATGAGAGCATCTGCGGCTCAACAAGGAGTGGCAC
CATTCGAAGAAAAGCACAGGCACTATTTTGACTTCCAGCGCCGATCTGGTGACTTGCCAGCTCAAAAAGAGGGTG
AAGAGGTTGATTATAGAGGCGTCTTGCACCGTGATGGCTCGGTTCTTATGTCAGTATCCTTGGATCAGTTGAAGG
CACCCGAACTTCTATACAAGTCTCTTGCTGCAAAGCTTGTAGTTGGAATGCCTTTCAAGGACCTGGCAACTGTTG
ACTCAATCTTATTGAGAGAACTTCCACCAGTGGATGATGTTGATGCTCGGCTAGCTCTAAAAAGGCTGATAGATA
TTAGCATGGGAGTTATTGCCCCCTTATCAGAGCAGCTAACAAAGCCATTACCGAACGCCATGGTTCTTGTAAATC
TGAAGGAGTTGGCCAGTGGCGCTCACAAGCTTCTGCCTGAAGGTACACGCTTGGTTGTGTCTTTGCGTGGCGATG
AGTCTTATGAAGAACTATATATCCTCAGAGACATTGATGCTACAATGATTCTACATGACCTTCCATTTTCAGAAG
AGAAAGTTGGGAGAGTTCAAGCGGCAAGGAGACTATTTGAGTTCCTAGCAGACAATTCCTTGAATTTCCCAGTAA
TTCACCACATTCATTTTCCAAAAGGGATTCACAGGGACGACTTGGTCATTGGTGCTGGTACAAATGTGGGGGCCC
TACTGGTAGATGGTCTGGGAGATGGTTTACTATTAGAAGCCCAAGACCAGGACTTTGACTTCCTGAGGAACACGT
CTTTCAACCTATTACAAGGTTGCAGAATGAGAAACACAAAGACGGAGTACGTCTCATGTCCATCATGTGGTAGAA
CTTTGTTTGACCTTCAAGAAATCAGTGCAGAAATCCGTGAAAAGACATCTCATTTGCCTGGCGTCTCGATTGCAA
TTATGGGTTGCATTGTGAACGGACCCGGTGAGATGGCTGATGCTGATTTCGGGTATGTTGGTGGTGCTCCAGGGA
AGATTGACCTTTATGTCGGAAAGACGGTGGTGAAGAGAGGAATAGCAATGGAGGGAGCCACTGATGCATTGATCG
AGCTGATTAAAGAGCACGGGCGTTGGGTAGACCCTCCTGCAGAGGAGTAAGATCGAACATTTATAGTTCGGTTCA
TATGAACGGAGAGGCTTTAAAGGGCTTGTAAAAAGACCAAATCTTTTACAGTAGAAGGGTGTAGATTTGTATCCC
ATTGATGTTATCTACAGTTAATAATACTAGAAATGGTAGCAATGTAGTTACTTGAGAATAAGAAATCCAAGATTT
CATGATGGCTTTACTTGGCGAATCGAGCGGTGTAGTTTTATTTTAATGAAAATTGTACTCAACATTATAGTTTTC
GGATTTGGTTGTACAGTCGATGCTAGTTATTTTGAATAATAATTTATGCATTTAGCTTTT

>PB.6487.2 Euphorbia maculata HMBPP synthase Para.1_Iso.2 mRNA
AAACTAATTCAGTCTTCAAATACAAATCCTCTGTGATTTTGTTTCCCTCAAATTCTTCACCAACACACGCCCTTC
TCTGCATTATCTCCTCTACTTCGTCTCCAGATCCTTCATTTCCCTTCGCTTTCTCTTCTCTCTTTGGTAGGTGAG
AAATGGCGACTGGAGCTGTGCCGGCGTCTTTTACTGGTCTGAAGACCAGAGAATCGAGCTTGGGGTTTGGGAAGA
GCTTAGATTTTGTCAGAGTTTGTGATTTGAAGAGGATCAAGTCCGGCAGGAAGAGGATTTCAATGATTCGTAACT
CAAATACGGGTCCTGAGATGGTTGAGCTTCAGCCTGCTTCAGAAGGAAGCCCTTTATTAGTTCCTCGACAAAAGT
ATTGTGAATCTATTCACAAGACTGTGAGGAGAAAAACCCGCACTGTGATGGTGGGAAATGTTCCTCTTGGTAGCG
AGCATCCTATCAGGATCCAAACTATGACTACTACTGACACCAAAGATGTTGCTGGAACAGTTGAACAGGTGATGA
GAATAGCAGATCAGGGGGCAGATATAGTTCGGATAACAGTTCAAGGAAAGAGAGAAGCAGATGCGTGCTTTGAAA
TTAAAAATTCTCTCGTGCAGAAAAATTACAATATCCCGCTGGTTGCTGATATTCATTTTGCTCCACCTATTGCAA
TGCGGGTTGCTGAATGCTTTGACAAAATTCGTGTAAATCCAGGGAATTTCGCTGATAGGCGGGCACAGTTTGAGA
AGCTGGAGTACACAGAAGACGACTATCAGAAAGAACTTGAGCATATTGAGCAGGTATTTACACCACTGGTTGAAA
AATGTAAGAAATATGGAAGGGCTATCCGCATTGGGACAAACCATGGGAGCCTTTCAGATCGTATCATGAGCTACT
ATGGAGATTCTCCAAGGGGAATGGTTGAATCTGCATTTGAGTTTGCAAGGATATGCCGGAAACTGGATTTCCACA
ATTTTGTCTTTTCAATGAAAGCAAGTAACCCAGTTATCATGGTCCAGGCCTACCGTCTCCTTGTTGCAGAAATGT
ATGTCCAAGGCTGGGATTATCCATTGCACTTGGGTGTCACTGAAGCTGGAGAAGGTGAAGATGGGCGTATGAAAT
CTGCAATTGGCATTGGAACATTACTTCAGGATGGTTTGGGTGACACTATCAGGGTGTCCCTGACAGAATCCCCAG
AGATGGAGATAGACCCTTGCAGAAGATTGGCCAACCTTGGTATGAGAGCATCTGCGGCTCAACAAGGAGTGGCAC
CATTCGAAGAAAAGCACAGGCACTATTTTGACTTCCAGCGCCGATCTGGTGACTTGCCAGCTCAAAAAGAGGGTG
AAGAGGTTGATTATAGAGGCGTCTTGCACCGTGATGGCTCGGTTCTTATGTCAGTATCCTTGGATCAGTTGAAGG
CACCCGAACTTCTATACAAGTCTCTTGCTGCAAAGCTTGTAGTTGGAATGCCTTTCAAGGACCTGGCAACTGTTG
ACTCAATCTTATTGAGAGAACTTCCACCAGTGGATGATGTTGATGCTCGGCTAGCTCTAAAAAGGCTGATAGATA
TTAGCATGGGAGTTATTGCCCCCTTATCAGAGCAGCTAACAAAGCCATTACCGAACGCCATGGTTCTTGTAAATC
TGAAGGAGTTGGCCAGTGGCGCTCACAAGCTTCTGCCTGAAGGTACACGCTTGGTTGTGTCTTTGCGTGGCGATG
AGTCTTATGAAGAACTATATATCCTCAGAGACATTGATGCTACAATGATTCTACATGACCTTCCATTTTCAGAAG
AGAAAGTTGGGAGAGTTCAAGCGGCAAGGAGACTATTTGAGTTCCTAGCAGACAATTCCTTGAATTTCCCAGTAA
TTCACCACATTCATTTTCCAAAAGGGATTCACAGGGACGACTTGGTCATTGGTGCTGGTACAAATGTGGGGGCCC
TACTGGTAGATGGTCTGGGAGATGGTTTACTATTAGAAGCCCAAGACCAGGACTTTGACTTCCTGAGGAACACGT
CTTTCAACCTATTACAAGGTTGCAGAATGAGAAACACAAAGACGGAGTACGTCTCATGTCCATCATGTGGTAGAA
CTTTGTTTGACCTTCAAGAAATCAGTGCAGAAATCCGTGAAAAGACATCTCATTTGCCTGGCGTCTCGATTGCAA
TTATGGGTTGCATTGTGAACGGACCCGGTGAGATGGCTGATGCTGATTTCGGGTATGTTGGTGGTGCTCCAGGGA
AGATTGACCTTTATGTCGGAAAGACGGTGGTGAAGAGAGGAATAGCAATGGAGGGAGCCACTGATGCATTGATCG
AGCTGATTAAAGAGCACGGGCGTTGGGTAGACCCTCCTGCAGAGGAGTAAGATCGAACATTTATAGTTCGGTTCA
TATGAACGGAGAGGCTTTAAAGGGCTTGTAAAAAGACCAAATCTTTTACAGTAGAAGGGTGTAGATTTGTATCCC
ATTGATGTTATCTACAGTTAATAATACTAGAAATGGTAGCAATGTAGTTACTTGAGAATAAGAAATCCAAGATTT
CATGATGGCTTTACTTGGCG

>PB.6488.1 Euphorbia maculata HMBPP synthase Para.2 mRNA
GCGATTTTGTTTCCCTCAAATTCTTCACCAGCACACGCCCTTCTCTTCATTATCTCCTCTGCTTCCTCTCCAGAT
CCTTCATTCCCTTCACTTTCAGCTTTCGCTATCTATTCTCCCTTTGGTAGGTGAGAAATGGCGACTGGAGCTGTG
CCGGCGTCTTTTACTGGTCTGAAGACCAGGGAATCGAGCTTGGGGTTCGGAAAGAGCTTGGATTTTGTCAGAGTT
TGTGATTTGAAGAGGATCAAGTCCGGGAGGAAGAGGATTTCAATGATTCGTAACTCAAATGAGATGGTTGAGCTT
CAGCCTGCTTCAGAAGGAAGCCCTTTGTTAGTTCCTCGACAAAAGTATTGTGAATCTATTCACAAAACTGTGAGG
AGAAAAACCCGCACTGTAATGGTGGGAAACGTTCCTCTTGGTAGCGAGCATCCTATCAGGATCCAAACTATGACT
ACTACTGACACCAAAGATGTTGCTGGAACAGTTGAACAGGTGATGAGAATAGCAGATCAGGGGGCAGATATAGTT
CGGATAACAGTTCAAGGAAAGAGAGAAGCAGATGCTTGCTTTGAAATTAAAAATTCTCTCGTGCAGAAAAATTAC
AATATCCCGCTGGTTGCTGATATTCATTTTGCTCCACCTATTGCAATGCGGGTAGCAGAATGCTTTGACAAAATT
CGTGTAAATCCAGGGAATTTCGCTGATAGGCGGGCACAGTTTGAGAAGCTGGAGTACACAGAAGATGACTATCAG
AAAGAACTTGAGCATATTGAGCAGGTATTTACACCACTGGTTGAAAAATGTAAGAAATATGGAAGGGCTATCCGC
ATTGGGACAAACCATGGGAGCCTTTCAGATCGTATCATGAGCTACTATGGAGATTCTCCAAGGGGAATGGTTGAA
TCTGCATTTGAGTTTGCAAGGATATGCCGGAAACTGGATTTCCACAATTTTGTCTTTTCAATGAAAGCAAGTAAT
CCAGTTATCATGGTCCAGGCCTACCGTCTCCTTGTTGCAGAAATGTATGTCCAGGGCTGGGATTATCCATTGCAC
TTGGGTGTCACTGAAGCTGGGGAAGGTGAAGATGGGCGTATGAAATCTGCAATTGGCATTGGAACTTTACTTCAG
GATGGTTTGGGTGACACAATCAGGGTGTCCCTGACAGAACCCCCAGAGATGGAGATAGACCCTTGCAGAAGATTG
GCTAACCTTGGTATGAGAGCATCTGCTGCTCAACAAGGAGTGGCACCATTCGAAGAAAAGCACAGGCACTATTTT
GACTTCCAGCGCCGATCTGGTGACTTGCCAGCTCAAAAAGAGGGTGAAGAGGTTGATTACAGAGGCGTCTTGCAC

CGTGATGGCTCGGTTCTTATGTCAGTATCCTTGGATCAGTTGAAGGCACCCGAACTTCTATACAAGTCTCTTGCT
GCAAAGCTTGTAGTTGGAATGCCTTTCAAGGACCTGGCAACTGTTGACTCAATCTTATTGAGAGAACTTCCTCCA
GTGGACGATGTTGATGCTCGGCTAGCTCTAAAAAGGCTGATAGATATTAGCATGGGAGTTATTGCCCCCTTATCA
GAGCAGCTAACAAAGCCATTACCGAACGCCATGGTTCTTGTAAATCTGAAGGAGTTGGCCAGTGGCGCTCACAAG
CTTCTGCCTGAAGGTACACGCTTGGTTGTGTCTTTGCGTGGCGATGAGTCTTATGAAGAACTAGATATCCTCAAA
GACATTGATGCTACAATGATTCTACACGACCTACCATTTACAGAAGAGAAAGTTGGAAGAGTTCAAGCAGCAAGG
AGATTATTTGAGTTCCTAGCGGACAATTCTTTGAATTTCCCAGTAATTCACCATATTCATTTTCCAAATGGGATG
CACAGGGACGACTTGGTCATTGGAGCTGGTACAAATGTGGGGGCCCTACTAGTAGATGGTCTGGGAGATGGTTTA
CTATTAGAAGCCCCGGACCAGGACTTTGACTTCCTTAGGAACACCTCTTTCAACCTATTACAAGGTTGCAGAATG
AGAAACACAAAGACGGAGTACGTCTCATGCCCATCATGTGGTAGAACTTTGTTCGACCTTCAAGAAATCAGTGCA
GAAATCCGTGAAAAGACATCTCATTTGCCTGGCGTCTCGATTGCAATTATGGGTTGCATTGTGAATGGACCCGGC
GAGATGGCTGATGCTGATTTCGGGTATGTCGGTGGTGCTCCAGGAAAGATTGACCTTTACGTCGGAAAGACGGTG
GTAAAGAGAGGAATAGCAATGGAGGGAGCCACTGATGCATTGATCGAGCTGATTAAAGAGCACGGGCGTTGGGTA
GACCCTCCTGCAGAGGAGTAAGATCGAACATTTCGGTTTATATGAAAGGAGAGGCTCTAGAGGGGTTGTTAAGAA
GACCAAATCTTTTATCTTACCTTGTAGATTTGTATCCCATTAAAATTATCTACAGTTGATATACTAGAAATGATA
GCAATGTTGTTTCTTGAGAAATAAGAAATCCAAGATTTCATGATGTCTTGGCGAATCAAGCGGTGTAGTTTTATT
ATTTAACGAAAATTGTACTCACAACATTACATTTTTCGGATTTGGTTGGTTGTACAGTCAATGCTAGTTATTTTG
AATAATAATTTATGCATTTAGCTT

>PB.12927.1 Euphorbia maculata IPP/DMAPP synthase Para.1 mRNA
ATCTATTCAGCTGAGTCCACTAGTTTTTCCCCTCCGCAGAATTGACCACCGTCTCTTGTCTTCTCCGGCGAGCCA
TGGCCATTTCTCTCCAACTCTGCCGCCTCTCTCTCCGCGCCGATATATTTTCCGGCGACAAACTCGTCCCTCATT
TCCCCCGTAGATCGATCTCCATCCGATGCTCCGCCGGCGACTCCGCTTTGCCGTCGGTCTCCGGTGACTCCGATT
TCGACGCCAAGGTCTTCCGGCACAACTTGACTCGGAGCAAGAATTACAATCGGAGAGGATTTGGACACAAGGAGG
AGACCTTGGAGCTCATGAGTCAGGAATATACGAGTGATATTATCAAGACTTTGAGGGAAAACGGGAATGAATACA
CTTGGGGAAATGTTACTGTGAAATTGGCGGAAGCTTATGGGTTTTGCTGGGGCGTTGAGCGAGCTGTGCAGATTG
CTTACGAAGCTAGAAAGCAATTTCCGGAGGATAAGATTTGGATTACTAATGAAGTTATTCATAATCCAACTGTAA
ACAAGCGTTTGGAAGAGATGGAAGTTGTAACTATTCCTGTTGCAGAAGGGGGAAAGCAATTTGAAGTTGTGAACA
AAGGAGATGTTGTGATTTTGCCTGCTTTTGGAGCTGCAGTTGATGAGATGCTGACTCTAAGTCAAAAGGATGTTC
AAATTGTCGATACTACTTGCCCTTGGGTATCTAAGGTCTGGAATACGGTTGAGAAGCACAAGAAGGGAGACTATA
CTTCAATAATTCATGGTAAATATAGTCACGAGGAAACTATTGCTACTGCTTCTTTTGCGGGAAAGTACATCATAG
TGAAGAATATGGATGAGGCGATGTACGTTTGTGATTACATTCTTGGTGGTCAACTTAATGGCTCTAGCTCAACAA
GAGAAGCGTTCCTTAAGAAATTTGAAAAAGCAGTTTCCAAGGGATTTGATCCAGATGTTGACCTTGTCAAAGTCG
GTATTGCAAATCAAACTACAATGCTCAAGGGAGAAACAGAAGCCATTGGAAAATTGGCAGAGAAGACCATGATGC
AGAAATATGGTGTCGAAAATGTCAATGATCACTTTATTAGCTTCAACACCATTTGTGATGCTACTCAAGAGAGGC
AAGATGCAATGTATAAATTGGTAGAGGAAAAGCTGGATCTGATTCTGGTTGTTGGTGGATGGAACTCCAGCAACA
CTTCACACCTCCAAGAAATTGCAGAAGAGCGTGGAATTCCTTCATATTGGATCGACTATGAAAAGAGAATCGGTC
CAGGAAACAAAATCGCTTATAAGCTGAATCATGGGGAATTAGTTGAGAAAGCGAACTTTCTCCCAGAAGGTCCCA
TTACAATCGGTGTAACATCCGGTGCCTCAACTCCAGATAAGGTTGTTGAAGATGCGCTTGTGAAAATATTTGAAA
TCAAACGTCAAGAATTGTTGCAATTGGCATAGATTATGATTCTGGTAACAGACCCCGAGCATGAAATTAACATAA
GGTGCTGAAGATCTGTGTTGTACCATCAGAATGTACAAAAGGAAGAGCAATAATGTTGATAATATGCTTTGTATT
TGAGCTTATCTTATGTAAGAACTAATGTAAAATCCCAAATAAGGGTAGAGTCCGAAATTCATTGTACTAGTAAAC
CATCTCTTGTGTTGGTGTTGCATTTTTTGGTTAGAAAATTTCATATACACACCTTTACTATCG

>PB.12928.1 Euphorbia maculata IPP/DMAPP synthase Para.2 mRNA
ACAATAAAAACCCAAAGCCATAATCACAAAAAGGATCTCTTCAGCTGAGTCCACTGGTTTTGTTTCCCCCTCCGC
CCATTTGACCACCGTCTCTTGTCTTCTCCGGCGAGCCATGGCCATTTCTCTCCAACTCTGCCGCCTCTCCCTCCG
CGCCGACCTCTTTTCCGGCGACAAGCGCGTCCCTTGTTTCCCCCGTAAATCGATCTCCATCCGCTGCTCCGCCGG
CGACTCCGCTTCGCCGTCGGTCTCCGGTGACTCCGATTTTGATGCTAAGGTATTCCGGCACAACTTGACTCGGAG
CAAGAATTACAACCGGAGAGGATTCGGACACAAGGAGGAGACCTTGGAGCTCATGAATCAGGAATATACGAGTGA
TATTATCAAGACTTTGAAGGAAAACGGGAACGAATACACTTGGGGAAATGTTACTGTGAAATTGGCTGAAGCTTA
TGGGTTTTGCTGGGGCGTTGAACGCGCTGTGCAGATTGCTTATGAAGCTAGAAAGCAATTTCCTGAAGATAAGAT
TTGGATTACTAATGAAGTTATCCATAATCCGACTGTTAATCAGCGTTTGGAGGAGATGGAAGTTGTAACTATCCC
TGTTGCGGAAGGGGGAAAGCAATTTGAAGTTGTGAACAAAGGAGATGTTGTTATTTTGCCTGCTTTTGGAGCTGC
GGTAGACGAGATGTTGACTCTAAGTCAAAAGGATGTTCAAATTGTCGATACTACTTGCCCTTGGGTATCTAAGGT
CTGGAATACGGTTGAGAAGCACAAGAAGGGAGACTACACTTCAATAATTCATGGTAAATACAGTCACGAGGAAAC

TATTGCTACTGCTTCTTTTGCAGGAAAGTACATCATAGTGAAGAATATGGATGAGGCGATGTATGTTTGTGATTA
CATTCTTGGTGGTCAACTTAATGGTTCTAGCTCAACAAGAGAAGCTTTCCTTAAGAAATTTGAAAAAGCAGTTTC
CAAGGGATTTGATCCCGATGTTGACCTTGTCAAAGTCGGTATTGCAAATCAAACTACAATGCTCAAGGGAGAAAC
AGAAGCCATCGGAAAATTGGCGGAGAAGACCATGATGCAGAAATATGGTGTCGAAAATGTCAACGATCACTTTAT
TAGCTTCAACACCATCTGTGATGCTACTCAAGAGAGGCAAGATGCTATGTATAAATTGGTAGAAGACAAGCTGGA
TCTGATTCTAGTTGTTGGTGGATGGAACTCAAGCAACACTTCACACCTCCAAGAAATTGCAGAAGAGCGTGGAAT
TCCTTCATATTGGGTTGACTCTGAAAAGAGAATCGGTCCAGGAAACAAAATCGCTTATAAATTGAATCACGGGGA
ATTGGTTGAGAAAGAGAACTTCCTGCCAGAAGGCCCCATTACAATCGGTGTAACATCCGGTGCCTCAACTCCAGA
CAAGGTTGTTGAAGATGCGCTTGTGAAAATATTCGAAATCAAACGTCAAGAATTGTTGCAATTGGCATAAATGCG
TAAAATTAACAGAAGATTCTTAAGATCTGTGTTGTACCATCAGAATGTACAAAAGGAAGAACAATAATGCCGATA
ATGTGTTTGTTATTTCAGCTTATCTTGTCTTAGAACTAATGTAAAATCCCGAATAAGGTAGAGTCCGAAATTCAG
AGTACTAGCAAACAATCTCATGAATTGGTGTTGCATTCTTTGGTTAAAAAATTTCATATATATGCCTTTACTGT

Terpenoid synthase pathway genes

>PB.13712.1 Euphorbia maculata IDI Para.1_Iso.1 mRNA
GCGGCATGAAACATAATGCTGAGCCTCTAAGCTATTAGCTGCATAAATCGGCCACCGCCGCCGCAAATATTTACG
GCGAAAGAGTAGAAGAGAGACGAAGCTAGCCATCCACAGCTGAGAGTAATTGAATCTCCGCCTGAACTAGCGGTT
TCGATCAATATGGAGTCGAATTCGAATTCCATCGCTGTTCCCGAAGCCGACAGCACTCTCCTTTCTATCTGCTAC
ACCCGCGGCTCTCTTCAGCTCCTCGATCAGAGAAAGCTTCCTTTGGAGACTGTATATCTGGATATCCACGATTCA
TCAGATGGATGGTCTGCAATACGAGAGATGGTTGTTCGTGGAGCACCTGCAATTGCTATTGCTGCTGCTCTTTCT
CTTGCAGTGGAAGTGTTTAACTTGAAGGATTTCAATGGAACTTGTAATGATGCTACCTCTTTCCTCTTTAACAAA
TTAGAGTACCTTGTTTCCAGCCGACCAACTGCTGTGAATCTCTCGGATGCTGCAACAAAACTTAGGGAGATTATC
TTACAAGCCGCTGCTACTGCTTCAGAGTCAAAATCTATTTTCCAGGCCTACATAGAAGCTGCAGAAATTATGCTC
AAAGACGACGTTGCTTCAAATAAGGCAATCGGATCACATGGAGCTAGTTTTCTTCAGAGCTTGCTGAAGAACTCC
AAGAGGCTATCAGTTCTGACTCATTGCAACACTGGAAGTCTAGCTACAGCTGGATTTGGCACAGCCTTAGGTGTC
ATTCGAGCACTCCACTCTAACAGTGTCTTAGAGAGAGCCTATTGCACGGAAACTCGCCCATTCAATCAGGGATCA
AGGCTTACAGCTTTTGAATTGGTGCACGAAAAGATTCCTGCTACTCTTATAGCTGATTCTGCTGCAGCTGCGTTA
ATGAAAGATTGTCGAGTGAGTGCTGTAATTGTTGGAGCAGATCGAGTTGCTGCAAACGGAGATACAGCTAACAAG
ATCGGGACTTATAGCCTAGCGCTATGTGCCTCCTATCACAACATCCCGTTCTTTGTTGCAGCTCCATTGACCTCG
GTTGACTTATCTCTTTCTTCTGGAAAAGAAATTATAATAGAGGAAAGATCACCGAAAGAGTTATTGAATTCTCGT
GGAGGACTAGGAGAGCAAGTTGCAGCATCTGGGATCGATGTCTGGAATCCGGCTTTTGACGTTACTCCTGCACAA
CTGATAACCGGTATCATCACTGAAAAGGGTGTCATCACTAAGACGGGTAGCGATGTTTTTGACATAAAGAATTTT
GTAAAAGAGACAACTGGACTATCAGGGACATGAGCTGTGGACCGAATGACATGTCGTTTTTTCTGATATGATCAT
ATTGAAGGGCTATAGCCTACTGTCATGTTAAAACGAATCTCGTATGTATTGTGGCCCTCTTATAAACCAAGCCAA
ACCTTCAAGGCGATGTTTCTGATAGTTAATTTATTCAAGCTGAATAAAAAGGTTTCTCGTTTGACATGTAATTTT
TGTAAACCTTTGTTTGCACAAAATGTTACGGATCCCATACTCTACTCTCAGCTCTAAACTCTATTTTGTTAACTT
GATATAGTGAATTCACTGCAATTT

>PB.13712.2 Euphorbia maculata IDI Para.1_Iso.2 mRNA
GCCGCAAATATTTACGGCGAAAGAGTAGAAGAGAGACGAAGCTAGCCATCCACAGCTGAGAGTAATTGAATCTCC
GCCTGAACTAGCGGTTTCGATCAATATGGAGTCGAATTCGAATTCCATCGCTGTTCCCGAAGCCGACAGCACTCT
CCTTTCTATCTGCTACACCCGCGGCTCTCTTCAGCTCCTCGATCAGAGAAAGCTTCCTTTGGAGACTGTATATCT
GGATATCCACGATTCATCAGATGGATGGTCTGCAATACGAGAGATGGTTGTTCGTGGAGCACCTGCAATTGCTAT
TGCTGCTGCTCTTTCTCTTGCAGTGGAAGTGTTTAACTTGAAGGATTTCAATGGAACTTGTAATGATGCTACCTC
TTTCCTCTTTAACAAATTAGAGTACCTTGTTTCCAGCCGACCAACTGCTGTGAATCTCTCGGATGCTGCAACAAA
ACTTAGGGAGATTATCTTACAAGCCGCTGCTACTGCTTCAGAGTCAAAATCTATTTTCCAGGCCTACATAGAAGC
TGCAGAAATTATGCTCAAAGACGACGTTGCTTCAAATAAGGCAATCGGATCACATGGAGCTAGTTTTCTTCAGAG
CTTGCTGAAGAACTCCAAGAGGCTATCAGTTCTGACTCATTGCAACACTGGAAGTCTAGCTACAGCTGGATTTGG
CACAGCCTTAGGTGTCATTCGAGCACTCCACTCTAACAGTGTCTTAGAGAGAGCCTATTGCACGGAAACTCGCCC
ATTCAATCAGGGATCAAGGCTTACAGCTTTTGAATTGGTGCACGAAAAGATTCCTGCTACTCTTATAGCTGATTC
TGCTGCAGCTGCGTTAATGAAAGATTGTCGAGTGAGTGCTGTAATTGTTGGAGCAGATCGAGTTGCTGCAAACGG
AGATACAGCTAACAAGATCGGGACTTATAGCCTAGCGCTATGTGCCTCCTATCACAACATCCCGTTCTTTGTTGC
AGCTCCATTGACCTCGGTTGACTTATCTCTTTCTTCTGGAAAAGAAATTATAATAGAGGAAAGATCACCGAAAGA
GTTATTGAATTCTCGTGGAGGACTAGGAGAGCAAGTTGCAGCATCTGGGATCGATGTCTGGAATCCGGCTTTTGA
CGTTACTCCTGCACAACTGATAACCGGTATCATCACTGAAAAGGGTGTCATCACTAAGACGGGTAGCGATGTTTT
TGACATAAAGAATTTTGTAAAAGAGACAACTGGACTATCAGGGACATGAGCTGTGGACCGAATGACATGTCGTTT
TTTCTGATATGATCATATTGAAGGGCTATAGCCTACTGTCATGTTAAAACGAATCTCGTATGTATTGTGGCCCTC
TTATAAACCAAGCCAAACCTTCAAGGCGATGTTTCTGATAGTTAATTTATTCAAGCTGAATAAAAAGGTTTCTCG
TTTGACATG

>PB.13713.1 Euphorbia maculata IDI Para.2 mRNA
GCGGCATGAATCATAATGCCGAGCCTGTGCTGCTTTAATCCGCCGCCGCGAATATTTACCGGCTAAAGAGTGGAA
GAGAGACGAAGCTCGCCATCCACAGCTGACTAGAGTAGGTTTCGATCAATATGGAGTCCAATTCGAACTCCATCG
CTGTTCCTGCAGCCGATAACACTCTCCTTTCTATCTGCTACACCCGCGGCTCTCTTCAGCTCCTCGATCAGAGGA
AGCTTCCTTTGGAGACTGTGTATCTGGATATCCGTGATTCATCAGATGGATGGTCTGCAATCAGAGAGATGGTTG
TCCGTGGAGCACCTGCAATTGCTATTGCAGCTGCTCTTTCTCTTGCAGTGGAAGTGTTTAACTTGAAGGATTTCA
ATGGAAGTTGTAATGATGCTGCTTCTTTCCTCTTTAACAAACTGGAGTACCTTGTTTCCAGCCGACCAACTGCTG
TGAATCTCTCTGATGCTGCAAAAAAACTTAAGGAGATTATCTTGCAAGCTGCTGCTACTGCTTCTGAGTCAAAAG

CTGTTTTTCAGGCCTACATAGAAGCCGCAGAAATTATGCTCAAAGATGACGTTGCTTCAAATAAGGCAATTGGAT
CATATGGAGCTAGTTTTCTTCAAAGCTTACTAAAGAACTCCAAGAGGCTATCAGTTCTGACTCATTGCAACACTG
GAAGTCTAGCTACAGCAGGATATGGTACTGCCCTTGGGGTTATTCGAGCACTCCATTCTCAAGGTGTCTTAGAGA
GAGCCTACTGCACTGAAACTCGCCCATTTAATCAGGGATCAAGGCTCACAGCTTTCGAATTGGTGCACGAAAAGA
TTCCTGCTACTCTGATAGCAGATTCTGCTGCAGCTGCATTGATGAAAGACGGACGAGTGAGTGCTGTAATTGTCG
GAGCAGATAGAGTAGCCGCAAATGGTGATACAGCTAACAAGATTGGGACTTATAGCCTTGCGCTATGCGCATCCT
ATCTCAACATCCCGTTCTTTGTTGCAGCTCCACTGACCTCCATTGATTTGTCTCTTTCTTCTGGAAAAGAAATTG
TAATCGAGGAAAGATCGCCGAAAGAGTTATTGAATTCTCGGGGAGGTCTAGGAGAGCAAGTTGCAGCATCTGGGA
TCGATGTCTGGAATCCGGCTTTTGATGTTACTCCCGCACGATTGATAACCGGCATCATCACCGAAAAGGGTGTCA
TCACCAAGACGGGTAGCGAAGTTTTCGACATAAAGAATTTTGTAAAAGAGACAGCTGGACTACCTGAGAAATGAA
CTGCTGACCGAATGCCATGTCGTTTTTTTCTGAGATGATCATATTTAAGGGCTATTAGCCTCATGTCACGATGAA
ACGAATCTCGTATGTATTGTGGCTATCTTATAAACCAAGCCAAACCTTCGAGGGTAATGTTTGTTTCTGTACTAG
TTAATTTATCGAAGCTGAATAAAAAGGTTTCTCGTTTGACATGTAATTTTTGTAAACCTATGTTTGCACAAGAAC
TTACCGATCTGTGTTATTAATTTTCTT

>PB.19277.1 Euphorbia maculata GPP synthase Para.1 mRNA
GTCACTACATTTATAACAACCATTTCATTTGATTTCAATTTCCCTTCTTTGCTATTAAACTCGGGGTCGGTTATA
TGCTGTACTTTTGTAGATTTCCCCTTTTGATTCTTCTTCTTCATTTCGCTTCTTTTGAGTAGAAACAAACGCTGG
CGGCAATGTTCTTCTCGCGGGGCTTATCACGTATTTCGAGAAGCGGCGGCCGTTGGTTTCTTTCTCGGCGGATAC
ACCCTTGTGTTCTCTCGAACGATTCCAATCTAATTGGAGATTCTACACAAAAGGTTTTTAATCGCAGAGAAACTT
GTATTTGGAGCTTGCCTGCTTTGCATGGCTTCAGACAACACATTCACCAGACCAGCCCCTTAGTCGAGGAACAAT
CTGACCCATTTTCGCTAGTTGCTGATGAATTATCTCTCCTCGCTAATAGGTTACGGGAAATGGTGGTTGCCGAGG
TCCCGAAGCTTGCCTCTGCCGCTGAGTACTTCTTCAAGATGGGAGTAGAAGGAAAGAGGTTTCGTCCTACGGTTT
TGTTGCTGATGGCATCAGCTTTGAATGTGCACGTTCCTAGTACATTGCCAGCGAACACTGAAGAAGCTTTGGAAA
CTCAATTAAGATCTAGACAGCAGCGTATAGCTGAAATTACAGAAATGATACATGTGGCGAGTCTTCTCCATGATG
ATGTATTGGATGATGCAGACACAAGGCGTGGCATTGGTTCATTAAATTTTGTCATGGGAAATAAGGTAGCAGTTT
TAGCCGGAGATTTTCTGCTATCAAGAGCTTGTGTGGCACTTGCTTCTTTGAAGAATACAGAGGTTGTATCACTTT
TAGCGACAGTGGTGGAGCATCTTGTAACAGGCGAAACCATGCAGATGACTAGCACATCTGAGCAACGTTGTAGCA
TGGAGTACTATATGCTAAAGACGTACTATAAAACAGCATCTTTGATATCAAACAGCTGCAAAGCAATTGCGCTTC
TTGCTGGGCAGACAGCAGAAGTTTCGATTTTGGCCTTCGAGTATGGAAAAAATCTGGGATTAGCGTTTCAATTGA
TAGATGACGTCCTTGATTTCACGGGCACATCAACTTCCCTGGGAAAGGGTTCATTATCCGACATTCGCCATGGGA
TCGTGACAGCTCCAATATTGTTTGCAATGGAAGAGTTCCCACAATTACGTGCAGTTGTTGAGGAGGGTTTTGACA
AACCCGAGAATGTTGATATTGCCTTAGAGTACCTTGGAAAGAGCCGAGGAATACAAAGGACCCGAGAGCTAGCTG
CAAAGCATGCTAAATTTGCTGCAGAAGCAATTGAATCACTGCCTGAATCCGATGATGAAGATGTAATAAAGTCGA
GGCGAGCATTGGTTGACCTTACTCACCGAGTAATCTCAAGAAACAAATGATATGTTTTTGAACTTCCACCTGCTC
TCGATAGTAGGTTTTTTTTTGTTGTTTCGAATAGGGTGTGTGGTTTTTCTTTATATGTGGTAGCCGGGTCATTGG
TTCCATTATTTGTTGTTTAACAAAACTGTTCACAGACGATCCAATTGTACATTGTAGAAAGAAAACGAAATGGTA
GTTACATGTTTTTGTTTC

>PB.19278.1 Euphorbia maculata GPP synthase Para.2 mRNA
AAGAGAGAAAGAGTTGTATTTCACAAGTCTCAGTGGTTCTCAGCTCCAATGGTAGCAGATCATTTTTCTCTGGAA
ATCAAGAAAAACAAGTGCATAGATTGAGGAATTCGAGCTAGGGATTTGTTTCCCTGCTTGCATGTCAGCTCTTCG
GCTTTAATCGAGGGAAAATCAGTGTTTTAAGCTCGGAGAGATTTTGAAGGATGATTATGAAAAGAAGAATATCAG
CTTAGGAAGGGGCATGCGTTTTCAAAATGATGGCTGATTCTGCTTCAGAGCCTGGTTTTCAATCTAATAAGTCGG
GCTCAAGATATATAGGCAACTCCTTCTTTAACATTCCTGGTTTGTTTGTAGGATTTGGTTTTAAAGGCTCATCAG
ATTCTGAGTCTATTAAAAGTCCATCATCTCCTCTTGATTTCAGCCTTTTCTCAAATCTTAGCAGCCAATTTAGTC
TCAAGAACCCAAAATCACCAACCCAGAATAAGTGGCATTCCAGTAAAGTGGGACTTGGAATCATAAACTTACTTG
TTGATGAGCCCAAATCAACTGGTCTTGTTCTAAGTTCATCAAAGAGGAAAAATGTAATCTTTGGATCAGATGTGT
GGACCGGATACTCTGAGCAGTCGAAGTCTTTTCCAGCAGATTACATGGTCTTGCTACTGTCACAAAGCGAAACTC
CTAAATCAAGTTCGAATTCATCAATTTCTGATGCTCTTTCTGGAAAATCAGGAGTTCCTTTGGAACCTGAATCTT
TTCAAAACTCTTCTTCAGCTAGCTTATCCCCCAAATCCCATTTGAGTTCGAAAAAGTTTTGTTCAGAGAATCGAA
CTACTACTATGTCAAGTTCATCCCTTTTTAGTAACAGTTCTTTGGATGTCAAAACGAGTTCACTTCCTGTACCAA
TAGGCTCTCTCTCTGCTAGTGAGATTGAGCTTTCTGAGGATTATACCTGTATAATTTCTTATGGTCCAAACCCGA
AAACAACTCATATTTTCGGTGACTGTATTTTGGAATGTCACACAAACGACTTACCAAATCTTAACAACACAGAAA
ACCTGAGTTCTGATTCCTTGGACAACTCAACTCCATTTCCATCTGATGAGTTTTTGAGCTTCTGCAACTCTTGTA

AGAAAAAACTGGAGGAAGGAGATGACATTTATATATATAGAGGAGAAAAAGCATTTTGTAGCTCCGATTGTCGTT
CAGAGGACATTTTTGCTGAGGATGAAATAGGGAAAAGTTGCTGTGATCCCCAGAATAGCTCTCCTGCATCAAGCT
ACCATGAAGACGTTTTCTTAACGGGTATGGGGCTTTGACTTGGTGATGGATCTGGATCCGGATTAGCAACAACGT
GGAGCTATTTTTCGATACCCTATATTTTTGTTGTGTTAGTTGAGCCATAAAAATACTTTTTTTTTTTTTTTGAA
TCTGTGTTGGATTATATGGGTTAGAAGTGGCCAATATGAGCCCTCACCCATCAATTTTTCTGTTATAATGAGCAT
TTATTATGTAGCTATTACCTTTTGTGACTGTTAAAACTCATTATCAAAGCTTGGCGTTTTGTCATCTTTGATTAC
TCAATTGATCATGTTTTTGCT

>PB.13017.1 Euphorbia maculata Monoterpene synthase Para.1_Iso.1 mRNA
GTCTACAATCAGACTAATTTAACAAAATTCCCTTCCCTACTCTTCTAGTCTCTTCTTCACCTTGATTTCTTTTGC
TCTCTCCGTCTTCACCATGAGCTGGTGGTGGGCCGGCGCTATCGGCGCCGCTAAGAAGAAATTCGACGAAGATGA
CGCTCCTAGAGCCTACCAGAGCGTGGCTCTCGTGCTCGGAGTCACCGGGATCGTCGGCAACAGTTTGGCCGAGAT
CCTCCCCCTCAACGACACCCCCGGCGGCCCCTGGAAAGTCTACGGCGTCGCCCGCCGCCCCCGCCCTAGCTGGAA
CGCCGACCATCCGGTGGAGTACATCCAGTGCGATATCTCAGATCAGGAGGAATCGGAGTCCAAATTATCCAAGCT
CACCGACGTGACTCACATCTTCTACGTCACCTGGACGAATCGCTCGTCGGAGGCCGAGAATTGCGAGATCAACGG
CGCAATGTTCCGCAACGTCCTCGCCGCCGTGGTACCTAACGCGCCGAATCTCCGCCACATCTGCCTCCAAACCGG
CACAAAGCACTATCTCGGTCCGTTCGAGTTGTTCGGCAAGATCCAGCCGCACGATCCGCCGTTCGCGGAGGATCT
GCCGCGGTTAAACGCGCCGAATTTCTACTACACGCTAGAGGACATCCTCTTCGATTTCGTCGCGAAGAAGGAAGG
ATTGACCTGGTCGATCCACCGGCCCGACGTGATCTTCGGATTCTCGCCGTACAGTCTGATGAACGTGATCGGAAC
TCTCTGCGTCTACGCCGCGATTTGCAAACACGAAGGGCTCCCGCTGCGGTTTCCGGGGGCGAAATCGGCGTGGAA
CTGTTACGCGATCGCCTCCGACGCCGATCTGATAGCTGAGCACCAGATCTGGGCGGCGGTGGATCCGTACGCGAA
GAACGAGGCGTTCAACTGCCACAACGGCGACGTGTTCAAGTGGAAGCACTTGTGGAGAGTTCTGGCAGAGCAATT
TGGGATCGAGGAGAACGGAGTTGAGGAGGGGGAGGGGGGATTGAGCTTGGTGGAGACAATGAAGGGGAAAGAGGC
GGTTTGGGAGGAAATTGTGAGGGAAAATCAGCTGCGGGAGACGAAATTGGATGAGGTGGCGCAGTGGTGGTTTGT
GGATTTGATTTTGGGCGGAGAGCCGGTGATTTCGAGCATGAACAAGAGCAAGGAGCATGGGTTTTTGGGGTTCAG
GAATTCGAAGAATTCGTTCGTGTCGTGGATTGATAAGATGAAGGGGTATAAGATTGTGCCTTCGTTTGGTGTGAA
TTGAGTTGATCGTTGGTGTTGCGCTGGTGTTGGTGGTGAATAAGGTCTGTTGGTTTGATTTGTATGTGTACTGGC
CTTTTGCGTTTTGCAGTTTCTGCTTATTTGTTTTGTTGCTCCAAATAATCATTGAATTGTTCAAAGGGATAACGA
TTTTTTAAGTTTTTTTGGTTTGTGGAACATGAGGATTTCATTGACATTGAAATGTTTTACCAGTTAATCACTTCA
GATGCAAACATAGGAGACTGAAATCCTTGCTCGTTTTATTTGTTGAATCACTCTCAGCCAAAATGGTGGTCTGGC
CACAGTTCATTAATGTTCATTTCCAGCAATTTTAGAGTTTTCTCAGGGTATTTCCTTGGGATAATAGGAACTTGT
AATATTCAACCATCGATATATATATAAAAATAAGGAGGAAACTCTTTTCCAATTTACAAGTG

>PB.13017.2 Euphorbia maculata Monoterpene synthase Para.1_Iso.2 mRNA
TCTACAATCAGACTAATTTAACAAAATTCCCTTCCCTACTCTTCTAGTCTCTTCTTCACCTTGATTTCTTTTGCT
CTCTCCGTCTTCACCATGAGCTGGTGGTGGGCCGGCGCTATCGGCGCCGCTAAGAAGAAATTCGACGAAGATGAC
GCTCCTAGAGCCTACCAGAGCGTGGCTCTCGTGCTCGGAGTCACCGGGATCGTCGGCAACAGTTTGGCCGAGATC
CTCCCCCTCAACGACACCCCCGGCGGCCCCTGGAAAGTCTACGGCGTCGCCCGCCGCCCCCGCCCTAGCTGGAAC
GCCGACCATCCGGTGGAGTACATCCAGTGCGATATCTCAGATCAGGAGGAATCGGAGTCCAAATTATCCAAGCTC
ACCGACGTGACTCACATCTTCTACGTCACCTGGACGAATCGCTCGTCGGAGGCCGAGAATTGCGAGATCAACGGC
GCAATGTTCCGCAACGTCCTCGCCGCCGTGGTACCTAACGCGCCGAATCTCCGCCACATCTGCCTCCAAACCGGC
ACAAAGCACTATCTCGGTCCGTTCGAGTTGTTCGGCAAGATCCAGCCGCACGATCCGCCGTTCGCGGAGGATCTG
CCGCGGTTAAACGCGCCGAATTTCTACTACACGCTAGAGGACATCCTCTTCGATTTCGTCGCGAAGAAGGAAGGA
TTGACCTGGTCGATCCACCGGCCCGACGTGATCTTCGGATTCTCGCCGTACAGTCTGATGAACGTGATCGGAACT
CTCTGCGTCTACGCCGCGATTTGCAAACACGAAGGGCTCCCGCTGCGGTTTCCGGGGGCGAAATCGGCGTGGAAC
TGTTACGCGATCGCCTCCGACGCCGATCTGATAGCTGAGCACCAGATCTGGGCGGCGGTGGATCCGTACGCGAAG
AACGAGGCGTTCAACTGCCACAACGGCGACGTGTTCAAGTGGAAGCACTTGTGGAGAGTTCTGGCAGAGCAATTT
GGGATCGAGGAGAACGGAGTTGAGGAGGGGGAGGGGGGATTGAGCTTGGTGGAGACAATGAAGGGGAAAGAGGCG
GTTTGGGAGGAAATTGTGAGGGAAAATCAGCTGCGGGAGACGAAATTGGATGAGGTGGCGCAGTGGTGGTTTGTG
GATTTGATTTTGGGCGGAGAGCCGGTGATTTCGAGCATGAACAAGAGCAAGGAGCATGGGTTTTTGGGGTTCAGG
AATTCGAAGAATTCGTTCGTGTCGTGGATTGATAAGATGAAGGGGTATAAGATTGTGCCTTCGTTTGGTGTGAAT
TGAGTTGATCGTTGGTGTTGCGCTGGTGTTGGTGGTGAATAAGGTCTGTTGGTTTGATTTGTATGTGTACTGGCC
TTTTGCGTTTTGCAGTTTCTGCTTATTTGTTTTGTTGCTCCAAATAATCATTGAATTGTTCAAAGGGATAACGAT
TTTTTAAGTTTTTTTGGTTTGTGGAACATGAGGATTTCATTGACATTGAAATGTTTTACCAGTTAATCA

>PB.13018.1 Euphorbia maculata Monoterpene synthase Para.2_Iso.1 mRNA

GTCGACAATCACGCCACTCTAATTTTTAATTTCCTCTCCTACTCTTCTAGTCTCTTCTTCGCCTTGTTTTTTTGC
TCTCTCCGTCTTCACCATGAGCTGGTGGTGGGCCGGCGCTATCGGCGCCGCTAAGAAATTCGATGAAGACGACGC
TCCCAGAAGCTACCAGAGCGTGGCTCTCGTGCTCGGAGTCACCGGAATCGTCGGCAACAGTTTGGCCGAGATACT
CCCCCTCAACGACACCCCCGGCGGCCCCTGGAAGGTCTACGGCGTGGCCCGCCGCCCCCGCCCCAGCTGGAACGC
CGACCATCCGGTGGAGTACATCCAGTGCGATATCTCCGATCAGGAGGAATCGCAATCGAAGCTATCCAAGCTCAC
CGATGTGACTCACATCTTCTACGTCACCTGGACGAATCGGTCGTCGGAGGCCGAGAACTGCGAGATCAACGGCGC
GATGTTCCGCAACGTCCTCGCCGCCGTCGTACCGAACGCGCCGAATCTCCGCCACATCTGCCTCCAAACCGGCAC
CAAGCACTACCTCGGCCCGTTCGAGCTGTACGGCAAGATCCAGCCGCACGATCCGCCGTTCGCGGAGGATCTGCC
GCGGCTAAACGCGCCGAATTTCTACTACACGCTAGAGGACATCCTCTTCGAGTTCGTCGCGAAGAAGGAAGGATT
GACCTGGTCGATCCACCGGCCCGACGTGATATTCGGATTCTCGCCGTACAGCCTGATGAACGCGATCGGAACTCT
CTGCGTCTACGCCGCGATATGCAAGCACGAAGGACTCCCGCTGCGGTTTCCGGGCACGAAATCGGCGTGGAACAG
TTACGCGATTGCCTCGGACGCCGATCTCATAGCGGAGCACCAGATCTGGGCAGCGGTGGACCCGTACGCGAAGAA
CGAGGCGTTTAACTGCCACAACGGCGACGTTTTCAAGTGGAAACATTTCTGGAGAGTTTTGGCGGAGCAATTTGG
GATAGAAGAGTACGGATTTGAAGAGGGGGAGGGGAGATTGAGCTTGGCGGAGACGATGAAGGGGAAAGAGGCGGT
TTGGGAGGAAATTGTGAGGGAGAATCAGCTGCAGGAGACGAAATTGGAGGAGGTGGCGCAGTGGTGGTTTGTGGA
TTTGATTTTGGGGGGAGAGCCGGTGATTTCGAGCATGAACAAGAGCAAGGAGCATGGGTTCTTGGGGTTCAGGAA
TTCGAGGAATTCGTTTGTTTCATGGATTGATAAGATGAAGGGGTATAAGATTGTGCCTTCTTTTGGTTTGAATTG
AATGGATACATTGGTGAGTCTGTCTGTTGGTTTGATTTGTATCTGTACTGGCCTTTTGTGTTGTATTGTGTTTCT
GCTTATTTGTTTTGCTGTTCCAAATAATCTGGTCTTCTGTTGGGGTAATCAGTCATTGAATTGTTTAAACAGATA
ATGATTTTAAAGGTTTTTTGGTTTGTGT

>PB.13018.2 Euphorbia maculata Monoterpene synthase Para.2_Iso.2 mRNA
TCGACAATCACGCCACTCTAATTTTTAATTTCCTCTCCTACTCTTCTAGTCTCTTCTTCGCCTTGTTTTTTTGCT
CTCTCCGTCTTCACCATGAGCTGGTGGTGGGCCGGCGCTATCGGCGCCGCTAAGAAGAAATTCGATGAAGACGAC
GCTCCCAGAAGCTACCAGAGCGTGGCTCTCGTGCTCGGAGTCACCGGAATCGTCGGCAACAGTTTGGCCGAGATA
CTCCCCCTCAACGACACCCCCGGCGGCCCCTGGAAGGTCTACGGCGTGGCCCGCCGCCCCCGCCCCAGCTGGAAC
GCCGACCATCCGGTGGAGTACATCCAGTGCGATATCTCCGATCAGGAGGAATCGCAATCGAAGCTATCCAAGCTC
ACCGATGTGACTCACATCTTCTACGTCACCTGGACGAATCGGTCGTCGGAGGCCGAGAACTGCGAGATCAACGGC
GCGATGTTCCGCAACGTCCTCGCCGCCGTCGTACCGAACGCGCCGAATCTCCGCCACATCTGCCTCCAAACCGGC
ACCAAGCACTACCTCGGCCCGTTCGAGCTGTACGGCAAGATCCAGCCGCACGATCCGCCGTTCGCGGAGGATCTG
CCGCGGCTAAACGCGCCGAATTTCTACTACACGCTAGAGGACATCCTCTTCGAGTTCGTCGCGAAGAAGGAAGGA
TTGACCTGGTCGATCCACCGGCCCGACGTGATATTCGGATTCTCGCCGTACAGCCTGATGAACGCGATCGGAACT
CTCTGCGTCTACGCCGCGATATGCAAGCACGAAGGACTCCCGCTGCGGTTTCCGGGCACGAAATCGGCGTGGAAC
AGTTACGCGATTGCCTCGGACGCCGATCTCATAGCGGAGCACCAGATCTGGGCAGCGGTGGACCCGTACGCGAAG
AACGAGGCGTTTAACTGCCACAACGGCGACGTTTTCAAGTGGAAACATTTCTGGAGAGTTTTGGCGGAGCAATTT
GGGATAGAAGAGTACGGATTTGAAGAGGGGGAGGGGAGATTGAGCTTGGCGGAGACGATGAAGGGGAAAGAGGCG
GTTTGGGAGGAAATTGTGAGGGAGAATCAGCTGCAGGAGACGAAATTGGAGGAGGTGGCGCAGTGGTGGTTTGTG
GATTTGATTTTGGGGGGAGAGCCGGTGATTTCGAGCATGAACAAGAGCAAGGAGCATGGGTTCTTGGGGTTCAGG
AATTCGAGGAATTCGTTTGTTTCATGGATTGATAAGATGAAGGGGTATAAGATTGTGCCTTCTTTTGGTTTGAAT
TGAATGGATACATTGGTGAGTCTGTCTGTTGG

>PB.19208.1 Euphorbia maculata FPP synthase mRNA
GGCTGAGCTTCGCCGTCGTCGAAGCATTAAAATGACTTGAAAACAATCTCTACCCGTTTTCCTCTTCTTTGCTAT
TAATCCCCAAAATGAGTCCGCGTCGGTTGTATGTTGTTCTTTTGTGATTTTCCCTTTTTTGACTTCTTCATCTTC
CTCTTCATTTCGCTTCTCTTTAGCGGCAGCGGCAGTATAAACTGACACTAGCGGCGATGTTATTCTCGCGGGGTT
TATCGCGTATTACGCGAAGCGGCAGCCGTTGGTTTCTCTGTCAGCGAATAAAGCCTTCTATTGTTTCATATAATC
CCCATTTGATCGGAAATACTACACAACAGTAGGTTTTAAATAGCAGAGAAACTTATATTGGGAGCTCACCTGCTT
TGCATGGCTTCAGACAATACATTCATCAGACCAGCTCTTATATTGAGGAACAGTCTGAGCCATTTTCTCTGGTTG
CTGATGAACTATCACTCCTTGCTAATAGGTTGCGGGAAATGGTGGTTACTGAGGTCCCTAAGCTCGCCTCTGCTG
CCGAGTATTTCTTCAAGATGGGAGTAGAAGGAAAGAGGTTTCGCCCAACGGTGTTGTTGCTGATGGCATCAGCTT
TGAATGTAGGCATACCTGGTACGTTGCCAACTGCTACTGAAGAGAGTTTGGAAACACAACTAAGAACAAGACAGC
AGCGTATAGCTGAAATTACAGAAATGATACATGTGGCGAGTCTTCTCCATGATGATGTGTTGGATGATGCAGACA
CTAGGCGTGGAATTGGTTCATTAAATTTTGTGATGGGGAATAAGGTTGCAGTATTAGCCGGAGATTTTCTGCTAT
CAAGAGCTTGTGTGGCACTAGCTTCTTTAAAGAACACAGAGGTGGTCTCACTTTTAGCAACAGTGGTAGAACATC
TTGTAACCGGCGAAACTATGCAGATGACTAGTACATGTGAGCAACGTTGTAGCATGGAGTACTATATGCAGAAGA
CATACTACAAGACAGCATCGTTGATTTCAAACAGCTGCAAAGCAATAGCGCTTCTTGCTGGGCAAACAGCTGAAG

TTTCCATTTTGGCTTATGAGTATGGCAAAAATCTGGGATTGGCATTTCAGTTGATAGACGACGTCCTTGATTTCA
CGGGCACATCAACGTCCCTTGGAAAGGGTTCACTGTCTGACATTCGCCATGGGATCGTGACAGCTCCAATATTGT
TTGCAATGGAGGAGTTCCCACAGTTGCGTGCAGTAGTTGAGGAGGGCTTTGATAATTCCAGGAATATTGATATTG
CCTTAGAGTACCTCGGGAAAAGCCGAGGAATACAGAGGACTCGAGAGCTAGCTGCAAAGCATGCTAGCCTTGCTG
CAGAAGCCATCGATTCACTGCCCGAATCCGATGATGAAGAAGTAAGGAAGTCGAGACGGGCATTGATAGACCTTA
CTCACAGAGTGATCACAAGAAATAAATGAAAGATTTTGGAATTCTCGACACCTTTTTACAATAGATTTTCAGTTT
CGAACTTTTTGTTTATTGATTTCATTACGTTAATTGTTGTTCAATGATGATGTTGCTCGTGGACATTCCATTTGT
AAATTACAGGTCATGTGGAGATGGTGAAAGAGGGTAACAAAATAATGATATTTACAGGTCATTTAAGCCATTGAT
TTGAAGTG

>PB.6478.1 Euphorbia maculata Sesquiterpene synthase mRNA
AGTGTCCAAAGATGTCACTCTTTTCCCCAATTTTTCTGTATAAATAAAGACTCCATTTTTGCCCCAATTCCATCA
CTCTTCTTCTTCTACTTCTTTAGCCGCCATGGCTGCCTCTTCTCACTTCTTCAGTTCTTCCATTTCAGCGAAATC
AGGACAGCATCCTAAGTCACAATTAGTATCAGAAAGCAAGACAGCAATTCAGCTTGATGAAACTAAAGATAAGAT
TAAAAAGATGTTCAACAAGATTGAACTTTCGGTTTCGTCATATGATACTGCTTGGGTAGCTATGGTCCCCTCTCC
AAATTCACTAGACGTTCCTTTTTTCCCTGAATGCTCGAAATGGATTGTCGATAATCAATGCAAAGACGGCTCTTG
GAGTGTTCATCATCATCACAATCCATCCTTAGTTAAAGATTCTCTATCGTCTACGTTAGCTTGTGTTCTTGCATT
GAAGCAATGGGGCATTGGCGAAAATCAAGTAAACAAAGGACTCCGGTTCATTGAGCTGAATTCGGGTTTGTTGAA
TGATCAGAAGCAAAAGTCACCTATTGGATTTGACATAACATTTCCCGGTATGCTCGAGCGTGCTAAAGAGTTGGG
TTTGAATCTTCCTTTGGATTCAAAATACGTCGAATCGATGCTTCTCAGGAGAGATTTGGATCTTAAAAGCCGCTG
TGGCAGCACAACAGAGGGGAGAAAGGCTTACATAGCATATATTTCCGAAGGGATCGGGAAATTTCAAGATTGGAA
CATGGCCATGAAATATCAAAGGAAAAACGGGTCGCTTTTCAACTCACCGTCAGCAACTGCAATGGCTTTTAGTCA
TCTTCGTGATGCTAGTTGCCTTCGGTACCTTCGGTGTGCTTTAAAAAAGTTTGGAAATTCAGTTCCTACCGTTTA
TCCCTTCGATGTTTTTGTCCGACTAAGTATGGTTGACACCCTTGAAAGTTTGGGAATCGGACGGCTTTTTGGGGA
TGAGATAAAATCGGTTCTTGATGATACTTACATACGATGGTTGCAAGGAAATGAGGAGATATTTCTAGATTGTAC
AACTTGTGCTCTGGCATTTCGGGTATTACGAGCTAACGGCTTCAATGTCTCTTCAGAAAAATTGAATCGATTCAC
CAAAGAATACTTTAATAATTCACTTGAAGGATATTTGGAGGACGTAAAACCGGCTTTGGAGTTATATAAAGCTTC
GCAAGTACTCTATCCGGACGAAATATTTCTGGAAAAGCAAATTTCTTGGACAGGTCAGTTCTTGCAGGAGAAATT
ATCTACCGGTGTAAGACAAAGTGATGGAATTGATAAACACATTGTTGAAGAGGTTCATGATGCTCTCAATTTCGC
TTCTTATGCGGATTTGGAACGGTTAACTAACTGGAGGAGAATTACAAAATATAGAATTGATGAAACAAAGATGCT
AAAATCATCGTATCGTTGCTCAAACGTTGCAAATCCATATTTCATTAAACTGGCTGTAGAAGATTTCAACTTCTG
CCAATCAATGCACCGGGAAGAACTCCAACATCTCGGGAGATGGATTGTGGAGAAAAGATTGGACAAGCTGAAGTT
TGCTAGGCAAAAGCTTGGCTATTGTTACTTTTCTTGTGCAGCTTCTCTCTTTGCACCCGAAATGACTGATGCTCG
TTTATCGTGGGCGAAAAATGCGGTGCTTACAACTGTTGTGGATGATTTCTTTGATGTTGGGGGTTCAGAGGAGGA
ATCGGTCAACCTCATTGAATTAACCGAGAGGTGGGATGTTGATGGGAACTCTCGGTTTTGTTCCGAGAATGTCGA
GATCATATTTTCTGCGCTTCATAGTACCATTTGTGAGATTGGAGAGAAAGCATTTGGGTATCAAGGGCGTAACGT
GACCCGTCATATTATCAAAATTTGGTTGGATTTGCTGAAATCAATGTTGACCGAAGCTCGATGGTCAAAAAGCAA
AGCTACGCCAGCTCTCGATGAATATATGGCCAATGGATACACATCATTTGCTCTAGGCCCGATTGTCCTGCCCGC
TCTTTTCTTTGTCGGGCCAAAACTAACCGAGAAACATTTTGCGGGCCCCGAATTGCATGAATTGTTTAAGACAAT
GAGCACTTGTGGACGACTACTCAACGACTGGAGAAGTTTTAAGAGGGAATTTGAACAAGGGAAGCTTAATGCTGT
CTCACTACACATGATTAATGGCTCGATAACCGAAGAAGAAGCTGTTAGAATAATAAAGGGTTTGATTGAAAGCAA
CCGAAAAGAAGTGTTGAGGCTCGTTTTGCAGGAAAAGGATAGTGAAATACCGAGACGATGCAAGGAGTTGTTCTG
GAAAATGACCAAAGTAGTAAACATGTTTTACTCACAAAATGATGGTTTTACTTCTAATGAGATGATTAATACTGC
AAATGCACTCATTAATCAACCTGTATCTCTATAAACCGATCTGTTAGAGTTTGACAATATACTCGGTAACTCGGG
TTGGAACCTGAGTGCCTCATCGATTGTGAAAAGAAACTTGTTCAGTGAGTTGTTTAGAAGAAATTAGGTAGATTT
TAAGTACACTTATTTTGGATGAAATTAAGGGATTTTATGTGTGGGTCAATCCATGGTTTTTAGATCCAGACCAGC
CATCAAATCGGTGTAGAAACAAGTCCATGATTTTAAGATTTAATCAAATTAATTTAGTGATTG

>PB.14297.1 Euphorbia maculata GGPP synthase Para.1 mRNA
CTTCTTCTTCTTCTTCGAAGCCCCATTTATGGCCGGAGCTTGAAGACGGCAATTTATCGATCACATTCCCAATTT
CAACCCTCAATTTCATCAATTCCCCCCTAATTAAAGATGAATTCGATGAATTTCAATTCATGGGTTCACCCCTCT
TCATTCTGTAACCAATTATCCAGATCCAAATCCCAATCCCCGCCATTAAAAAGCTTCCCAATTCGCCATTGCAAT
CCCAAATCAAAGCAACCCATTCCTTCAATTTCTCCAATTTCCGCAATCATGACCAAAGAAGAAGAAACCCTCCAA
AAGCCCTCTTTCGATTTCAACTCCTACATGGTCCAGAAGGCCGCCGCCGTCCACCGCGCCCTCGACGCCGCCGTG
CCCCTCAAGGAGCCGGCCAAAATCCACGAGTCCATGCGCTACTCCCTCCTCGCCGGCGGCAAGAGGGTCCGCCCG
GCGCTCTGCCTCGCCGCCTGCGAGCTCGTCGGCGGGGACGAGCCCGCGGCGATGCCCGCCGCCTGCGCGGTGGAG

ATGATCCACACCATGTCCCTCATCCACGACGACCTCCCCTGTATGGACAACGACGACCTCCGCCGCGGGAAGCCC
ACCAACCACATCGTCTTCGGGGAGGACGTGGCGGTGCTCGCCGGCGACGCCCTCCTCTCCTTCGCCTTCGAGCAC
ATCGCTACCGCCACCCGCAACGTCTCGCCCGAGAGGATCGTCCGGGCGATCGGGGAGCTGGCTAAGGCGATAGGC
GCGGAAGGACTGGTCGCGGGCCAGGTAGTGGATTTAAGCTCGGAGAGGGCATCCGAGGTGAAATTGGAGACATTA
GAGTTCATCCACGTGCACAAGACGGCGAAACTGCTCGAGGCCTCGGTGGTTTTGGGTGCGATTTTGGGCGGAGGG
AGCGACGAGGAGGTGGAGAAGTTGAGGAAGTACGCAAGGGGGATCGGATTGTTGTTTCAAGTGGTGGATGATATA
TTGGATGTGACGAAATCGTCGGAGGAGTTGGGGAAAACCGCAGGAAAAGATTTGGTGGCGGATAAAGTTACATAT
CCGAAGCTAATGGGGATCGAGAAATCGAGGGAGTTTGCGGAGGAGTTGAGGCGGGAGGCCGAGGCGCAGTTGGGG
TGCTTTGACACCAAGAAGGCTGCGCCTTTGGTCGCCTTGGCGAATTATATTGCGTATAGGCAGAATTGATTGGTT
GTATTGAAGTTTAGTGATACATTTTGTGTTGTGAATGTTTTGTGTTGAATTTTAGTGATACATGTTGTTGGTTGG
TAGC

>PB.14298.1 Euphorbia maculata GGPP synthase Para.2 mRNA
ATTTAAGCTCTTTGCTCTTTCTCCGCGTTACCCAACTCCATTTCCGCCGTAAAGTCGCTGCCTTTATCACTTTCC
CCGGCGGTCTTTTATGGCCGGAGCTTGAAGACGGCAATTTTCCGATCACATTCCCAATTCTCACTCTCAATTTCA
TCTATTTCCCCCCGATTAAAGATGAATTCGATGAATTTAGGTTCATGGGTTCACCCCTCTTCAATCTGTCATCAA
TTATCCAGATCCAAATCCAAATCTCCGCCATTGAAAAGCTTCCCAATTCGCCATTACAATCCCAAATCAAAGCAA
CCCGTTTCTCCAATTTCCGCAATTATGACGAAGGAAGAAGAAACCCTCCAAAAGCCCTCTTTCGATTTCAACTCC
TACATGGTCCAGAAGGCCGCCGCCGTCAACCGGGCCCTCGACGCCGCCGTGCCCCTCAAAGAGCCGGCCAAAATC
CACGAATCGATGCGGTACTCCCTCCTCGCCGGCGGCAAGAGGGTCCGGCCGGCGCTCTGCCTCGCCGCCTGCGAG
CTCGTCGGCGGGGACGAATCCGCCGCGATGCCCGCCGCCTGCGCGGTGGAGATGATCCACACGATGTCCCTCATC
CACGACGACCTCCCCTGTATGGACAACGACGACCTCCGCCGCGGAAAACCCACCAACCACATCGTCTTCGGCGAG
GACGTGGCGGTGCTCGCCGGCGACGCCCTCCTCTCCTTCGCCTTCGAGCACATCGCCGCCGCCACCCAAAACGTT
TCGCCCGAGAGAATCGTCCGGGCGATTGGGGAGCTAGCTAAGGCGATAGGCGCTGAAGGATTGGTCGCGGGCCAA
GTGGTTGACATAACGTCGGAGAGAGCATCGGAGGTGAAATTGGAGACCCTAGAGTTCATCCACGTCCACAAGACG
GCGAAATTGCTGGAGGCGGCGGTGGTTTTGGGGGCGATTTTGGGCGGAGGGACCGATGAGGAGGTGGAGAAGTTG
AGGAAATATGCGAGGGGGATCGGGTTGTTGTTTCAAGTGGTGGATGATATATTGGATGTGACGAAATCGTCGGAG
GAGTTGGGGAAAACGGCGGGGAAAGATTTGGTGGCAGATAAAGTGACGTATCCGAAGCTGTTGGGGATCGAGAAA
TCGAGGGAATTCGCTGAGGCGCTGAAAAAGGAGGCTGAGGCGCAGTTGGGGTGCTTTGATGCTGAAAAGGCGGCG
CCTTTGGTCGCCTTGGCCAGTTACATTGCTTATAGGCAGAATTGATTGATTTGGCGGGGCTCTTTGGTGTTGAAT
TTTAGTGATACATTTAGTTGATAGCATTTGGTGGGATTAGTTTTGTGGTAATGAGATTTGTGTTGGATTTGATTG
AAAATTGCTTCTAAGGATTGAAATTTCTAATGTAGTCCCGGATGATTGAGGCAAGGTAAAGTTCTTGAGATGTTT
TGATCTTGTTTAATATTTGTATATACGAGTTAAAAGATATTGATCTTTATCGATCG

>PB.18527.1 Euphorbia maculata Ent-Kaurene synthase mRNA
ACCACTCTTCTTCTTCTTCGTCTTCTTAGGCCGCCATGGCTGCCTCTTCTCACTTCTTCAGTTCCCCCATTTCAG
CGAAATCGAGACAACATCCTAAGTCACCATTAGTAGCAGAAAGCAAGACAGCAGTTCAGCTTGATGAAACTAAAG
ATAAGATTAAAAACATGTTCAACAAGATTGAACTTTCGGTTTCGGCATATGATACTGCTTGGGTAGCAATGGTCC
CCTCTCCAAATTCACTAGACGTTCCTTTTTTTCCCGAGTGCTCGAAATGGATTGTCGATAATCAAAGAAAAGACG
GGTCTTGGAGTGTTCATCATTGTCATGATCCATCATTAGTTAAAGACTCTCTATTGTCTACGTTAGCATGTGTTC
TTGCATTGAAGCAATGGGGCATTGGCGAAAATCAAGTAAACAAAGGACTCCGGTTCATTGAGCTGAATTCGGGTT
TGTTGAGTGATCAGAAGCAAAAGACACCTATTGGATTTGACATAACATTTCCCGGTATGCTCGAGCGTGCTAAAG
AGTTGGGTTTGAATCTTCCTCTGGATTCCAAATACGTCGAGTCAATGCTTCTCAGGAGACACTTGGGTCTTAAAA
GTGGCTGTGGCAGCATCACAGATGGAAGAAAAGCTTACTTAGCATATATTTCCGAAGGGATCGGGAATTTCCAAG
ACTGGAATATGGCCATGAAATATCAAAGGAAAAACGGGTCGCTTTTCAACTCGCCATCAGCAACTGCAACTGCTT
TTAGTCATCTTCATGATGCTAGTTGTCTTCGGTACCTTCGGGGTGCTTTAAAAATGTTTGGAAATTCAGTTCCTA
CCGTTTATCCCTTCGATATTTTTGCCCGACTAAGTATGGTTGACACCCTCGAAAGTTTGGGAATTGGGCGGTTTT
TCCAGGACGATATAAAATCGGTTCTCGATGATACTTATAGACAATGGTTGCAGGGAAATGAAGAGATATTTCTGG
ATTGTACAACTTGTGCTATGGCATTTCAGGTATTACGAACTAACGGCTACAACGTCTCTTCAGAGAAATTGAATT
TATTTACCAAGGAGCACTTTAATAATTCACTTGAAGGATATTTAGAGGACATTAGGCCTGCTTTGGAGTTATATA
AAGCGTCACAAGTACTTTATCGAGACGAAATATTTCTGGAAAAACAAATTTCATGGACAAGTCATTTCTTGAAGG
AGAAATCGTCTGTGGGTTTAAGACAAAGTGATGGAATTGATAAACACATTGATGATGAGGTTCATGATGCACTCA
ATTTCGCTTCTTATGCCGATTTGGAACGGTTAACTAACTGGAGGAGAATTGCAAAATACAGAGTTGACGAAACAA
AGATGCTAAAAACATCATATCGTTGCTCAAACGTTGCGAATCAACATTTCATTAAACTGGCAGTAGAAGATTTCA
ACTTCTGCCAATCAATGCACCAGGAAGAACTCCAATTTCTCGGAAGATGGGTAGTGGAGAAAAGATTGGACAAGC
TGAAGTTTGCCAGGCAAAAGCTTGGCTATTGTTACTTTTCGTGTGCAGCTTCTCTCTTTGCACCCGAAATGACCG

ATGCTCGTTTATCGTGGGCGAAAAATGCCGTTCTTACAACTGTTGTGGATGATTTCTTTGATGTTGGAGGTTCAG
AGGAGGAATTGGTCAACCTCATTGAATTAATCGAGAGGTGGGATGTTGATGGGAACTCTCGGTTTTTCTCTGAGA
ATGTCGAGATCATATTTTCTGCACTTCATAGCTCCATTTGTGAGATCGGAGAGAGAGCACTCGGGTATCAAGGGC
GTAACGTGACCGGTCATGTTATCAAAATTTGGTTGGATTTGTTGAAATCGATGTTGACCGAAGCTCGATGGTCAA
AAAGAAAAGCTACGCCGACTCTCAACGAATATATGGCTAATGGATACACATCATTTGCTCTCGGACCAATTGTCC
TGCCCGCTCTTTTCTTTGTTGGGCCAAAACTGACCGAGAAAGATTTTGCAAGCCCCGAATTGCACGAGTTGTTCA
AGACCATGAGCACTTGTGGACGTCTACTCAACGACTGGAGAAGCTTTAAGAGGGAATTTGAACAAGGGAAGCTTA
ATGCTGTCACGCTGCACATGATTAATGGCGATATGACCGAAGAAGAAGCTGTTGGAAAAGTAAAGGGTTTGATCG
AAAGCAACAGGAAAGAAGTGTTGAGGCTCGTTTTGCGGGAGAACGATAGTGAAATACCGAGACGGTGCAAGGACT
TGTTTTGGAAAATGACCAAAGTAGTGAGCATGTTTTACTCACAAGATGATGGTTTTACCTCTAATGAGATGATTA
ATACTGCAAATGCACTCATTAATCAACCCATATCCTTATAAACTGATCTGTTAGAGTTTGACAAGATACTCGGGG
ACTCGGGTTTGAACACAAGTGCCTGATCGATTGTAAAAAGAAACTTGCATGTAAAAGTTATACAAAAGCAAAACA
AATGATCCTTTGC

>PB.2914.1 Euphorbia maculata Triterpene synthase para.1_Iso.1 mRNA
GTGCTAACCAAACGTGTATCTCTGCTTCTCGCTCTCTCGTCTGTCACTTTCAAGCTCTCCAAGAACTCAGTGGAG
TGAACAGATCAGAATGTGGAGGTTGAAGATCGCCGAGGGAGCTGAGAATCCATGGCTGCGGAGTGTTAACAATCA
TGTTGGAAGACAGGTTTGGGAGTTTGATCCTAATCTTGGATCTCCTGAAGATCACTTGCAGATCGAGAATGCTCG
TCGGAGTTATCGCGAGAATCGGTTTAGTATGAAGCATAGTTCAGATCTATTGATGAGGATTCAGTTTGCTAAGGA
GAATCCGTTGAGTGAAGTTTTGCCTCAAGTTAAGCTGAATGATGACGATGATATCACTGAAGAGGCTGTCTCTGT
TACTCTGAGGAGAGCTCTGGATTATTATTCGACTATTCAGGCACATGATGGCCATTGGCCTGGAGATTATGGAGG
CCCTATGTTTCTTATGCCTGGATTGGTCATAGCTCTATACGTTACTGGAGCACTCAACGCTGTTTTATCAGAGGA
GCATAAGAAAGAGATGTGCCGATACCTATACAACCACCAGAATAGAGATGGTGGATGGGGCTTGCATATTGAGGG
CCCGAGCACGATGTTTGGGAGTGTTTTGTCCTATGTTACTTTGAGATTGTTGGGTGAAGGGGCTAATGATGGAGA
TGGAGCTATGGAGAGAGGGCGTAAATGGATCCTGGACCATGGCAGTGCTACTGCAATCACATCATGGGGGAAAAT
GTGGCTTTCAGTGCTTGGAGCTTTCGAGTGGTCTGGGAATAATCCCCTCCCTCCCGAGATATGGCTTCTCCCATA
TATACTCCCATTCCATCCCGGGAGGATGTGGTGCCACTGCCGGATGGTCTATCTGCCCATGTCGTATTTATTTGG
AAAAAGGTTTGTTGGCCCAATTACATCAACAGTTTTGTCTTTGAGAAAGGAGCTATTCACTGTCCCGTATCATGA
AGTAGACTGGAATCATGCACGCAACCAATGTGCAAAGTGAGCTCCTGTCCACAGCTTCACAGCTACAACTTCCAG
AGAAGACCTGTATTATCCTCATCCCATGGTGCAAGATGTACTTTGGGCAACTCTTGACAAGTTAGTTGAGCCCAT
TCTAATGAGTTGGCCTGGAAAAAAGTTGAGAGAAAAGGCTCTTCAGACTGTGATGCAGCACATACATTATGAGGA
TGAAAACACTCGCTATATATGCATTGGTCCTGTAAACAAGGTGTTAAATATGCTCTGCTGTTGGGTGGAAGATCC
AAATTCTGAAGCGTTCAAGCTGCATCTTCCAAGAATACATGATTACCTTTGGCTAGGTGAAGATGGAATGAAAAT
GCAGGGTTATAATGGGAGTCAACTGTGGGATACAGCTTTTGCAGTTCAGGCAATCTTGTCTACTAATCTTGCTCA
CGAATACGGTCCAACTTTAAAAAAGGCTCATGCTTATATTAAAAGTTCTCAGGTCTTGGATGATTGTCAAGGAGA
TCTTGATTTTTGGTATCGTCACATCTCAAAAGGTGCATGGCCTTTTTCAACTGCAGATCATGGTTGGCCAATATC
AGATTGCACAGCAGAGGGATTGAAAGCTGCTCTGTTGTTATCCAAAGTTCCACGTGATATTGTCGGGGATTCATT
AGATGTTAACCAGTTATGTGATTCAGTCAATGTCATCCTCTCTCTGCAGAATGGTGATGGTGGATTTGCTACATA
TGAGCTTACAAGATCTTACAGTTGGTTAGAGTTAATCAATCCTGCTGAAACTTTTGGTGACATCGTCATCGACTA
TCCTTATGTCGAGTGTACTTCAGCAGCAGTTCAAGCTCTCACAGCTTTTAGAAAATTACATCCCGAACATCGACG
GGACGAAATAGAATCTTGCATTGAAAAGGCAATCAAGTTTATTGAGAAAATTCAGGCAGCAGATGGCTCATGGTA
TGGCTCATGGGGTGTTTGCTTCACCTATGGTACATGGTTTGGCGTTAAAGGGCTGGTGGCTGCTGGAAAGAACTT
TAACAATTGCCCCAGTATTCGAAAGGCTTGTGAGTTTCTGCTGTCTAAACAGTGTCCTTCTGGTGGTTGGGGAGA
GAGTTATCTTTCGTGTCAAACAAAGGTTTATTCCAATCTTGACGATAACAGGTCTCATGTTGTAAATACTTCTTG
GGCTATGCTGAGCCTTATTGATGCTGGGCAGGCTGAGAGAGACCCAACGCCATTGCACCGTGCAGCAAGGTACCT
GATAAATTCTCAAATGGAAAATGGAGATTTCCCTCAGCAGGAAATCATGGGAGTGTTCAACAAGAACTGCATGAT
AACATATGCAGCGTACAGAGATATATTCCCAATATGGGCATTGGGAGAGTATCGAACCCGGGTACTGCAGCAGTC
TTCGTAAGAAAAACACCAAAAATTTTAATATATTTATCGATTTTAGGACTCAAATTAGTTTCTTTCATTTACCGG
AAGATTTAAATAATCTAATTGCAGGCTTGTTAGATTTATGTTTAGATTAATGCAAACTTGAACAGCACCTTTTCT
ATAGTATCATTGTTATTGATTTGTTCTTCTGTGTCATGTACGCTTCAAAGTTACAACTCTTCGGGACGAAATGAA
AACGCAAGTTTATATGTG

>PB.2914.2 Euphorbia maculata Triterpene synthase para.1_Iso.2 mRNA
AACCAAACGTGTATCTCTGCTTCTCGCTCTCTCGTCTGTCACTTTCAAGCTCTCCAAGAACTCTGGAGTGAACAG
ATCAGAATGTGGAGGTTGAAGATCGCCGAGGGAGCTGAGAATCCATGGCTGCGGAGTGTTAACAATCATGTTGGA
AGACAGGTTTGGGAGTTTGATCCTAATCTTGGATCTCCTGAAGATCACTTGCAGATCGAGAATGCTCGTCGGAGT

TATCGCGAGAATCGGTTTAGTATGAAGCATAGTTCAGATCTATTGATGAGGATTCAGTTTGCTAAGGAGAATCCG
TTGAGTGAAGTTTTGCCTCAAGTTAAGCTGAATGATGACGATGATATCACTGAAGAGGCTGTCTCTGTTACTCTG
AGGAGAGCTCTGGATTATTATTCGACTATTCAGGCACATGATGGCCATTGGCCTGGAGATTATGGAGGCCCTATG
TTTCTTATGCCTGGATTGGTCATAGCTCTATACGTTACTGGAGCACTCAACGCTGTTTTATCAGAGGAGCATAAG
AAAGAGATGTGCCGATACCTATACAACCACCAGAATAGAGATGGTGGATGGGGCTTGCATATTGAGGGCCCGAGC
ACGATGTTTGGGAGTGTTTTGTCCTATGTTACTTTGAGATTGTTGGGTGAAGGGGCTAATGATGGAGATGGAGCT
ATGGAGAGAGGGCGTAAATGGATCCTGGACCATGGCAGTGCTACTGCAATCACATCATGGGGGAAAATGTGGCTT
TCAGTGCTTGGAGCTTTCGAGTGGTCTGGGAATAATCCCCTCCCTCCCGAGATATGGCTTCTCCCATATATACTC
CCATTCCATCCCGGGAGGATGTGGTGCCACTGCCGGATGGTCTATCTGCCCATGTCGTATTTATTTGGAAAAAGG
TTTGTTGGCCCAATTACATCAACAGTTTTGTCTTTGAGAAAGGAGCTATTCACTGTCCCGTATCATGAAGTAGAC
TGGAATCATGCACGCAACCAATGTGCAAAGGAAGACCTGTATTATCCTCATCCCATGGTGCAAGATGTACTTTGG
GCAACTCTTGACAAGTTAGTTGAGCCCATTCTAATGAGTTGGCCTGGAAAAAAGTTGAGAGAAAAGGCTCTTCAG
ACTGTGATGCAGCACATACATTATGAGGATGAAAACACTCGCTATATATGCATTGGTCCTGTAAACAAGGTGTTA
AATATGCTCTGCTGTTGGGTGGAAGATCCAAATTCTGAAGCGTTCAAGCTGCATCTTCCAAGAATACATGATTAC
CTTTGGCTAGGTGAAGATGGAATGAAAATGCAGGGTTATAATGGGAGTCAACTGTGGGATACAGCTTTTGCAGTT
CAGGCAATCTTGTCTACTAATCTTGCTCACGAATACGGTCCAACTTTAAAAAAGGCTCATGCTTATATTAAAAGT
TCTCAGGTCTTGGATGATTGTCAAGGAGATCTTGATTTTTGGTATCGTCACATCTCAAAAGGTGCATGGCCTTTT
TCAACTGCAGATCATGGTTGGCCAATATCAGATTGCACAGCAGAGGGATTGAAAGCTGCTCTGTTGTTATCCAAA
GTTCCACGTGATATTGTCGGGGATTCATTAGATGTTAACCAGTTATGTGATTCAGTCAATGTCATCCTCTCTCTG
CAGAATGGTGATGGTGGATTTGCTACATATGAGCTTACAAGATCTTACAGTTGGTTAGAGTTAATCAATCCTGCT
GAAACTTTTGGTGACATCGTCATCGACTATCCTTATGTCGAGTGTACTTCAGCAGCAGTTCAAGCTCTCACAGCT
TTTAGAAAATTACATCCCGAACATCGACGGGACGAAATAGAATCTTGCATTGAAAAGGCAATCAAGTTTATTGAG
AAAATTCAGGCAGCAGATGGCTCATGGTATGGCTCATGGGGTGTTTGCTTCACCTATGGTACATGGTTTGGCGTT
AAAGGGCTGGTGGCTGCTGGAAAGAACTTTAACAATTGCCCCAGTATTCGAAAGGCTTGTGAGTTTCTGCTGTCT
AAACAGTGTCCTTCTGGTGGTTGGGGAGAGAGTTATCTTTCGTGTCAAACAAAGGTTTATTCCAATCTTGACGAT
AACAGGTCTCATGTTGTAAATACTTCTTGGGCTATGCTGAGCCTTATTGATGCTGGGCAGGCTGAGAGAGACCCA
ACGCCATTGCACCGTGCAGCAAGGTACCTGATAAATTCTCAAATGGAAAATGGAGATTTCCCTCAGCAGGAAATC
ATGGGAGTGTTCAACAAGAACTGCATGATAACATATGCAGCGTACAGAGATATATTCCCAATATGGGCATTGGGA
GAGTATCGAACCCGGGTACTGCAGCAGTCTTCGTAAGAAAAACACCAAAAATTTTAATATATTTATCGATTTTAG
GACTCAAATTAGTTTCTTTCATTTACCGGAAGATTTAAATAATCTAATTGCAGGCTTGTTAGATTTATGTTTAGA
TTAATGCAAACTTGAACAGCACCTTTTCTATAGTATCATTGTTATTGATTT

>PB.2914.3 Euphorbia maculata Triterpene synthase para.1_Iso.3 mRNA
AACCAAACGTGTATCTCTGCTTCTCGCTCTCTCGTCTGTCACTTTCAAGCTCTCAAGGAACTCGGGAAAGTGAAC
GGATCAGAATGTGGAGGTTGAAAATCGCCGAGGGAGCTGAGAATCCATGGCTGCGGAGTGTTAACAATCATGTTG
GAAGGCAGGTTTGGGAGTTTGATCCTAATCTTGGATCTCCTGAAGATCATTTGCAGATCGAGAATGCTCGTCAGA
GTTTTCGCGAGAATCGGTTGAGTATGAAGCATAGCTCAGATCTATTGATGAGGATTCAGTTTGCGAAGGAGAATC
CGTTGAGTGAAGTTTTGCCGCAAGTTATGCTGAATGACGACGATGCTATCACTGAAGAGGCTGTCTCTGTTACTC
TGAGGAGAGCTCTGGATTATTATTCGACTATTCAGGCACATGATGGTCATTGGCCTGGAGATTATGGAGGCCCTA
TGTTTTCTTATGCCTGGATTGGTCATAGCTCTATACGTTACTGGAGCACTCAATGCTGTTTTATCAGAGGAGCAT
AAGAAAGAGATGTGTCGATACCTATACAACCACCAGAATAGAGATGGTGGATGGGGCTTGCATATTGAGGGCCCG
AGCACGATGTTTGGGAGTGTTTTGTCCTATGTTACTTTGAGGTTGTTGGGTGAAGGGGCTAATGACGGAGACGGA
GCTATGGAGAGAGGGCGTAAATGGATCCTGGACCATGGCAGTGCTACTGCAATCACATCATGGGGGAAAATGTGG
CTTTCAGTGCTTGGAGCTTTCGAGTGGTCTGGGAATAATCCCCTCCCTCCCGAGATATGGCTTCTCCCTTATATA
CTCCCATTCCATCCTGGGAGGATGTGGTGCCACTGCCGGATGGTCTATCTGCCCATGTCATATTTATTTGGAAAA
AGGTTTGTTGGCCCAATTACATCAACAGTTTTGTCTTTGAGAAAGGAGCTATTCACTGTCCCATATCATGAAGTA
GACTGGAATCATGCACGCAACCAATGTGCAAAGGAAGACCTGTATTATCCTCATCCCATGGTGCAAGATGTACTT
TGGGCAACTCTTGACAAGTTAGTTGAGCCCATTCTAATGAGTTGGCCTGGAAAAAAGTTGAGAGAAAAGGCTCTT
CAGACTGTGATGCAGCACATACATTATGAGGATGAAAACACTCGCTATATATGCATTGGTCCTGTAAACAAGGTG
TTAAATATGCTCTGCTGTTGGGTGGAAGATCCAAATTCCGAAGCGTTCAAGCTGCATCTTCCAAGAATACATGAT
TACCTTTGGCTAGCTGAAGATGGAATGAAAATGCAGGGTTATAATGGGAGTCAACTGTGGGATACAGCTTTTGCA
GTTCAAGCAATCTTGTCCACTAATCTTGTTCAGGAATACGGTCCAACTTTAAAAAAAGCTCATGCTTTTATTAAA
AGTTCTCAGGTCTTAGATGATTGTCACGGTGATCTTGATTTTTGGTATCGTCACATATCAAAAGGTGCATGGCCT
TTTTCAACTGCAGATCATGGTTGGCCAATATCAGACTGCACAGCAGAGGGATTGAAAGCTGCTCTGTTGTTATCC
AAAGTTCCACGCGATATTGTCGGAGATTCATTAGATTTAACCGGTTATGTGATTCAGTCAATGTCATTCTCTCT
CTGCAGAATGGTGATGGCGGATTTGCTACATATGAGCTTACAAGATCTTACAGTTGGTTAGAGTTAATCAATCCT

GCCGAAACTTTTGGTGACATCGTCATCGATTATCCGTACGTAATTATTGGGTTACACCAATTTTCTTTTTCATAA
AAATATCAATTACTAACATGTGCAATCTATGAAGTTATGTCGAGTGTACTTCAGCAGCAGTTCAAGCTCTCACAG
CTTTTAGAAAATTACATCCTGAACATCGACGGGACGAAATAGAAACTTGCATTGAAAAGGCAACCAAGTTTATTG
AGAAAATTCAGGCAGCAGATGGCTCATGGTATGGCTCATGGGGTGTTTGCTTCACCTATGGTACATGGTTTGGCG
TTAAAGGGCTGGTGGCTGCTGGAAAGAACTTTAACAATTGCCCCAGTATTCGAAAGGCTTGTGAGTTTCTGCTGT
CTAAACAGTGTCCTTCTGGTGGTTGGGGAGAGAGTTATCTTTCGTGTCAAACAAAGGTTTATTCCAATCTCGATG
ATAACAGGTCTCATGTTGTAAATACGTCTTGGGCTATGCTGAGCCTTATTGACGCTGGGCAGGTAAATATCGGTT
CTCTATAAGATTGTTTCTTCCGGGTTCATTATCCAGCTCTAATCCCATCTTAATAGCTCAATGATGTGTGATTGC
GGCGACAGTTTGTGCCTGAAAAGAAATATTTTTGTTAGTTTATGATATGCATACTTCCAGCATCTATCAAATATG
CAATGATTCCATTGGTTCCATGCGGGCCACAACTTGTGAACTGTATTAATCACATAATTTGGTTTATTTGTTTTG
AATTTTCCGGGCATTTATGAAGGAAAAGTATTCACTATGTGCTTTCAAAATGCGTAGGCTGAGAGAGACCCAACG
CCATTGCACCGTGCAGCAAGGTACCTGATAAATTCTCAAATGGAAAATGGAGATTTCCCTCAGCAGGTAAATTCA
CCAGTTTCTGACATAATCCACATCTGGAGCTTTTTTATATCTGTGCTGCAAATAATTCATATGTTGGTAATTGTA
GGACTAATTTGACTTTGCTTTCACAAACTGCTTTTCCGAAAAGCCCCATTTCCAAGATTTTGGGGGGAAGTAACT
GGTTTTAGTTTCGGGAAAGCTAACTAGACAATTCTTAACCAATTAGTGTGAATATACAAAATGTGTTGTTTAGGC
CTGTTTGGGATCGATGTTAGAGCTGTTGTCCCTGAAAGTTAACTTTTCAAATAACAACATACTTTGGCCTTTGAT
ACTTGATACAAATAAATTGAGAACTGTTTTGCCACTTTTAAGAAAAGTTTCAAAATGGAGCTTTCGGGGTAAGCA
GTCTGGAAAAGCAATCCAAGATCTAATGTCTTATGAAATGTTTAGTGAGAGCTACTTATCGGGACAAGCATTGAG
ACAACAACACCAACAGCCCTGTGAAGAAGTTTCAGTAGTTGGGGGACTTCGAAATGAGATTTTAGTTCCAATTTA
AGTTGAAATGTCTTTCGATTAATACATGGGAAAATTGAAGGTTTAGTTTTGTGTTAATTGCAGGAAATCATGGGA
GTGTTCAACAAGAACTGCATGATAACATATGCAGCGTACAGAGATATATTCCCAATATGGGCATTGGGAGAGTAT
CGAACCCGGGTACTGCAGCAGTCTTCCTAAGAAAAACACCAAAAATTTTAATATATCTATCGATTTTAGGACTCA
AATTAGTTTCTTTCATTTACTGGAAGATTTAAATAATCTAATTGCAGGCTTGTTAGATTTATGTTTAGATTAATG
CAAACTTGAACAGCACCTTTTCTATAGTATCATTGCTATTGATTTATACTTCTGTGTCATGTAAGCTTCAACGTT
ACAACTCTCCGCGATGAAATGAAAACTTAAGTTTATTTGTT

>PB.2915.1 Euphorbia maculata Triterpene synthase Para.2 mRNA
ATGCATAATTTTCCTCTTTTTCCTCTCCTTTTGCTTTTCGATTGAAGTTGATCTCTAGATTTTGCCTTTTTTTCG
CTGCATTTTGAGTTATAATTGAGTAATAATGCCGATTGCAGCAGTGGAGTGAACAGATCAGAATGTGGAGGTTGA
AGATCGCCGAGGGAGCTGAGAATCCATGGCTGCGGAGTGTTAACAATCATGTTGGAAGACAGGTTTGGGAGTTTG
ATCCTAATCTTGGATCTCCTGAAGATCACTTGCAGATCGAGAATGCTCGTCGGAGTTATCGCGAGAATCGGTTTA
GTATGAAGCATAGTTCAGATCTATTGATGAGGATTCAGTTTGCTAAGGAGAATCCGTTGAGTGAAGTTTTGCCTC
AAGTTAAGCTGAATGATGACGATGATATCACTGAAGAGGCTGTCTCTGTTACTCTGAGGAGAGCTCTGGATTATT
ATTCGACTATTCAGGCACATGATGGCCATTGGCCTGGAGATTATGGAGGCCCTATGTTTCTTATGCCTGGATTGG
TCATAGCTCTATACGTTACTGGAGCACTCAACGCTGTTTTATCAGAGGAGCATAAGAAAGAGATGTGCCGATACC
TATACAACCACCAGAATAGAGATGGTGGATGGGGCTTGCATATTGAGGGCCCGAGCACGATGTTTGGGAGTGTTT
TGTCCTATGTTACTTTGAGATTGTTGGGTGAAGGGGCTAATGATGGAGATGGAGCTATGGAGAGAGGGCGTAAAT
GGATCCTGGACCATGGCAGTGCTACTGCAATCACATCATGGGGGAAAATGTGGCTTTCAGTGCTTGGAGCTTTCG
AGTGGTCTGGGAATAATCCCCTCCCTCCCGAGATATGGCTTCTCCCATATATACTCCCATTCCATCCCGGGAGGA
TGTGGTGCCACTGCCGGATGGTCTATCTGCCCATGTCGTATTTATTTGGAAAAAGGTTTGTTGGCCCAATTACAT
CAACAGTTTTGTCTTTGAGAAAGGAGCTATTCACTGTCCCGTATCATGAAGTAGACTGGAATCATGCACGCAACC
AATGTGCAAAGGAAGACCTGTATTATCCTCATCCCATGGTGCAAGATGTACTTTGGGCAACTCTTGACAAGTTAG
TTGAGCCCATTCTAATGAGTTGGCCTGGAAAAAAGTTGAGAGAAAAGGCTCTTCAGACTGTGATGCAGCACATAC
ATTATGAGGATGAAAACACTCGCTATATATGCATTGGTCCTGTAAACAAGGTGTTAAATATGCTCTGCTGTTGGG
TGGAAGATCCAAATTCTGAAGCGTTCAAGCTGCATCTTCCAAGAATACATGATTACCTTTGGCTAGGTGAAGATG
GAATGAAAATGCAGGGTTATAATGGGAGTCAACTGTGGGATACAGCTTTTGCAGTTCAGGCAATCTTGTCTACTA
ATCTTGCTCACGAATACGGTCCAACTTTAAAAAAGGCTCATGCTTATATTAAAAGTTCTCAGGTCTTGGATGATT
GTCAAGGAGATCTTGATTTTTGGTATCGTCACATCTCAAAAGGTGCATGGCCTTTTTCAACTGCAGATCATGGTT
GGCCAATATCAGATTGCACAGCAGAGGGATTGAAAGCTGCTCTGTTGTTATCCAAAGTTCCACGTGATATTGTCG
GGGATTCATTAGATGTTAACCAGTTATGTGATTCAGTCAATGTCATCCTCTCTCTGCAGAATGGTGATGGTGGAT
TTGCTACATATGAGCTTACAAGATCTTACAGTTGGTTAGAGTTAATCAATCCTGCTGAAACTTTTGGTGACATCG
TCATCGACTATCCTTATGTCGAGTGTACTTCAGCAGCAGTTCAAGCTCTCACAGCTTTTAGAAAATTACATCCCG
AACATCGACGGGACGAAATAGAATCTTGCATTGAAAAGGCAATCAAGTTTATTGAGAAAATTCAGGCAGCAGATG
GCTCATGGTATGGCTCATGGGGTGTTTGCTTCACCTATGGTACATGGTTTGGCGTTAAAGGGCTGGTGGCTGCTG
GAAAGAACTTTAACAATTGCCCCAGTATTCGAAAGGCTTGTGAGTTTCTGCTGTCTAAACAGTGTCCTTCTGGTG
GTTGGGGAGAGAGTTATCTTTCGTGTCAAACAAAGGTTTATTCCAATCTTGACGATAACAGGTCTCATGTTGTAA

ATACTTCTTGGGCTATGCTGAGCCTTATTGATGCTGGGCAGGCTGAGAGAGACCCAACGCCATTGCACCGTGCAG
CAAGGTACCTGATAAATTCTCAAATGGAAAATGGAGATTTCCCTCAGCAGGAAATCATGGGAGTGTTCAACAAGA
ACTGCATGATAACATATGCAGCGTACAGAGATATATTCCCAATATGGGCATTGGGAGAGTATCGAACCCGGGTAC
TGCAGCAGTCTTCGTAAGAAAAACACCAAAAATTTTAATATATTTATCGATTTTAGGACTCAAATTAGTTTCTTT
CATTTACCGGAAGATTTAAATAATCTAATTGCAGGCTTGTTAGATTTATGTTTAGATTAATGCAAACTTGAACAG
CACCTTTTCTATAGTATCATTGTTATTGATTTGTTCTTCTGTGTCATGTACGCTTCAAAGTTACAACTCTTCGGG
ACGAAATGAAAACGCAAGTTTATTTGTG

>PB.2916.1 Euphorbia maculata Triterpene synthase para.3_Iso.1 mRNA
TGCTAACCAAACGTGTATCTCTGCTTCTCGCTCTCTCGTCTGTCACTTTCAAGCTCTCAAGGAACTCGGGAAAGT
GAACGGATCAGAATGTGGAGGTTGAAAATCGCCGAGGGAGCTGAGAATCCATGGCTGCGGAGTGTTAACAATCAT
GTTGGAAGGCAGGTTTGGGAGTTTGATCCTAATCTTGGATCTCCTGAAGATCATTTGCAGATCGAGAATGCTCGT
CAGAGTTTTCGCGAGAATCGGTTGAGTATGAAGCATAGCTCAGATCTATTGATGAGGATTCAGTTTGCGAAGGAG
AATCCGTTGAGTGAAGTTTTGCCGCAAGTTATGCTGAATGACGACGATGCTATCACTGAAGAGGCTGTCTCTGTT
ACTCTGAGGAGAGCTCTGGATTATTATTCGACTATTCAGGCACATGATGGTCATTGGCCTGGAGATTATGGAGGC
CCTATGTTTCTTATGCCTGGATTGGTCATAGCTCTATACGTTACTGGAGCACTCAATGCTGTTTTATCAGAGGAG
CATAAGAAAGAGATGTGTCGATACCTATACAACCACCAGAATAGAGATGGTGGATGGGGCTTGCATATTGAGGGC
CCGAGCACGATGTTTGGGAGTGTTTTGTCCTATGTTACTTTGAGGTTGTTGGGTGAAGGGGCTAATGACGGAGAC
GGAGCTATGGAGAGAGGGCGTAAATGGATCCTGGACCATGGCAGTGCTACTGCAATCACATCATGGGGGAAAATG
TGGCTTTCAGTGCTTGGAGCTTTCGAGTGGTCTGGGAATAATCCCCTCCCTCCCGAGATATGGCTTCTCCCTTAT
ATACTCCCATTCCATCCTGGGAGGATGTGGTGCCACTGCCGGATGGTCTATCTGCCCATGTCATATTTATTTGGA
AAAAGGTTTGTTGGCCCAATTACATCAACAGTTTTGTCTTTGAGAAAGGAGCTATTCACTGTCCCATATCATGAA
GTAGACTGGAATCATGCACGCAACCAATGTGCAAAGGAAGACCTGTATTATCCTCATCCCATGGTGCAAGATGTA
CTTTGGGCAACTCTTGACAAGTTAGTTGAGCCCATTCTAATGAGTTGGCCTGGAAAAAAGTTGAGAGAAAAGGCT
CTTCAGACTGTGATGCAGCACATACATTATGAGGATGAAAACACTCGCTATATATGCATTGGTCCTGTAAACAAG
GTGTTAAATATGCTCTGCTGTTGGGTGGAAGATCCAAATTCCGAAGCGTTCAAGCTGCATCTTCCAAGAATACAT
GATTACCTTTGGCTAGCTGAAGATGGAATGAAAATGCAGGGTTATAATGGGAGTCAACTGTGGGATACAGCTTTT
GCAGTTCAAGCAATCTTGTCCACTAATCTTGTTCAGGAATACGGTCCAACTTTAAAAAAAGCTCATGCTTTTATT
AAAAGTTCTCAGGTCTTAGATGATTGTCACGGTGATCTTGATTTTTGGTATCGTCACATATCAAAAGGTGCATGG
CCTTTTTCAACTGCAGATCATGGTTGGCCAATATCAGACTGCACAGCAGAGGGATTGAAAGCTGCTCTGTTGTTA
TCCAAAGTTCCACGCGATATTGTCGGAGATTCATTAGATTTTAACCGGTTATGTGATTCAGTCAATGTCATTCTC
TCTCTGCAGAATGGTGATGGCGGATTTGCTACATATGAGCTTACAAGATCTTACAGTTGGTTAGAGTTAATCAAT
CCTGCCGAAACTTTTGGTGACATCGTCATCGATTATCCTTATGTCGAGTGTACTTCAGCAGCAGTTCAAGCTCTC
ACAGCTTTTAGAAAATTACATCCTGAACATCGACGGGACGAAATAGAAACTTGCATTGAAAAGGCAACCAAGTTT
ATTGAGAAAATTCAGGCAGCAGATGGCTCATGGTATGGCTCATGGGGTGTTTGCTTCACCTATGGTACATGGTTT
GGCGTTAAAGGGCTGGTGGCTGCTGGAAAGAACTTTAACAATTGCCCCAGTATTCGAAAGGCTTGTGAGTTTCTG
CTGTCTAAACAGTGTCCTTCTGGTGGTTGGGGAGAGAGTTATCTTTCGTGTCAAACAAAGGTTTATTCCAATCTC
GATGATAACAGGTCTCATGTTGTAAATACGTCTTGGGCTATGCTGAGCCTTATTGACGCTGGGCAGGCTGAGAGA
GACCCAACGCCATTGCACCGTGCAGCAAGGTACCTGATAAATTCTCAAATGGAAAATGGAGATTTCCCTCAGCAG
GAAATCATGGGAGTGTTCAACAAGAACTGCATGATAACATATGCAGCGTACAGAGATATATTCCCAATATGGGCA
TTGGGAGAGTATCGAACCCGGGTACTGCAGCAGTCTTCCTAAGAAAAACACCAAAAATTTTAATATATCTATCGA
TTTTAGGACTCAAATTAGTTTCTTTCATTTACTGGAAGATTTAAATAATCTAATTGCAGGCTTGTTAGATTTATG
TTTAGATTAATGCAAACTTGAACAGCACCTTTTCTATAGTATCATTGCTATTGATTTATACTTCTGTGTCATGTA
AGCTTCAACGTTACAACTCTCCGCGATGAAATGAAAACTTAAGTTTATATGTG

>PB.2916.2 Euphorbia maculata Triterpene synthase para.3_Iso.2 mRNA
GGCAACTCTTGACAAGTTAGTTGAGCCCATTCTAATGAGTTGGCCTGGAAAAAAGTTGAGAGAAAAGGCTCTTCA
GACTGTGATGCAGCACATACATTATGAGGATGAAAACACTCGCTATATATGCATTGGTCCTGTAAACAAGGTGTT
AAATATGCTCTGCTGTTGGGTGGAAGATCCAAATTCCGAAGCGTTCAAGCTGCATCTTCCAAGAATACATGATTA
CCTTTGGCTAGCTGAAGATGGAATGAAAATGCAGGGTTATAATGGGAGTCAACTGTGGGATACAGCTTTTGCAGT
TCAAGCAATCTTGTCCACTAATCTTGTTCAGGAATACGGTCCAACTTTAAAAAAAGCTCATGCTTTTATTAAAAG
TTCTCAGGTCTTAGATGATTGTCACGGTGATCTTGATTTTTGGTATCGTCACATATCAAAAGGTGCATGGCCTTT
TTCAACTGCAGATCATGGTTGGCCAATATCAGACTGCACAGCAGAGGGATTGAAAGCTGCTCTGTTGTTATCCAA
AGTTCCACGCGATATTGTCGGAGATTCATTAGATTTTAACCGGTTATGTGATTCAGTCAATGTCATTCTCTCTCT
GCAGAATGGTGATGGCGGATTTGCTACATATGAGCTTACAAGATCTTACAGTTGGTTAGAGTTAATCAATCCTGC
CGAAACTTTTGGTGACATCGTCATCGATTATCCTTATGTCGAGTGTACTTCAGCAGCAGTTCAAGCTCTCACAGC

TTTTAGAAAATTACATCCTGAACATCGACGGGACGAAATAGAAACTTGCATTGAAAAGGCAACCAAGTTTATTGA
GAAAATTCAGGCAGCAGATGGCTCATGGTATGGCTCATGGGGTGTTTGCTTCACCTATGGTACATGGTTTGGCGT
TAAAGGGCTGGTGGCTGCTGGAAAGAACTTTAACAATTGCCCCAGTATTCGAAAGGCTTGTGAGTTTCTGCTGTC
TAAACAGTGTCCTTCTGGTGGTTGGGGAGAGAGTTATCTTTCGTGTCAAACAAAGGTTTATTCCAATCTCGATGA
TAACAGGTCTCATGTTGTAAATACGTCTTGGGCTATGCTGAGCCTTATTGACGCTGGGCAGGCTGAGAGAGACCC
AACGCCATTGCACCGTGCAGCAAGGTACCTGATAAATTCTCAAATGGAAAATGGAGATTTCCCTCAGCAGGAAAT
CATGGGAGTGTTCAACAAGAACTGCATGATAACATATGCAGCGTACAGAGATATATTCCCAATATGGGCATTGGG
AGAGTATCGAACCCGGGTACTGCAGCAGTCTTCCTAAGAAAAACACCAAAAATTTTAATATATCTATCGATTTTA
GGACTCAAATTAGTTTCTTTCATTTACTGGAAGATTTAAATAATCTAATTGCAGGCTTGTTAGATTTATGTTTAG
ATTAATGCAAACTTGAACAGCACCTTTTCTATAGTATCATTGCTATTGATTTATACTTCTGTGTCATGTAAGCTT
CAACGTTACAACTCTCCGCGATGAAATGAAAACTTAAGTTTATTTGCG

>PB.12605.1 Euphorbia maculata Squalene synthase Para.1 mRNA
AGGCGCAATAAATCCTTCCCTTTGGACTTTCCTCGTCTTCAACGGAAAATCCCCATAGCCAATTCTTTCTTTCTT
CTTTATTATATATTCTCCTTCAATGCCAGCCAGCCACCCGTCTCTGTATCTTTTGTGACAGATATTGTTCAATCT
CTGTTCGATTCCATCGTGTTCCTTTCGCAAATCGGTTGATTTTGATCTTTCGCGCTCGTTATCGGGATCAATTTG
ATTTCGATTTGGGGTTTCTAGGTATCTGTGAGTGATGGGGAGTTTGGGCGCGATTCTTCGACATCCGGATGATTT
CTACCCGCTTCTGAAGCTGAAAATGGCTTCGAAGCACGCGGAGAAGCAGATCCCGCCGCAGCCGAATTGGGGGTT
TTGTTATTCGATGCTTCATAAGGTCTCTCGGAGCTTTGCGCTCGTGATTCAGCAGCTTAACACTGAGCTCCGTGA
CGCTATCTGTATTTTCTATTTGGTTCTGAGAGCCCTTGATACCGTTGAGGATGATACAAGCATCGCTACAGATGT
GAAAGTGCCTATCTTGATAGCTTTTCACAAGCACCTATATGACCCGGAATGGCATTTTTCTTGTGGTACAAAGGA
ATATAAGGTTCTGATGGATCAGTTTCATCATGTTTCAACTGCTTTTCTCGAGCTCGGGAAAAGTTATCAGGAGGC
AATTGAGGATATCACAAAAAGAATGGGTGCAGGAATGGCTAAATTCATTTGCAAAGAGGTGGAAACAGTTGATGA
CTATGATGAATATTGCCACTATGTGGCAGGGCTTGTTGGATTAGGCCTTTCCAAGCTTTTTGATGCTTCCGGGTT
TGAAGATTTAGCACCAGATAACCTGTCTAACTCCATGGGGTTATTTCTTCAGAAAACAAACATAATCAGGGATTA
TTTGGAGGATATAAATGAGATACCTAAGTCACGAATGTTTTGGCCTCGCCAGATATGGAGTAAATATGTCAATAA
GCTTGAGGACTTAAAATATGAAGAAAATTCAGTCAAGGCAGTGCAATGCTTGAATGATATGGTTACTAATGCGTT
GATACATATGGATGATTGCTTGAAATACATGGCTGCACTTCGAGATCCTTCTATATTTCGATTTTGTGCCATTCC
TCAGATCATGGCAATTGCAACCCTAGCATTGTGCTACAACAACATTGAAGTATTTAGAGGAGTAGTGAAGATGAG
GCGTGGTCTGACTGCAAAGGTCATTGACCGTACAAGAACAATGGCAGATGTCTATAGAGCCTTCTATGACTTTGC
TGGTATGATGAAAGCCAAGGTCGACATGAATGATCCTAATGCAGAAAAGACACTAAATCGGCTCGAAGCAGTACA
AAAAACTTGCATGGAATCTGGTGTACTGAACACAAGGAAATCTTACATAGATGGGAGCGGACCGAAATATAACTC
TGTTCTGGTTGTTCTTCTGATTATCGTGTTGGCTATTATCTTGTCTTATCTCAGAAAACAGACCAACTAGTGTAA
GTTCGGTCACAAAATTGATTTGAATTTCGGGGTGCCGACATCTACGAGTATTATCTGCGGCTTTTGCATTTTCAG
TGCTCGGTACGATCAAGGATATGGGATTTCTGGTGTTCTGTATTTAGTGTAATGGCATAACAATATTTATGTCCA
AATTTCGATTGTGTTTCGGCTGAGTGTAAGCATATACTATGACTTCTCATTGTTGTCTGGTATTGTATACTCTAT
TTATGGAAATGAAATAATATTGTGATATTGAAGGTTCTAAGTG

>PB.12606.1 Euphorbia maculata Squalene synthase Para.2 mRNA
AACGGAAAATCCCCAAATCGCATACTTCAACCTTTCTTTACTATATATTTCCCTTCATTGCCAGCCAGCCACACG
TCTCTGTATCTTCTGTGACAGATATTGTTCAATCTCTGTCAATTCTGTCGTTTTCGTTTCAAGCCGATTCATAAA
TTGTTTGATTTTGATCTTTCGTGATCGTTGTTGGAGTTAATTTGATTTTGGCGTGGGCGATTTGGGGTTTCGAGG
GATCTGTGAGTAATGGGGAGTTTGGGAGCGATTCTGAGGCATCCGGATGATTTCTACCCGCTTTTGAAGCTGAAA
ATGGCTTCCAAGCATGCGGAGAAGCAGATCCCGCCACAGCCGAATTGGGGGTTTTGTTATTCGATGCTGCATAAG
GTGTCTCGGAGCTTTGCGCTGGTGATTCAGCAGCTCAACACTGAGCTCCGTGACGCTGTCTGTATTTTCTATTTG
GTTCTGAGAGCCCTTGATACTGTCGAGGATGATACAAGCATCGCTACTGATGTGAAAGTGCCTATCTTGATAGCT
TTTCACAAGCACCTATATGATCCGGAATGGCATTTTTCTTGTGGTACAAAGGAATATAAAGTTCTGATGGACCAG
TTTCATCATGTTTCAACTGCTTTTCTTGAGCTTGGGAAAAGTTATCAGGAGGCAATTGAGGATATCACAAAAAGA
ATGGGTGCAGGAATGGCCAAATTCATATGCAAAGAGGTGGAAACAGTTGATGACTACGATGAATATTGCCATTAT
GTGGCAGGGCTTGTGGGATTAGGTCTTTCCAAGCTTTTTGAGGCCTCCGGGTTTGAAGATTTAGCCCCAGATAAC
CTGTCTAATTCCATGGGTTTATTTCTTCAGAAAACGAACATAATCAGGGATTATTTGGAGGATATAAATGAGATA
CCTAAGTCACGAATGTTTTGGCCTCGCCAGATATGGAGTAAATATGTCAATAAGCTTGAGGACTTAAAATATGAA
GAAAATTCAGTCAAGGCAGTTCAATGCTTGAATGATATGGTTACTAATGCATTGATACATATGGATGATTGCTTG
AAATACATGGCTGCACTGCGGGATCCTTCAATATTTCGATTTTGCGCCATCCCTCAGATCATGGCAATTGCTACC
CTAGCATTGTGCTACAACAACATTCAAGTGTTTAGAGGTGTAGTGAAGATGAGGCGTGGTCTTACTGCAAAGGTC
ATTGATCGTACAAGAACTATGGCAGATGTGTATAGATCCTTCTATGACTTTGCTGGTATGATGAAAGCCAAGGTT

GATATGAATGATCCTAATGCCGAAAAGACACTAAATCGGCTCGAAGCAGTACAAAAAACTTGCAAGGAATCTGGT
GTACTAAACACAAGGAAATCTTACATAGATGGGAGCGGGCCGAAGTATAACTCTGTTCTGGTTGTTCTTCTGATT
ATTGCATTGGCTATTTACTTGTCTTATCTCAGAAAACAGACCAACTAGTGTAAGTTTGGTCAAAAGCGCCGACTT
CTATGAGTATTTTTTTCTGTGGTTTTTGCATTTTCAGTGCTCGGTACGTTCTAGGAGATGGGATTTCTTGTGTTCT
GTATTTAGTGTAATGGCTTAACAATATTTATGTCCAAATTTCAATTGCATTTTGGTATTTTATGTCTCGGGTGAG
TGTAAACATACTAATGATTTCTCATTGTTGTTTGGTATTGTATACTCTATTTATGGAAATGAAATAATATTGTGA
T
